# SeQuery: an interactive graph database for visualizing the GPCR superfamily

**DOI:** 10.1093/database/baz073

**Published:** 2019-06-25

**Authors:** Geng-Ming Hu, M K Secario, Chi-Ming Chen

**Affiliations:** 1 Department of Physics, National Taiwan Normal University, 88 Sec. 4 Ting-Chou Rd., Taipei 11677, Taiwan; 2 Department of Applied Chemistry, National Chiao Tung University, 1001 Ta Hsueh Rd., Hsinchu 300, Taiwan

## Abstract

The rate at which new protein and gene sequences are being discovered has grown explosively in the omics era, which has increasingly complicated the efficient characterization and analysis of their biological properties. In this study, we propose a web-based graphical database tool, SeQuery, for intuitively visualizing proteome/genome networks by integrating the sequential, structural and functional information of sequences. As a demonstration of our tool’s effectiveness, we constructed a graph database of G protein-coupled receptor (GPCR) sequences by integrating data from the UniProt, GPCRdb and RCSB PDB databases. Our tool attempts to achieve two goals: (i) given the sequence of a query protein, correctly and efficiently identify whether the protein is a GPCR, and, if so, define its sequential and functional roles in the GPCR superfamily; and (ii) present a panoramic view of the GPCR superfamily and its network centralities that allows users to explore the superfamily at various resolutions. Such a bottom-up-to-top-down view can provide the users with a comprehensive understanding of the GPCR superfamily through interactive navigation of the graph database. A test of SeQuery with the GPCR2841 dataset shows that it correctly identifies 99 out of 100 queried protein sequences. The developed tool is readily applicable to other biological networks, and we aim to expand SeQuery by including additional biological databases in the near future.

## Introduction

In recent years, the number of new genomic and proteomic sequences being produced in laboratories has increased by several orders of magnitude (1, 2). This explosion of new sequences produces a demand for methods capable of efficiently characterizing these sequences and of synthesizing this information into useful knowledge in the domains of biological complexity and human medicine. To extract such knowledge from large quantities of experimental data, new methods in analytics and bioinformatics are being developed to search for correlations between the evolutionary histories, structures and functions of protein sequences (3–6). Computational algorithms have also been developed for integrating various genomic and proteomic data sources to better understand rigorously regulated cellular processes (7, 8). With the help of more advanced computational techniques and the general availability of high-bandwidth networking, the sharing of data in genomics and proteomics will likely play a significant role in current explorations of the big picture of life.

Various public repositories of genomic and proteomic data have been established to fulfill different purposes. Proteomic analyses are usually more complex than genomic analyses, and original proteomic data are less frequently described and stored in a systematic way. Repositories of proteomic sequences can be classified into three categories: (i) raw data repositories, (ii) peptide/protein identification and quantification repositories and (iii) protein knowledge bases (9–11). Here, we focus on three databases in the third category: UniProt, RCSB PDB and GPCRdb. UniProt is an important hub of protein information that cross-references >150 databases. Currently, it is comprised of ~0.6 million reviewed sequences and 116 million annotated but unreviewed sequences (12). The RCSB PDB is an archive of experimentally determined, atomic-level 3D structures of biological macromolecules. In all, it collects >44 000 distinct structures of protein sequences and 10 000 structures of nucleic acid compounds (13). GPCRdb is an information system for G protein-coupled receptors (GPCRs), which contains data, diagrams and web tools for GPCRs. It contains information concerning >14 000 proteins from 3547 species (14). Other public databases provide additional GPCR data (9). Most of these databases provide detailed item-by-item descriptions of proteins, but they all lack intuitive, panoramic representations of the GPCR superfamily as well as the complex relationships between proteins. Therefore, by proper analysis and integration of GPCR data from existing repositories, we aim to construct a graphical database of GPCRs that allows users to intuitively and interactively explore the GPCR superfamily while visualizing its high-level structure and complexity. These techniques can also be extended to document other biological and medical systems.

GPCRs are the largest protein superfamily encoded by mammalian genomes. They share a common counter-clockwise bundle structure of seven transmembrane (TM) helices associated with heterotrimeric G proteins (15). Upon ligand binding, the conformational changes of GPCRs activate the G protein to allosterically modulate the activities of various downstream effector proteins; they regulate a wide variety of physiological functions, including smell, taste, vision, secretion, neurotransmission, metabolism, cellular differentiation and growth, and inflammatory and immune responses (16–18). Consequently, malfunctions in GPCR signaling pathways can cause various diseases, including cancer, diabetes, obesity, inflammation, cardiac dysfunction and central nervous system disorders. An increasing number of analyses have linked the abnormal expression of GPCRs and their autocrine/paracrine activation by agonists to various types of maladies in humans. For instance, it has been experimentally demonstrated that many GPCRs could function as biomarkers for the early diagnosis of cancer, and the pharmacological inhibition of GPCRs could interrupt cancer progression and metastasis (2, 19, 20). Therefore, GPCRs play a crucial role in developing a strategy for cancer prevention and treatment. The clinical importance of GPCRs is further demonstrated by their current pharmaceutical applications; ~34% of the Food and Drug Administration (FDA)-approved drugs affect GPCRs, and ~20% of drugs for which clinical trials were performed in 2017 collectively target 66 GPCRs that currently have no approved drugs (21). Understanding the structure, functions and therapeutic antibodies of the remaining GPCRs, particularly the ~120 orphan GPCRs whose ligands are currently unknown (22), could fuel the advance in GPCR-based drug discovery.

Due to the diverse roles of GPCRs in cellular regulation and signal transduction, the proper identification and classification of GPCRs are crucial to understanding their biological and pharmaceutical applications (23, 24). By integrating GPCR data from UniProt, GPCRdb and RCSB PDB and applying analytical methods such as minimum span clustering (MSC) method and graph centrality, we constructed a web-based graphical database, SeQuery (http://cluster.phy.ntnu.edu.tw), which allows users to efficiently identify GPCR sequences and to intuitively visualize their sequence, structure, function and centrality relationships in the GPCR sequence similarity network. We classify our dataset of 2841 GPCRs at three characteristic resolutions in SeQuery based on MSC results and functional annotations for sequences from GPCRdb and UniProt. Users can classify a newly discovered GPCR by comparing its sequence with those in SeQuery’s GPCR dataset. A test of SeQuery with the GPCR2841 dataset shows that it correctly identifies 99 out of 100 queried protein sequences and that it can provide a bottom-up visual exploration of the sequence similarity network by contextualizing the structural and biological properties of individual GPCR sequences. SeQuery also offers a top-down visual exploration of the GPCR superfamily, which shows the sequence/function relationships of GPCRs at the three resolution levels that we identified. For each functional GPCR family, SeQuery presents graphical views and centrality measures. Currently, SeQuery does not support the bulk insertion of a large number of GPCR sequences for computer-aided analyses; however, users can use the methods that we describe in the following section to analyze large sets of sequences.

## Materials and methods

### Dataset preparation

For this study, 3105 reviewed GPCR sequences were retrieved from UniProt in December 2018. We first searched UniProt with the query string ‘GPCR AND reviewed:yes’ and downloaded data of all 3653 matches. From the downloaded data, we then used MATLAB to isolate 3145 sequences by searching ‘G-protein coupled receptors’ in ‘Keywords’ or ‘G-protein coupled receptor’ or ‘G protein-coupled receptor’ in ‘Protein Names’. After verifying these sequences against published literature, we obtained a set of 3105 GPCR sequences. Among this set, 2841 GPCR sequences (dataset GPCR2841) were used to construct SeQuery’s interactive graph database; the remaining sequences were used to test the validity of SeQuery in identifying GPCR sequences. These GPCR sequences originate from >300 organisms and contain both orthologs and paralogs (25). According to the annotations in GPCRdb and UniProt, the dataset contains 2297 class A receptors (rhodopsin-like), 182 class B receptors (secretin-like), 68 class C receptors (metabotropic glutamate), 40 class D receptors (vomeronasal), 172 type 2 taste receptors (T2R), 4 class E receptors (cAMP) and 78 frizzled receptors (26). In the Supporting Information, Supplementary data Table S1 shows the MSC cluster label, UniProt ID and GPCRdb labels of each sequence. Every MSC cluster is labeled by the first two or three alphanumeric characters of the receptor group, followed by a three-digit number. We note that the proposed graph database needs to be rebuilt when the dataset is changed significantly, since the calculated distance matrix from BLASTp and the detailed network properties of the dataset may be altered by the addition of new sequences. An expanded GPCR3105 dataset containing 3105 reviewed sequences will be released in the near future.

**Figure 1:**
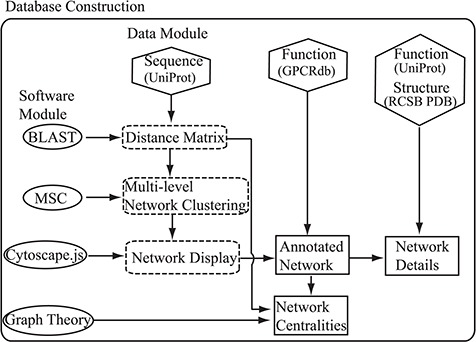
Flowchart of the construction of the SeQuery. Hexagons denote the source databases, and ovals denote computational methods. Intermediate data derived during computation is represented by dashed squares, while the generated graphs for visualization are represented by solid squares.

**Figure 2:**
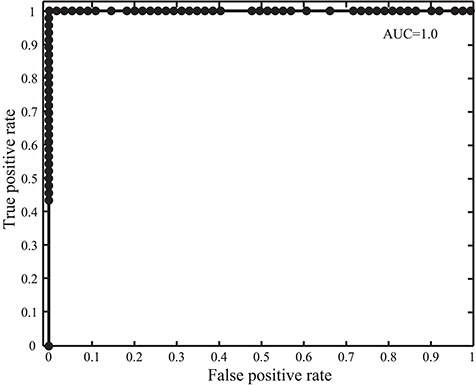
Receiver operating characteristic and AUC in the GPCR detection with SeQuery for the 100 tested protein sequences.

## Methods

As shown in Figure 1, SeQuery’s interactive graph database for the GPCR superfamily is constructed based on two modules: the data module, which consists of the sequence, function and structure information of GPCRs retrieved from UniProt, GPCRdb and RCSB PDB; and the software module, which contains BLAST, MSC, Cytoscape.js and graph-theoretic methods. Future expansions will incorporate more biological data in the data module. To construct the database, the sequence data are first analyzed by BLASTp to generate the distance matrix for the sequence similarity network of GPCRs. This distance matrix is analyzed using MSC to cluster the GPCR superfamily at three resolution levels. The cluster information is then provided as input to Cytoscape.js to display graphs of the GPCR clusters at these resolutions. Additionally, the functional classification of GPCRdb is used to annotate the GPCR sequences in each graph, so as to demonstrate the relationship between the sequences and functions of GPCRs in the sequence similarity network. We also add functional information from UniProt and known structures from RCSB PDB to each graph. Finally, we calculate various centrality measures of the sequence similarity network by graph-theoretic methods and display graphs of each GPCR family for different node-pair distance threshold values. The computations performed by SeQuery are detailed in the following paragraphs.

**Figure 3:**
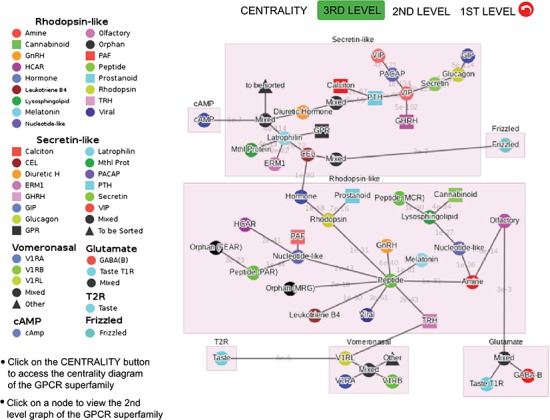
Third-level minimum spanning tree diagram of the GPCR superfamily with the base dataset. Each node represents a GPCR family. The legend shows the scheme of nodes’ colors and shapes that is used to distinguish GPCR functions annotated in GPCRdb (also labeled on the nodes).

BLASTp, using the general scoring matrix BLOSUM62 with default parameters (27), is used to calculate the distance matrix of the network based on the GPCR sequence data. We define the symmetrized sequence distance between protein sequences *i* and *j* as ${d}_{i,j}=\sqrt{E_{i,j}{E}_{j,i}}$, where *E*_*i*,*j*_ is the BLASTp *E*-value, a parameter that describes the expected number of matches due to chance when searching for the best-aligned region between sequences *i* and *j* in a database of a particular size. This definition of sequence distance is not unique, and other definitions have also yielded informative predictions in analyses of protein networks (4, 28). For detecting distant relationships between protein sequences, we also employ the PSI-BLAST (Position-Specific Iterative Basic Local Alignment Search Tool) algorithm, which iteratively uses an updated position-specific scoring matrix to search the dataset for new matches (29). We similarly define the distance between distant sequences as ${d}_{i,j}^{\prime }=\sqrt{E_{i,j}^{\prime }{E}_{i,j}^{\prime }}$, where ${E}_{i,j}^{\prime }$ is the PSI-BLAST *E*-value.

**Figure 4:**
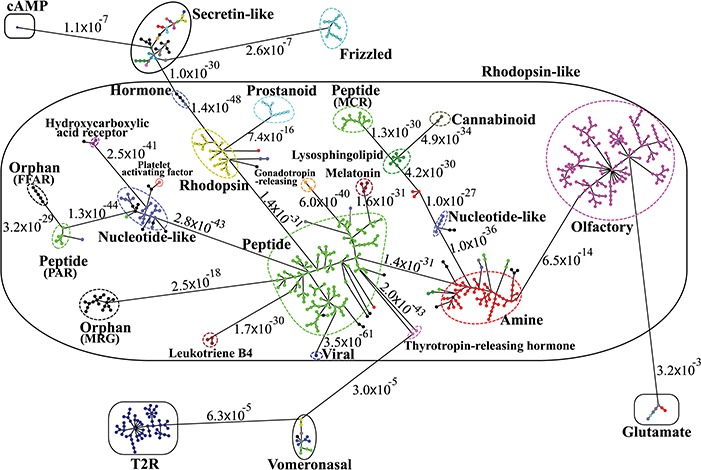
Minimum spanning tree diagram of the GPCR network in the dataset GPCR2841 (outliers not shown). Each circle represents an MSC cluster, which is colored according to the functions of its constituents. The lengths of the edges are not proportional to their distances, but the distances between subfamilies and classes are labeled to visualize their sequence similarities.

We cluster the GPCR superfamily by applying MSC to the distance matrix $\big(\big\{{d}_{i,j}\big\}\big)$. MSC provides a hierarchical approach to clustering and visualizing the structure of a complex network at various resolution levels. It does not require hyper-parameterization nor *a priori* knowledge of the number of clusters and outperforms other clustering algorithms in efficiency and accuracy in the clustering of large networks. These attributes make MSC an ideal tool for network analysis in a large web-based database. A more detailed description of the MSC algorithm is available in our previous work (3).

In our previous analysis, the MSC clustering of protein sequences exhibited a disparity in sequence distances between GPCR-GPCR pairs and GPCR-non GPCR pairs (3). Here, we assume that a protein sequence is a GPCR if its shortest distance to GPCR sequences in the base dataset is smaller than a threshold of 0.0009, which maximizes the *F*-measure of identifying GPCRs to be 0.9998. In SeQuery, we utilize this assumption to assess if a query sequence (not in the base dataset) is a GPCR. For simplicity, the distances between the query sequence and the base sequences are calculated using the base dataset instead of the base + query dataset. This simplification could in principle lead to a small deviation in calculated sequence distances due to a change in the dataset size (1/2842). To demonstrate this conjecture, we examined the effect of a small size change by calculating the distances of the 2841 base sequences to 10 query sequences with lengths ranging from 132 to 4568 amino acids (a.a.), listed in Supplementary data Table S2. For all query sequences, we defined the normalized root mean square deviation (NRMSD) of its distances to the 2841 base sequences as $\mathrm{NRMSD}=\sqrt{\frac{1}{2841}{\sum}_{i=1}^{2841}{\Big(1-{d}_i^{\mathrm{b}}/{d}_i^{\mathrm{b}+\mathrm{q}}\Big)}^2}$, where ${d}_i^{\mathrm{b}}$ and ${d}_i^{\mathrm{b}+\mathrm{q}}$ are the calculated sequence distances to sequence *i* using the base dataset and using the base + query dataset, respectively. As shown in Supplementary data Table S2, the value of NRMSD is less than 2 × 10^−5^ for all queried sequences, suggesting that ${d}_i^{\mathrm{b}}$ is a valid approximation of ${d}_i^{\mathrm{b}+\mathrm{q}}$. We also note that longer query sequences affect the NRMSD more strongly.

For interactive visualization of GPCRs, clusters and network graphs based on MSC clustering results are prebuilt and presented using the JavaScript library Cytoscape.js (30). These graphs illustrate the GPCR superfamily at three different resolution levels; the first level shows the relationships among receptor sequences, the second level shows the relationships among receptor clusters and the third level shows the relationships among receptor families.

Nodes with high centrality are highly involved in the structure of a network. To evaluate the centrality of important nodes in the GPCR sequence similarity network, SeQuery uses four different centrality measures, namely the weighted degree (*C*_WD_), closeness (*C*_C_), betweenness (*C*_B_) and eigenvector (*C*_EV_) centralities. We consider an all-to-all, undirected, weighted graph *G*:= (*V*, *E*) with |*V*| nodes and |*E*| edges; the weight matrix **W** of *G* has weights *w_uv_* for the edge connecting each pair of nodes (*u*, *v*), $\forall u,v\in V$. Equivalently, we can define a distance matrix **D** for *G* with elements $\tilde{d}$*_uv_*, where $\tilde{d}$*_uv_* ≡ ${w}_{uv}^{-1}-1$. To calculate the centrality measures for the GPCR network and avoid numerical errors, we consider the relation $\tilde{d}$*_uv_* ≡ $d$*_uv_*^0.01^ + δ, where δ = 10^−200^ is an arbitrarily small distance and *d*_uv_ are the sequence distance matrix elements for GPCR pairs (*u*, *v)*, $\forall u,v\in V$. Diagonal elements in both **W** and **D** have a value of 0. For *G*, the weighted degree centrality of a node $u\in V$ is defined as

(1) \begin{equation*} {C}_{\mathrm{WD}}(u)=\sum_{v\in V}{w}_{uv}.\kern0.5em \end{equation*}

The closeness centrality of a node $u\in V$ is defined as

(2) \begin{align*}{C}_{\mathrm{C}}(u)=\left(\left|V\right|-1\right)\cdot {\left[\sum_{v\in V}\tilde{d}\left(u,v\right)\right]}^{-1},\end{align*}

where $\tilde{d}(u,v)$ is the shortest distance between nodes *u* and *v*. The betweenness centrality of a node $u\in V$ is defined as

(3) \begin{equation*}{C}_B(u)=\sum_{\begin{subarray}{c}j,k\\ {}j\ne k\ne u\end{subarray}}\frac{g_{jk}(u)}{g_{jk}},\end{equation*}

where *j* and *k* are other nodes in the network such that *j* ≠ *k* ≠ *u*, *g_jk_* is the number of shortest paths between node *j* and node *k* and *g_jk_* (*u*) is the number of those paths that pass through the node *u*. The definition of betweenness centrality of a node in Equation (3) can be extended to calculate the betweenness centrality of an edge by calculating *g_jk_* (*e*), the number of shortest paths that pass through the edge *e*. The eigenvector centrality of a node $u\in V$ is defined as

(4) \begin{equation*}{C}_{\mathrm{EV}}(u)=\frac{1}{\lambda}\sum_{v\in V}{w}_{uv}{C}_{\mathrm{EV}}(v), \end{equation*}

where λ is an eigenvalue. This equation can be rewritten in vector notation as the eigenvector equation **Wx** = λ**x**, where *x_u_* = *C*_EV_(*u*). As the elements of **W** are nonnegative, there is a unique largest eigenvalue, which is real and positive. The eigenvector **x** corresponding to this eigenvalue yields the desired centrality measure.

In studying the sequence similarity network of GPCRs, we compute and interpret the above centrality indices to characterize important nodes or edges within the network. The weighted degree centrality ${C}_{\mathrm{WD}}(u)$ of node *u* is used to characterize its overall connectivity to other sequences in the network. The closeness centrality ${C}_{\mathrm{C}}(u)$ of a node *u* measures the reciprocal of the sum of the length of the shortest paths between *u* and all other nodes; the more central a node is, the closer it is to all other nodes. In graph theory, the eigenvector centrality is a measure of the influence of a node in a network; a high eigenvector centrality means that a node is connected to many other nodes of high centralities. Lastly, the betweenness centrality of a node or edge is the number of the shortest paths that pass through it; a node or edge with high betweenness centrality exerts more control over the network. In general, the closeness, weighted degree and eigenvector centralities have similar patterns in a complex network, while the betweenness centrality fundamentally differs from the other measures and represents the dynamic information flow of the network (31). For our study of GPCRs, the first three centrality measures can be used to find the most representative or influential sequences in a GPCR cluster, sub-family or family. The betweenness centrality can be used to find sequences (nodes) or sequence pairs (edges) that bridge different domains in the sequence space of GPCRs; these sequences or sequence pairs could play a key role in the evolution of GPCRs.

**Figure 5:**
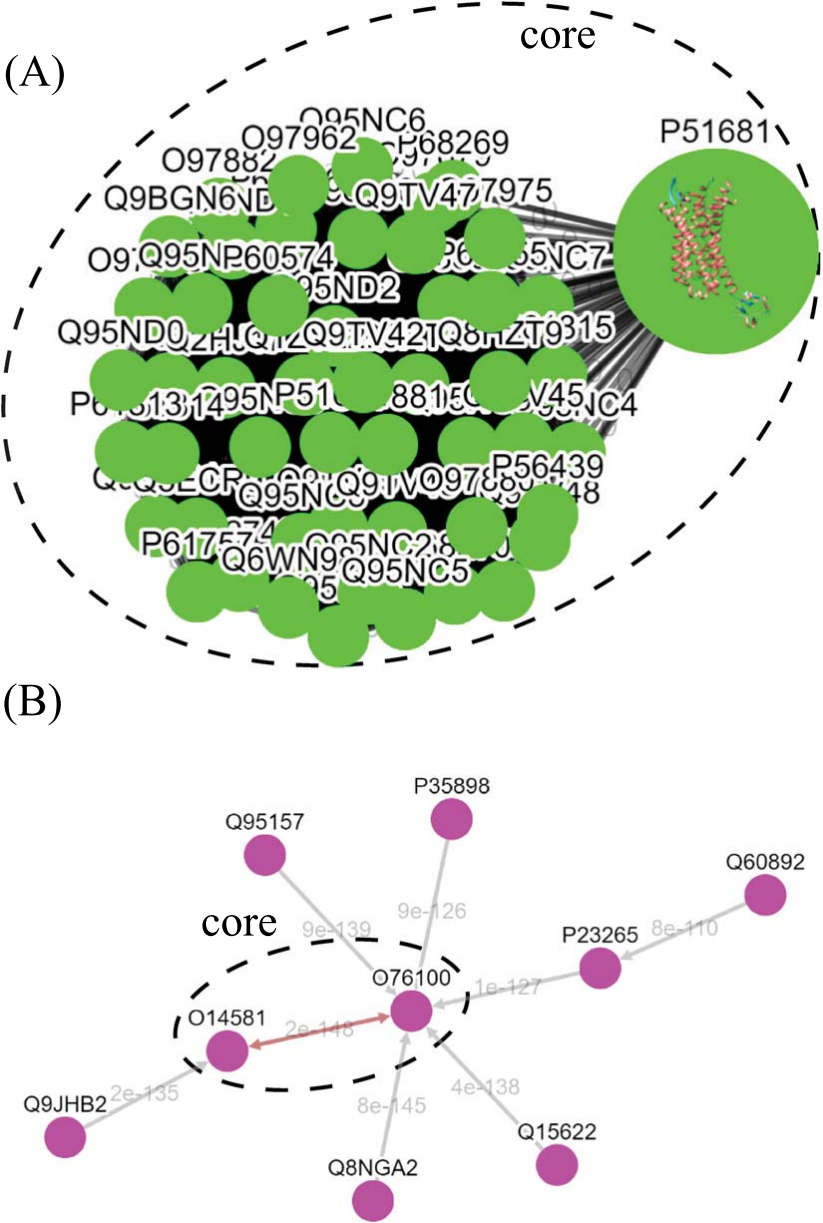
Tree diagrams of the first-level GPCR clusters, showing member sequences of cluster Pe001, which has a conservative core (**A**); and member sequences of cluster Ol001, which has no conservative core (**B**). In (A), the 3D protein structure of P51681 is displayed in its node, and a line segment is used to represent a zero-distance edge connecting two nodes in the core. In (B), a double-headed arrow is used to represent the shortest edge connecting two nodes in the core, and a single-headed arrow (directed toward the node closer to the core) is used to represent all other edges connecting two nodes in the cluster. Each edge is labeled with its length.

## Results and discussion

We intend SeQuery to be a web-based graph database for understanding complex proteome and genome networks. To demonstrate this functionality, we implemented SeQuery as an interactive graph database of GPCRs, which identifies possible GPCR sequences and offers a bottom-up-to-top-down panoramic view of the GPCR superfamily. To verify the ability of SeQuery to identify GPCR sequences, we submitted 100 query sequences randomly selected from a test dataset (the probability of selecting a GPCR sequence is 0.5), which included 46 newly reviewed GPCR sequences and 54 non-GPCR proteins. Among the randomly chosen non-GPCR proteins, 25 sequences were membrane proteins with chain lengths from 132 a.a. to 731 a.a., and 29 sequences were 7TM non-GPCRs with chain lengths from 232 to 627 a.a. 7TM non-GPCR sequences have the same 7TM helix topology as GPCRs but do not couple to G proteins. Soluble proteins were not considered in this test since they can be easily distinguished by secondary structure prediction tools such as TMHMM (32). Supplementary data Table S3 provides detailed information regarding the selected test sequences. Figure 2 shows the receiver operating characteristic curve that we constructed to test our GPCR predictor; we utilize the area under the curve (AUC) to evaluate its predictive ability (0.5 for a random predictor and 1 for an excellent predictor). The validity of our method is supported by its AUC value of 1.0, which would be lowered by the presence of remote GPCR homologs or sequences from a novel GPCR class in the test dataset. A close examination of the 100 predictions in Supplementary data Table S3 reveals one false negative (O45767, pheromone receptor activity in GO: molecular function). Our sequence analysis shows that O45767 is distant from sequences in the vomeronasal receptor class that putatively function as receptors for pheromones; the shortest distance (*E* value) is 1022, and the average distance is 13 649.

**Figure 6:**
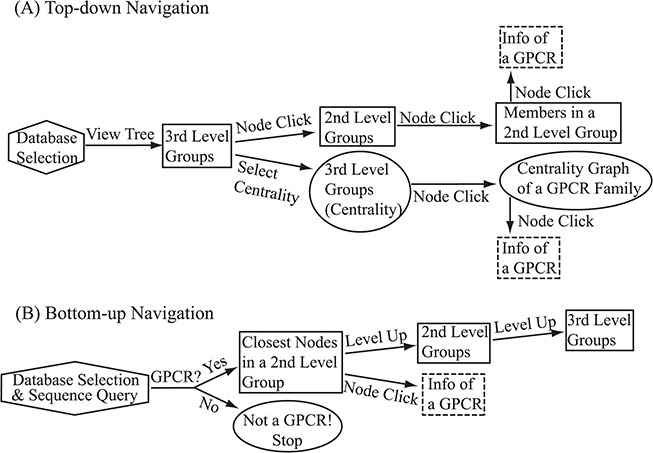
Flowchart of the two navigation interfaces of the SeQuery database, including the top-down navigation scheme (**A**) and the bottom-up navigation scheme (**B**). Source databases are represented by hexagons. In the top-down and bottom-up interface designs, we use solid squares to represent generated MSC graphs and ovals to represent alternative centrality graphs or warnings of detected non-GPCR. Dashed squares represent modal boxes providing information about a GPCR sequence when its corresponding node is clicked.

To detect distant relationships between GPCRs, we applied PSI-BLAST to calculate the smallest distance (*d’*) between the query sequence and sequences in the dataset if *d* > 0.0009 in its initial BLASTp calculation. The smallest BLASTp distance for the sequence O45767 is *d* = 0.002, and its smallest PSI-BLAST distance is *d’* = 2 × 10^−114^ after 10 iterations, with the closest protein being P53452. Thus, the sequence is remotely related to aminergic receptors. For almost all non-GPCR sequences in the test dataset, their smallest PSI-BLAST distance was larger than the threshold distance. Therefore, PSI-BLAST can be used to check if a query sequence is distantly related to GPCRs. The sole exception was sequence Q8LD98, which had *d* = 0.02 and *d’* = 10^−61^ after 10 iterations.

Figure 3 shows the third-level minimum spanning tree diagram of the GPCR superfamily in SeQuery, based on the MSC clustering results. At this resolution level, nodes in the network graph represent receptor families, and edges represent minimum spanning connections between receptor family nodes. The colors and shapes of the nodes are used to distinguish their functions, which are based on GPCRdb annotations and are also labeled on the nodes. The shortest sequence distance between two families is represented by the edge that connects them (the shortest distance is marked on the corresponding edge). The GPCR superfamily in the GPCR2841 dataset is clustered into seven classes, including rhodopsin-like, secretin-like, glutamate, vomeronasal, cAMP, frizzled and T2R receptors. The largest class of GPCRs is the rhodopsin-like receptors, which contains 21 families (503 clusters). Figure 4 shows the second-level minimum spanning tree diagram of the GPCR2841 dataset, in which each node represents a first-level MSC cluster. The classification of GPCRs in SeQuery is generally comparable with the A-F and GRAFS systems, which are two common GPCR classification schemes (33, 34). For instance, among the taste receptors, T1R belongs to class C (glutamate), while T2R is a putative GPCR class. In the A-F classification, T2R is distantly related to class A; in GRAFS, it represents a distinct cluster within the frizzled/taste 2 class. In SeQuery, frizzled receptors and T2R belong to two different sequence classes. Vomeronasal receptors are putatively identified as pheromone receptors and are remotely related to the receptors of the main olfactory system.

The graph-based visualization of the GPCR superfamily can allow users to observe the biological properties of GPCRs. In Figure 5, we show the network graphs of the largest cluster (Pe001) in the peptide receptor family (A) and the largest cluster (Ol001) in the olfactory receptor family (B). The nodes in the graphs represent GPCR sequences, and the edges represent minimum spanning connections between sequences (the shortest distance is marked on the corresponding edge). We found that the conservative core of Pe001 contains 51 similar peptide receptors, for which the pairwise sequence distances are all zero; meanwhile, Ol001 contains only pairs of sequences with non-vanishing distances. These network characteristics suggest that peptide receptors have a much smaller intra-cluster selection pressure than olfactory receptors. On the other hand, Figure 4 shows that peptide receptors are decomposed into three groups and several clusters, while all olfactory receptor clusters form a single group. This suggests that peptide receptors have a larger intra-family (inter-cluster) selection pressure than olfactory receptors. These findings are consistent with our previous calculations using the Nei–Gojobori method of the evolutionary pressures placed on GPCRs (3).

**Figure 7:**
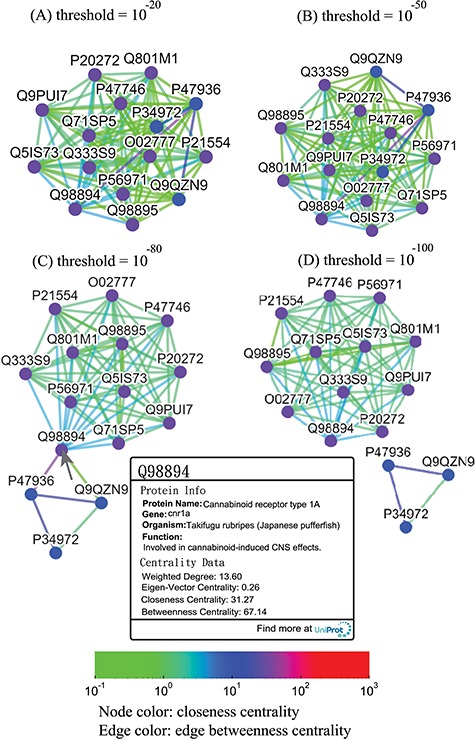
Thresholded sequence similarity network graphs of the cannabinoid receptor family with threshold distances of 10^−20^ (A), 10^−50^ (B), 10^−80^ (C) and 10^−100^ (D). Nodes and edges are colored by the values of their closeness and betweenness centralities, respectively, according to the legend. Upon clicking on a node such as Q98894, the user is presented with a modal box showing both the protein information and the centrality data of the node.

We note that our automatic sequence-based clustering of GPCRs could be helpful in elucidating relationships between the sequence, structure, function and evolution of GPCR sequences, particularly orphan GPCRs for which natural ligands are currently unknown. For example, the MiR002 cluster contains Q923Y7, Q5QD15 and Q5QNP2. In GPCRdb, the first two sequences were annotated as aminergic receptors (trace amine-associated receptor 4), while Q5QNP2 was annotated as a class A orphan/other. The *E*-value between the first two sequences is negligibly small but is 10^−99^ between Q5QD15 and Q5QNP2. It is reasonable to infer that Q5QNP2 is a different type of trace amine-associated receptor. Indeed, Q5QNP2 is annotated as a trace amine-associated receptor 13c in the most recent version of UniProt. So far, only Q5QD15 has been found to have a ligand phenethylamine in GLASS (35); thus, phenethylamine-type compounds are probable ligands of Q923Y7 or Q5QNP2.

## The utility of the database

SeQuery provides two navigation interfaces for exploring the GPCR superfamily, as illustrated in Figure 6. We recommend users to use the Google Chrome browser for optimal viewing quality. At the SeQuery homepage (http://cluster.phy.ntnu.edu.tw), users can select the GPCR2841 dataset and explore the GPCR superfamily from the top down. Alternatively, users can also submit a query sequence and explore its role in the GPCR superfamily from the bottom up.

**Figure 8:**
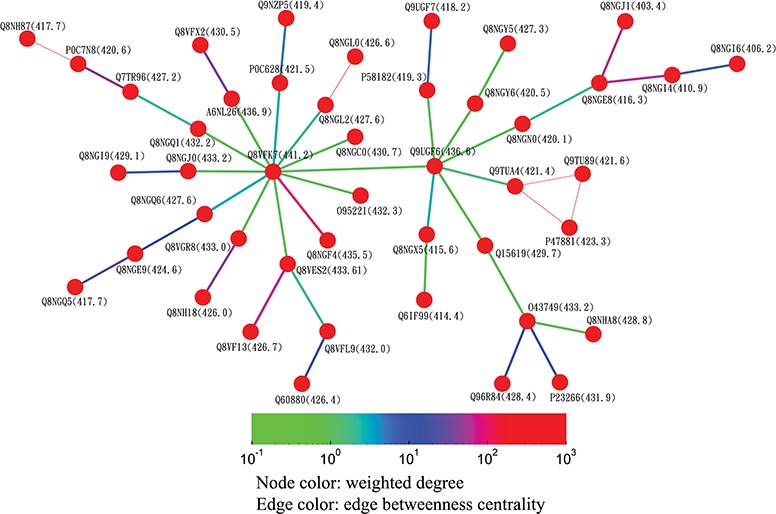
Partial minimum spanning tree of olfactory receptors near the hub sequence Q8VFK7. Nodes are colored based on their closeness centrality values, which are also labeled in parentheses. Edges are colored based on their betweenness centrality values. Thin edges in the graph represent zero-distance sequence pairs.

In the top-down navigation interface, SeQuery first displays a panoramic view of the GPCR superfamily at the third level of clustering, as shown in Figure 3. The same scheme of colors and shapes shown in the legend is used to denote the functions of GPCRs at all three levels of resolution in SeQuery. Each node in the graph represents a GPCR family. When users click a node, SeQuery shows the second-level clustering of the network centered at the clusters that comprise the selected GPCR family. At the second level of clustering, the GPCR superfamily is represented by a minimum spanning tree of receptor clusters as predicted in the first-level MSC clustering. Users can explore the second-level graphs by dragging and zooming the graph or by locating a GPCR family of interest by clicking it in the legend on the left-hand side. When a user clicks on a node in the second-level graph, SeQuery shows the constituent sequences of the selected cluster in a first-level graph. Each node in a first-level graph is a GPCR sequence, and the detailed information for each sequence can be viewed in a modal box by clicking the corresponding node. At the first level of clustering, receptor clusters are represented by a network graph that may or may not contain a core of zero-distance sequences, as shown in Figure 5(A) and (B). If a sequence has a known protein structure, such as P51681 in Figure 5(A), its structure will be displayed on the node.

Alternatively, users can select the ‘Centrality’ button to access the centrality networks in the third-level graph. When users click on a node in the third-level graph, SeQuery displays a centrality graph (with a threshold centrality value of 10^−100^) showing the statistical properties of the corresponding receptor family. Centrality graphs for other threshold values and the MST centrality graph are available by using the slider to adjust the threshold and by clicking the ‘MST Graph’ button, respectively. As an example, in Figure 7, we show the thresholded centrality graphs of the cannabinoid receptor family in the rhodopsin-like class. Nodes are colored according to their closeness centralities, and edges are colored according to their betweenness centralities. The threshold sequence distance values for the subgraphs of Figure 7 are 10^−20^ (A), 10^−50^ (B), 10^−80^ (C) and 10^−100^ (D); edges longer than the threshold are not shown in each subgraph. From Figure 7, it is clear that the edge connecting Q98894 and P47936 has the largest betweenness centrality, and thus the largest potential to disconnect a sequence similarity network if it is removed. Therefore, these two sequences could play a key role in the evolution of cannabinoid receptors. As the cannabinoid receptor family has a large core of zero-distance sequences (Q98894 and other nodes colored in purple), these sequences have the same closeness and eigenvector centralities. However, Q98894 has the largest weighted degree and betweenness centralities, suggesting that this sequence is more central to the family. To further demonstrate the utility of centrality measures, we display a partial minimum spanning tree of the olfactory receptor family in Figure 8, which shows the neighborhood of the most connected hub (Q8VFK7) in the family. Nodes are colored and labeled according to the values of their weighted degree centralities (sequence IDs are also labeled), and edges are colored according to the values of their betweenness centralities. Evidently, the hub has the largest value of centrality measures among its immediate neighborhood (see also the closeness and eigenvector centralities in Supplementary data Figures S1 and S2). These results suggest that hub sequences are more representative, influential and connected in a GPCR family. Figures 7 and 8 can only be reproduced by starting from the ‘Centrality’ tab.

**Figure 9:**
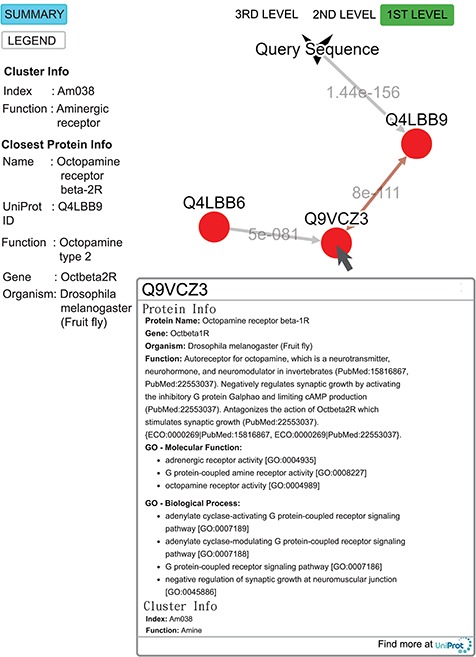
Query result for the submitted sequence G3M4F8 in SeQuery, showing the first-level cluster graph. Information about both the cluster and the closest proteins are shown on the left-hand side. Upon clicking on a node such as Q9VCZ3, the user is presented with a modal box showing both the protein information and the cluster information of the node. A double-headed arrow is used to represent the shortest edge connecting two nodes in the core, and a single-headed arrow (directed toward the node closer to the core) is used to represent all other edges connecting two nodes in the cluster. The edge connecting the query sequence to its closest adjacent sequence is also represented by a single-headed arrow. Each edge is labeled with its length.

In the bottom-up navigation interface, the base dataset GPCR2841 is selected by default, and the user uploads or enters the query sequence at the homepage in the FASTA format. SeQuery determines if the sequence is a GPCR based on its BLASTp distance to sequences in the base dataset. If so (*d* < 0.0009), as shown in Figure 9, SeQuery will display the graph consisting of connections between the query sequence and its neighbors at the first level of clustering, alongside information related to the overall cluster and the closest neighbor of the query sequence. The role of the cluster in the GPCR superfamily can be further investigated at the second or third levels of clustering. If not (*d* > 0.0009), SeQuery will run PSI-BLAST for 10 iterations to calculate the smallest distance (*d’*) between the query sequence and sequences in the GPCR2841 dataset and verify if any remote relationships exist.

To demonstrate the functionality of SeQuery, we submitted a query sequence (UniProt ID: G3M4F8, the sample data for SeQuery at the homepage) in the bottom-up navigation interface. SeQuery determines that it is closest to Q4LBB9 with a distance of 1.4 × 10^−156^ and should belong to cluster Am038 in the aminergic receptor family. As shown in Figure 9, SeQuery shows the network graph consisting of G3M4F8 and cluster Am038, as well as information about the cluster and the protein most closely related to G3M4F8. When each node in the graph is clicked, information about the corresponding sequence is shown in a modal box. UniProt describes Q4LBB9 as the octopamine receptor beta-2R from *Drosophila melanogaster*; it acts as a neurotransmitter, neurohormone and neuromodulator. In the cluster Am038, Q4LBB9 has a greater distance of 8 × 10^−111^ to Q9VCZ3, which is the octopamine receptor beta-1R from *D. melanogaster*. Based on this sequence information alone, the query sequence is predicted to be an ortholog of Q4LBB9, which is consistent with its UniProt annotation as the octopamine receptor beta-2R from *Chilo suppressalis*. Further analysis of G3M4F8 and Q4LBB9 using sequence alignment shows that they have 42.2% sequence identity, as shown in Supplementary data Figure S3. The two sequences also have very similar GO annotations; 9 out of 20 GO terms are common to both sequences, as shown in Supplementary data Table S4.

As an interesting exercise, we entered the recently identified sequence of heliorhodopsins (UniProt ID: A0A2R4S913) in SeQuery (36). SeQuery determined that the heliorhodopsin sequence is not a GPCR since its distance to the closest GPCR sequence (P46090, a peptide receptor) is 0.4. Indeed, heliorhodopsins are a subclass of microbial rhodopsins, the sequences of which share no clearly detectable identity with animal rhodopsins (the GPCR rhodopsin family). The closest sequence in the GPCR rhodopsin family to A0A2R4S913 is P2868, a blue-sensitive opsin, and the sequence distance is 150. However, in the rhodopsin family, 73% of intra-cluster sequence pairs have zero distance, and the median intra-family sequence-pair distance is 7.7 × 10^−63^. Therefore, at the sequence level, we found no evidence that heliorhodopsins are related to the GPCR rhodopsin family.

We also evaluated GPCR isoforms using SeQuery. As shown in Supplementary data Table S5, we considered various isoforms of two human corticotropin-releasing hormone receptor (CRHR) sequences, Q13324 (CRHR2) and P34998 (CRHR1). Among these sequences, Q13324-1 and P34998-1 have been chosen as the canonical sequences of the human CRHR2 and CRHR1. It was found that all six human CRHR2 isoforms are most similar to their canonical sequence, and four human CRHR1 isoforms have almost zero distance to Q76LL8 (CRHR1 sequence of *Macaca mulatta*) or P34998. Among the potential computationally mapped isoforms of human CRHR1, three are most similar to P34998 (human), three are most similar to Q76LL8 (*M. mulatta*), one is most similar to P35353 (*Rattus norvegicus*) and one is most similar to O62772 (*Ovis aries*). We note that all CRHR orthologs in the base dataset are highly similar to each other and form a core in the first level MSC cluster Co001, suggesting that the CRHR family is highly conserved.

## Conclusions and outlook

GPCRs recognize an exceptional variety of extracellular stimuli and consequently serve as essential transporters in eukaryotic signal transduction. Understanding the GPCR superfamily is valuable to theoretical research on cell signaling and molecular recognition, as well as to applied research in drug discovery and disease treatment. We have developed a web-based graph database, SeQuery, which provides an interactive tool for identifying GPCR sequences and visualizing the GPCR superfamily at various characteristic resolutions. By integrating and analyzing GPCR data from three existing databases with our software module, SeQuery is able to respond to queries rapidly and to provide an interactive visual interface for users to understand the properties of GPCR sequences through bottom-up-to-top-down navigation. Our tool is readily extensible to other biological networks, and we aim to expand SeQuery by integrating additional biological databases in future work.

## Author contributions

G.M.H. carried out the study of the G protein-coupled receptor (GPCR) superfamily and prepared the GPCR data. M.K.S. constructed the graph database. C.M.C. conceived of the study and wrote the main manuscript text. All authors reviewed the manuscript.

## Supplementary Material

supp2_baz073

## Notes

**Database URL**: http://cluster.phy.ntnu.edu.tw
