# Supplementary material for: SeQuery: an interactive graph database for visualizing the GPCR superfamily
Source: Database (Oxford). 2019 Jun 25;2019:baz073. doi: 10.1093/database/baz073 (PMC6591535; doi:10.1093/database/baz073)
Supplement: supp2_baz073 [file supp2_baz073.docx]

Supporting Information

For **“SeQuery: An Interactive Graph Database for Visualizing the GPCR Superfamily”**

by Geng-Ming Hu, M.K. Secario, and Chi-Ming Chen

Table of Contents

1. Supplementary Figures S1-S3 discussed in the paper (page 1-3).
2. Supplementary Tables S1-S4 (pages 4-77).


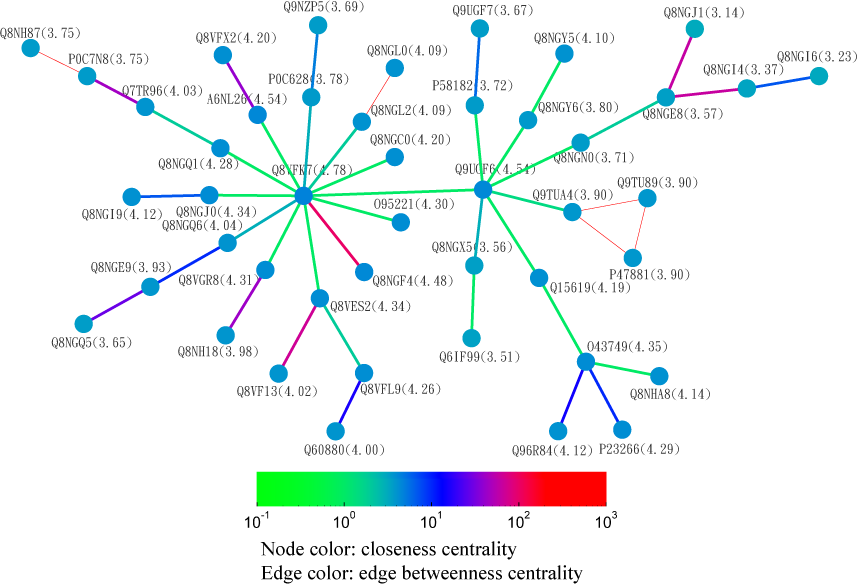


Figure S1. A partial minimum spanning tree of Olfactory Receptors near the hub sequence Q8VFK7. Nodes are colored based on their closeness centrality values which are labeled in the parentheses. Edges are colored based on their betweenness centrality values. Thin edges represent sequence pairs of zero distance.


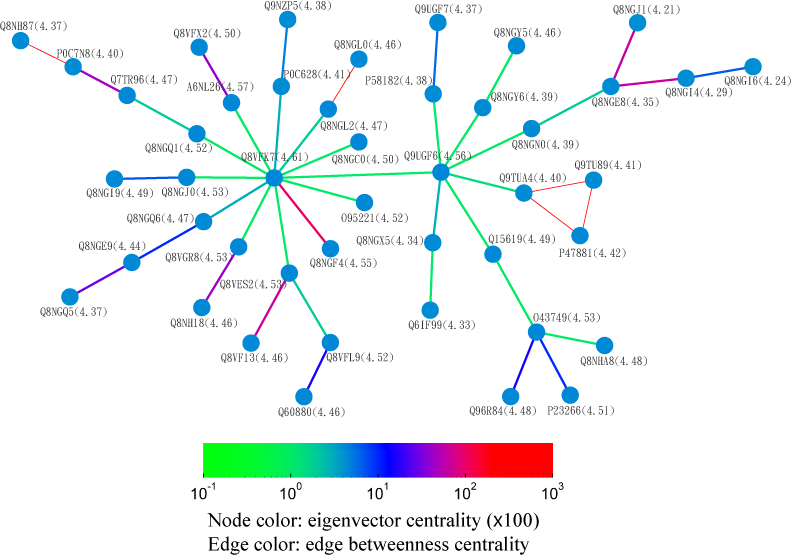


Figure S2. A partial minimum spanning tree of Olfactory Receptors near the hub sequence Q8VFK7. Nodes are colored based on their eigenvector centrality values. The values in the parentheses are 100 times the eigenvector centrality values. Edges are colored based on their betweenness centrality values. Thin edges represent sequence pairs of zero distance.

Figure S3. Sequence alignment of Q4LBB9 and G3M4F8. The asterisk symbol indicates a match between the two sequences.

Table S1. MSC clustering of 2841 GPCR sequences in the base dataset, showing the MSC cluster label, UniProt ID, and level 2 and function labels in GPCRdb of each sequence.

| **MSC cluster label** | **UniProt ID** | **GPCR level 2 label** | **GPCR function label** |
| --- | --- | --- | --- |
| Am001 | P30545 | Aminergic receptor | Alpha Adrenoceptors type 2b |
| Am001 | Q60475 | Aminergic receptor | Alpha Adrenoceptors type 2b |
| Am001 | P18089 | Aminergic receptor | Alpha Adrenoceptors type 2b |
| Am001 | O19012 | Aminergic receptor | Alpha Adrenoceptors type 2b |
| Am001 | O77830 | Aminergic receptor | Alpha Adrenoceptors type 2b |
| Am001 | O77715 | Aminergic receptor | Alpha Adrenoceptors type 2b |
| Am001 | O18935 | Aminergic receptor | Alpha Adrenoceptors type 2b |
| Am001 | P19328 | Aminergic receptor | Alpha Adrenoceptors type 2b |
| Am001 | O77721 | Aminergic receptor | Alpha Adrenoceptors type 2b |
| Am001 | O19014 | Aminergic receptor | Alpha Adrenoceptors type 2b |
| Am001 | O19054 | Aminergic receptor | Alpha Adrenoceptors type 2b |
| Am001 | O19091 | Aminergic receptor | Alpha Adrenoceptors type 2b |
| Am001 | O77700 | Aminergic receptor | Alpha Adrenoceptors type 2b |
| Am001 | O77713 | Aminergic receptor | Alpha Adrenoceptors type 2b |
| Am002 | P35404 | Aminergic receptor | Serotonin type 1b |
| Am002 | P28222 | Aminergic receptor | Serotonin type 1b |
| Am002 | O08892 | Aminergic receptor | Serotonin type 1b |
| Am002 | P60020 | Aminergic receptor | Serotonin type 1b |
| Am002 | Q0EAB5 | Aminergic receptor | Serotonin type 1b |
| Am002 | P46636 | Aminergic receptor | Serotonin type 1b |
| Am002 | Q588Y6 | Aminergic receptor | Serotonin type 1b |
| Am002 | P49144 | Aminergic receptor | Serotonin type 1b |
| Am002 | P56496 | Aminergic receptor | Serotonin type 1b |
| Am002 | P28334 | Aminergic receptor | Serotonin type 1b |
| Am002 | P79250 | Aminergic receptor | Serotonin type 1b |
| Am002 | P28564 | Aminergic receptor | Serotonin type 1b |
| Am002 | Q6XXX8 | Aminergic receptor | Serotonin type 1b |
| Am003 | Q6XXY0 | Aminergic receptor | Serotonin type 1a |
| Am003 | Q9N298 | Aminergic receptor | Serotonin type 1a |
| Am003 | Q98998 | Aminergic receptor | Serotonin type 1a |
| Am003 | P19327 | Aminergic receptor | Serotonin type 1a |
| Am003 | Q9N297 | Aminergic receptor | Serotonin type 1a |
| Am003 | Q64264 | Aminergic receptor | Serotonin type 1a |
| Am003 | O42384 | Aminergic receptor | Serotonin type 1a |
| Am003 | O42385 | Aminergic receptor | Serotonin type 1a |
| Am003 | Q9N296 | Aminergic receptor | Serotonin type 1a |
| Am003 | Q0EAB6 | Aminergic receptor | Serotonin type 1a |
| Am003 | Q6XXX9 | Aminergic receptor | Serotonin type 1a |
| Am003 | P08908 | Aminergic receptor | Serotonin type 1a |
| Am004 | Q5QD11 | Aminergic receptor | Trace amine type 7 |
| Am004 | Q5QD09 | Aminergic receptor | Trace amine type 7 |
| Am004 | Q923X5 | Aminergic receptor | Trace amine type 7 |
| Am004 | Q923Y2 | Aminergic receptor | Trace amine type 15 |
| Am004 | Q923X6 | Aminergic receptor | Trace amine type 7 |
| Am004 | Q923Y4 | Aminergic receptor | Trace amine type 7 |
| Am004 | Q5QD12 | Aminergic receptor | Trace amine type 15 |
| Am004 | Q923Y1 | Aminergic receptor | Trace amine type 7 |
| Am004 | Q5QD21 | Aminergic receptor | Trace amine type 7 |
| Am004 | Q5QD10 | Aminergic receptor | Trace amine type 7 |
| Am004 | Q5QD08 | Aminergic receptor | Trace amine type 7 |
| Am004 | Q923X8 | Aminergic receptor | Trace amine type 7 |
| Am005 | Q4KWL2 | Aminergic receptor | Beta Adrenoceptors type 2 |
| Am005 | P54833 | Aminergic receptor | Beta Adrenoceptors type 2 |
| Am005 | Q28509 | Aminergic receptor | Beta Adrenoceptors type 2 |
| Am005 | P07550 | Aminergic receptor | Beta Adrenoceptors type 2 |
| Am005 | Q8K4Z4 | Aminergic receptor | Beta Adrenoceptors type 2 |
| Am005 | Q28997 | Aminergic receptor | Beta Adrenoceptors type 2 |
| Am005 | P10608 | Aminergic receptor | Beta Adrenoceptors type 2 |
| Am005 | Q8UUY8 | Aminergic receptor | Beta Adrenoceptors type 2 |
| Am005 | Q9TST5 | Aminergic receptor | Beta Adrenoceptors type 2 |
| Am005 | P18762 | Aminergic receptor | Beta Adrenoceptors type 2 |
| Am005 | Q28044 | Aminergic receptor | Beta Adrenoceptors type 2 |
| Am005 | P04274 | Aminergic receptor | Beta Adrenoceptors type 2 |
| Am006 | Q6TLI9 | Aminergic receptor | Dopamine Vertebrate type 2 |
| Am006 | Q9GJU1 | Aminergic receptor | Dopamine Vertebrate type 2 |
| Am006 | P61168 | Aminergic receptor | Dopamine Vertebrate type 2 |
| Am006 | P34973 | Aminergic receptor | Dopamine Vertebrate type 2 |
| Am006 | O73810 | Aminergic receptor | Dopamine Vertebrate type 2 |
| Am006 | P60026 | Aminergic receptor | Dopamine Vertebrate type 2 |
| Am006 | P52702 | Aminergic receptor | Dopamine Vertebrate type 2 |
| Am006 | P24628 | Aminergic receptor | Dopamine Vertebrate type 2 |
| Am006 | P61169 | Aminergic receptor | Dopamine Vertebrate type 2 |
| Am006 | P20288 | Aminergic receptor | Dopamine Vertebrate type 2 |
| Am006 | P53453 | Aminergic receptor | Dopamine Vertebrate type 2 |
| Am006 | P14416 | Aminergic receptor | Dopamine Vertebrate type 2 |
| Am007 | Q9TST4 | Aminergic receptor | Beta Adrenoceptors type 3 |
| Am007 | Q28524 | Aminergic receptor | Beta Adrenoceptors type 3 |
| Am007 | P46626 | Aminergic receptor | Beta Adrenoceptors type 3 |
| Am007 | Q60483 | Aminergic receptor | Beta Adrenoceptors type 3 |
| Am007 | P13945 | Aminergic receptor | Beta Adrenoceptors type 3 |
| Am007 | Q95252 | Aminergic receptor | Beta Adrenoceptors type 3 |
| Am007 | O02662 | Aminergic receptor | Beta Adrenoceptors type 3 |
| Am007 | Q9XT58 | Aminergic receptor | Beta Adrenoceptors type 3 |
| Am007 | Q9XT57 | Aminergic receptor | Beta Adrenoceptors type 3 |
| Am007 | P25962 | Aminergic receptor | Beta Adrenoceptors type 3 |
| Am007 | P26255 | Aminergic receptor | Beta Adrenoceptors type 3 |
| Am008 | Q923Y5 | Aminergic receptor | Trace amine type 6 |
| Am008 | Q969N4 | Aminergic receptor | Trace amine type 17 |
| Am008 | Q5QD13 | Aminergic receptor | Trace amine type 6 |
| Am008 | Q923X9 | Aminergic receptor | Trace amine type 8 |
| Am008 | Q923Y3 | Aminergic receptor | Trace amine type 8 |
| Am008 | Q5QD06 | Aminergic receptor | Trace amine type 8 |
| Am008 | Q5W8W0 | Aminergic receptor | Trace amine type 6 |
| Am008 | Q96RI8 | Aminergic receptor | Trace amine type 6 |
| Am008 | Q923Y0 | Aminergic receptor | Trace amine type 8 |
| Am008 | Q5QD05 | Aminergic receptor | Trace amine type 8 |
| Am008 | Q5QD07 | Aminergic receptor | Trace amine type 8 |
| Am009 | P08173 | Aminergic receptor | Musc. acetylcholine Vertebrate type 4 |
| Am009 | Q9ERZ4 | Aminergic receptor | Musc. acetylcholine Vertebrate type 2 |
| Am009 | P10980 | Aminergic receptor | Musc. acetylcholine Vertebrate type 2 |
| Am009 | P08172 | Aminergic receptor | Musc. acetylcholine Vertebrate type 2 |
| Am009 | Q9N2A7 | Aminergic receptor | Musc. acetylcholine Vertebrate type 2 |
| Am009 | P32211 | Aminergic receptor | Musc. acetylcholine Vertebrate type 4 |
| Am009 | P41985 | Aminergic receptor | Musc. acetylcholine Vertebrate type 2 |
| Am009 | P06199 | Aminergic receptor | Musc. acetylcholine Vertebrate type 2 |
| Am009 | P30372 | Aminergic receptor | Musc. acetylcholine Vertebrate type 2 |
| Am009 | P17200 | Aminergic receptor | Musc. acetylcholine Vertebrate type 4 |
| Am010 | P42289 | Aminergic receptor | Dopamine Vertebrate type 1 |
| Am010 | Q95136 | Aminergic receptor | Dopamine Vertebrate type 1 |
| Am010 | P21728 | Aminergic receptor | Dopamine Vertebrate type 1 |
| Am010 | Q61616 | Aminergic receptor | Dopamine Vertebrate type 1 |
| Am010 | P50130 | Aminergic receptor | Dopamine Vertebrate type 1 |
| Am010 | P35406 | Aminergic receptor | Dopamine Vertebrate type 1 |
| Am010 | P42288 | Aminergic receptor | Dopamine Vertebrate type 1 |
| Am010 | O77680 | Aminergic receptor | Dopamine Vertebrate type 1 |
| Am010 | P53452 | Aminergic receptor | Dopamine Vertebrate type 1 |
| Am010 | P18901 | Aminergic receptor | Dopamine Vertebrate type 1 |
| Am011 | P14842 | Aminergic receptor | Serotonin type 2a |
| Am011 | Q75Z89 | Aminergic receptor | Serotonin type 2a |
| Am011 | P18599 | Aminergic receptor | Serotonin type 2a |
| Am011 | Q5R4Q6 | Aminergic receptor | Serotonin type 2a |
| Am011 | P28223 | Aminergic receptor | Serotonin type 2a |
| Am011 | P35363 | Aminergic receptor | Serotonin type 2a |
| Am011 | P50129 | Aminergic receptor | Serotonin type 2a |
| Am011 | P50128 | Aminergic receptor | Serotonin type 2a |
| Am011 | O46635 | Aminergic receptor | Serotonin type 2a |
| Am012 | Q9N2A3 | Aminergic receptor | Musc. acetylcholine Vertebrate type 3 |
| Am012 | P20309 | Aminergic receptor | Musc. acetylcholine Vertebrate type 3 |
| Am012 | P49578 | Aminergic receptor | Musc. acetylcholine Vertebrate type 3 |
| Am012 | P08483 | Aminergic receptor | Musc. acetylcholine Vertebrate type 3 |
| Am012 | Q9U7D5 | Aminergic receptor | Musc. acetylcholine Non Vertebrate |
| Am012 | P11483 | Aminergic receptor | Musc. acetylcholine Vertebrate type 3 |
| Am012 | Q9N2A2 | Aminergic receptor | Musc. acetylcholine Vertebrate type 3 |
| Am012 | P41984 | Aminergic receptor | Musc. acetylcholine Vertebrate type 3 |
| Am012 | Q9ERZ3 | Aminergic receptor | Musc. acetylcholine Vertebrate type 3 |
| Am013 | Q28998 | Aminergic receptor | Beta Adrenoceptors type 1 |
| Am013 | Q9TST6 | Aminergic receptor | Beta Adrenoceptors type 1 |
| Am013 | P08588 | Aminergic receptor | Beta Adrenoceptors type 1 |
| Am013 | P79148 | Aminergic receptor | Beta Adrenoceptors type 1 |
| Am013 | P43141 | Aminergic receptor | Beta Adrenoceptors type 4 |
| Am013 | P47899 | Aminergic receptor | Beta Adrenoceptors type 1 |
| Am013 | P18090 | Aminergic receptor | Beta Adrenoceptors type 1 |
| Am013 | P34971 | Aminergic receptor | Beta Adrenoceptors type 1 |
| Am013 | Q9TT96 | Aminergic receptor | Beta Adrenoceptors type 1 |
| Am014 | Q90WY5 | Aminergic receptor | Alpha Adrenoceptors type 2b |
| Am014 | Q90WY4 | Aminergic receptor | Alpha Adrenoceptors type 2a |
| Am014 | Q60474 | Aminergic receptor | Alpha Adrenoceptors type 2a |
| Am014 | P18871 | Aminergic receptor | Alpha Adrenoceptors type 2a |
| Am014 | Q01338 | Aminergic receptor | Alpha Adrenoceptors type 2a |
| Am014 | P22909 | Aminergic receptor | Alpha Adrenoceptors type 2a |
| Am014 | Q28838 | Aminergic receptor | Alpha Adrenoceptors type 2a |
| Am014 | P08913 | Aminergic receptor | Alpha Adrenoceptors type 2a |
| Am015 | P79748 | Aminergic receptor | Serotonin type 1d |
| Am015 | P49145 | Aminergic receptor | Serotonin type 1d |
| Am015 | P28565 | Aminergic receptor | Serotonin type 1d |
| Am015 | P28221 | Aminergic receptor | Serotonin type 1d |
| Am015 | P79400 | Aminergic receptor | Serotonin type 1d |
| Am015 | Q61224 | Aminergic receptor | Serotonin type 1d |
| Am015 | P11614 | Aminergic receptor | Serotonin type 1d |
| Am015 | Q60484 | Aminergic receptor | Serotonin type 1d |
| Am016 | Q76MS7 | Aminergic receptor | Histamine type 2 |
| Am016 | P25021 | Aminergic receptor | Histamine type 2 |
| Am016 | P61752 | Aminergic receptor | Histamine type 2 |
| Am016 | P17124 | Aminergic receptor | Histamine type 2 |
| Am016 | P60021 | Aminergic receptor | Histamine type 2 |
| Am016 | P25102 | Aminergic receptor | Histamine type 2 |
| Am016 | P97292 | Aminergic receptor | Histamine type 2 |
| Am016 | P47747 | Aminergic receptor | Histamine type 2 |
| Am017 | P31389 | Aminergic receptor | Histamine type 1 |
| Am017 | Q9N2B0 | Aminergic receptor | Histamine type 1 |
| Am017 | P31390 | Aminergic receptor | Histamine type 1 |
| Am017 | P70174 | Aminergic receptor | Histamine type 1 |
| Am017 | Q9N2B1 | Aminergic receptor | Histamine type 1 |
| Am017 | P30546 | Aminergic receptor | Histamine type 1 |
| Am017 | P35367 | Aminergic receptor | Histamine type 1 |
| Am018 | P43140 | Aminergic receptor | Alpha Adrenoceptors type 1a |
| Am018 | Q9WU25 | Aminergic receptor | Alpha Adrenoceptors type 1a |
| Am018 | Q91175 | Aminergic receptor | Alpha Adrenoceptors type 1a |
| Am018 | P97718 | Aminergic receptor | Alpha Adrenoceptors type 1a |
| Am018 | O02824 | Aminergic receptor | Alpha Adrenoceptors type 1a |
| Am018 | P18130 | Aminergic receptor | Alpha Adrenoceptors type 1a |
| Am018 | P35348 | Aminergic receptor | Alpha Adrenoceptors type 1a |
| Am019 | P12657 | Aminergic receptor | Musc. acetylcholine Vertebrate type 1 |
| Am019 | P11229 | Aminergic receptor | Musc. acetylcholine Vertebrate type 1 |
| Am019 | Q5R949 | Aminergic receptor | Musc. acetylcholine Vertebrate type 1 |
| Am019 | P08482 | Aminergic receptor | Musc. acetylcholine Vertebrate type 1 |
| Am019 | P56489 | Aminergic receptor | Musc. acetylcholine Vertebrate type 1 |
| Am019 | P04761 | Aminergic receptor | Musc. acetylcholine Vertebrate type 1 |
| Am020 | Q5IS53 | Aminergic receptor | Musc. acetylcholine Vertebrate type 5 |
| Am020 | Q5IS98 | Aminergic receptor | Musc. acetylcholine Vertebrate type 5 |
| Am020 | Q920H4 | Aminergic receptor | Musc. acetylcholine Vertebrate type 5 |
| Am020 | P56490 | Aminergic receptor | Musc. acetylcholine Vertebrate type 5 |
| Am020 | P08911 | Aminergic receptor | Musc. acetylcholine Vertebrate type 5 |
| Am020 | P08912 | Aminergic receptor | Musc. acetylcholine Vertebrate type 5 |
| Am021 | P52703 | Aminergic receptor | Dopamine Vertebrate type 3 |
| Am021 | Q5IS72 | Aminergic receptor | Dopamine Vertebrate type 3 |
| Am021 | P35462 | Aminergic receptor | Dopamine Vertebrate type 3 |
| Am021 | P30728 | Aminergic receptor | Dopamine Vertebrate type 3 |
| Am021 | P19020 | Aminergic receptor | Dopamine Vertebrate type 3 |
| Am022 | P32305 | Aminergic receptor | Serotonin type 7 |
| Am022 | P50407 | Aminergic receptor | Serotonin type 7 |
| Am022 | P34969 | Aminergic receptor | Serotonin type 7 |
| Am022 | P32304 | Aminergic receptor | Serotonin type 7 |
| Am022 | Q91559 | Aminergic receptor | Serotonin type 7 |
| Am023 | P35405 | Aminergic receptor | Alpha Adrenoceptors type 2c |
| Am023 | Q01337 | Aminergic receptor | Alpha Adrenoceptors type 2c |
| Am023 | P18825 | Aminergic receptor | Alpha Adrenoceptors type 2c |
| Am023 | Q60476 | Aminergic receptor | Alpha Adrenoceptors type 2c |
| Am023 | P22086 | Aminergic receptor | Alpha Adrenoceptors type 2c |
| Am024 | Q9TTM9 | Aminergic receptor | Alpha Adrenoceptors type 1d |
| Am024 | P25100 | Aminergic receptor | Alpha Adrenoceptors type 1d |
| Am024 | P23944 | Aminergic receptor | Alpha Adrenoceptors type 1d |
| Am024 | P97714 | Aminergic receptor | Alpha Adrenoceptors type 1d |
| Am024 | O02666 | Aminergic receptor | Alpha Adrenoceptors type 1d |
| Am025 | P97717 | Aminergic receptor | Alpha Adrenoceptors type 1b |
| Am025 | P18841 | Aminergic receptor | Alpha Adrenoceptors type 1b |
| Am025 | P15823 | Aminergic receptor | Alpha Adrenoceptors type 1b |
| Am025 | P35368 | Aminergic receptor | Alpha Adrenoceptors type 1b |
| Am025 | P11615 | Aminergic receptor | Alpha Adrenoceptors type 1b |
| Am026 | P28335 | Aminergic receptor | Serotonin type 1c |
| Am026 | P34968 | Aminergic receptor | Serotonin type 1c |
| Am026 | Q60F97 | Aminergic receptor | Serotonin type 1c |
| Am026 | P08909 | Aminergic receptor | Serotonin type 1c |
| Am026 | Q5IS66 | Aminergic receptor | Serotonin type 1c |
| Am027 | P21917 | Aminergic receptor | Dopamine Vertebrate type 4 |
| Am027 | P30729 | Aminergic receptor | Dopamine Vertebrate type 4 |
| Am027 | Q6TLJ0 | Aminergic receptor | Dopamine Vertebrate type 4 |
| Am027 | P51436 | Aminergic receptor | Dopamine Vertebrate type 4 |
| Am028 | Q17232 | Aminergic receptor | Octopamine type 6 |
| Am028 | P22270 | Aminergic receptor | Octopamine type 4 |
| Am028 | Q25188 | Aminergic receptor | Octopamine type 6 |
| Am028 | O02213 | Aminergic receptor | Octopamine type 6 |
| Am029 | P30940 | Aminergic receptor | Serotonin type 1f |
| Am029 | P30939 | Aminergic receptor | Serotonin type 1f |
| Am029 | Q02284 | Aminergic receptor | Serotonin type 1f |
| Am029 | O08890 | Aminergic receptor | Serotonin type 1f |
| Am030 | P50406 | Aminergic receptor | Serotonin type 6 |
| Am030 | P31388 | Aminergic receptor | Serotonin type 6 |
| Am030 | Q5IS65 | Aminergic receptor | Serotonin type 6 |
| Am030 | Q9R1C8 | Aminergic receptor | Serotonin type 6 |
| Am031 | O14804 | Aminergic receptor | Trace amine type 5 |
| Am031 | Q5QD23 | Aminergic receptor | Trace amine type 5 |
| Am031 | Q5QD28 | Aminergic receptor | Trace amine type 5 |
| Am031 | Q5QD14 | Aminergic receptor | Trace amine type 5 |
| Am032 | Q13639 | Aminergic receptor | Serotonin type 4 |
| Am032 | P97288 | Aminergic receptor | Serotonin type 4 |
| Am032 | Q62758 | Aminergic receptor | Serotonin type 4 |
| Am032 | O70528 | Aminergic receptor | Serotonin type 4 |
| Am033 | Q9QXI3 | Aminergic receptor | Trace amine type 9 |
| Am033 | Q8BZA7 | Aminergic receptor | Trace amine type 9 |
| Am033 | Q96P69 | Aminergic receptor | Trace amine type 9 |
| Am033 | Q8NDV2 | Aminergic receptor | Trace amine type 9 |
| Am034 | P25115 | Aminergic receptor | Dopamine Vertebrate type 5 |
| Am034 | Q8BLD9 | Aminergic receptor | Dopamine Vertebrate type 5 |
| Am034 | P42290 | Aminergic receptor | Dopamine Vertebrate type 5 |
| Am034 | P21918 | Aminergic receptor | Dopamine Vertebrate type 5 |
| Am035 | Q02152 | Aminergic receptor | Serotonin type 2b |
| Am035 | Q8UUG8 | Aminergic receptor | Serotonin type 2b |
| Am035 | P30994 | Aminergic receptor | Serotonin type 2b |
| Am035 | P41595 | Aminergic receptor | Serotonin type 2b |
| Am036 | Q9QYN8 | Aminergic receptor | Histamine type 3 |
| Am036 | P58406 | Aminergic receptor | Histamine type 3 |
| Am036 | Q9JI35 | Aminergic receptor | Histamine type 3 |
| Am036 | Q9Y5N1 | Aminergic receptor | Histamine type 3 |
| Am037 | Q25414 | Aminergic receptor | Serotonin Insect |
| Am037 | Q25190 | Aminergic receptor | Serotonin Insect |
| Am037 | Q17239 | Aminergic receptor | Serotonin Insect |
| Am038 | Q4LBB9 | Aminergic receptor | Octopamine type 2 |
| Am038 | Q4LBB6 | Aminergic receptor | Dopamine Non Vertebrate |
| Am038 | Q9VCZ3 | Aminergic receptor | Octopamine type 1 |
| Am039 | Q91ZY2 | Aminergic receptor | Histamine type 4 |
| Am039 | Q91ZY1 | Aminergic receptor | Histamine type 4 |
| Am039 | Q9H3N8 | Aminergic receptor | Histamine type 4 |
| Am040 | Q8IZ08 | Aminergic receptor | Dopamine Vertebrate type 1 |
| Am040 | Q7TQP2 | Aminergic receptor | Dopamine Vertebrate type 1 |
| Am040 | Q7TQN7 | Aminergic receptor | Dopamine Vertebrate type 1 |
| Am041 | Q8TDV5 | Aminergic receptor | Trace amine type 9 |
| Am041 | Q7TQP3 | Aminergic receptor | Trace amine type 9 |
| Am041 | Q7TQN8 | Aminergic receptor | Trace amine type 9 |
| Am042 | Q9P1P5 | Aminergic receptor | Trace amine type 2 |
| Am042 | Q5QD17 | Aminergic receptor | Trace amine type 2 |
| Am042 | Q5QD25 | Aminergic receptor | Trace amine type 2 |
| Am043 | Q5QD16 | Aminergic receptor | Trace amine type 3 |
| Am043 | Q5QD24 | Aminergic receptor | Trace amine type 3 |
| Am043 | Q9P1P4 | Aminergic receptor | Trace amine type 3 |
| Am044 | P35364 | Aminergic receptor | Serotonin type 5a |
| Am044 | P47898 | Aminergic receptor | Serotonin type 5a |
| Am044 | P30966 | Aminergic receptor | Serotonin type 5a |
| Am045 | P47800 | Aminergic receptor | Dopamine Vertebrate type 1 |
| Am045 | P53454 | Aminergic receptor | Dopamine Vertebrate type 1 |
| Am045 | P42291 | Aminergic receptor | Dopamine Vertebrate type 1 |
| Am046 | P30951 | Aminergic receptor | Trace amine type 9 |
| Am046 | P35412 | Aminergic receptor | Trace amine type 9 |
| Am046 | P47775 | Aminergic receptor | Trace amine type 9 |
| Am047 | Q8K1Q3 | Aminergic receptor | Trace amine type 9 |
| Am047 | P46089 | Aminergic receptor | Trace amine type 9 |
| Am047 | P35413 | Aminergic receptor | Trace amine type 9 |
| Am048 | Q6YNI2 | Aminergic receptor | Trace amine type 9 |
| Am048 | P51651 | Aminergic receptor | Trace amine type 9 |
| Am048 | P46095 | Aminergic receptor | Trace amine type 9 |
| Am049 | P32251 | Aminergic receptor | Alpha Adrenoceptors type 2d |
| Am049 | Q8JG70 | Aminergic receptor | Alpha Adrenoceptors type 2d |
| Am049 | Q8JG69 | Aminergic receptor | Alpha Adrenoceptors type 2d |
| Am050 | Q8HZ64 | Aminergic receptor | Trace amine type 1 |
| Am050 | Q5QD29 | Aminergic receptor | Trace amine type 1 |
| Am050 | Q96RJ0 | Aminergic receptor | Trace amine type 1 |
| Am051 | Q6VB83 | Aminergic receptor | Serotonin type 1e |
| Am051 | P28566 | Aminergic receptor | Serotonin type 1e |
| Am051 | Q9N2B6 | Aminergic receptor | Serotonin type 1e |
| Am052 | P28285 | Aminergic receptor | Serotonin Insect |
| Am052 | P28286 | Aminergic receptor | Serotonin Insect |
| Am053 | O42574 | Aminergic receptor | Beta Adrenoceptors type 1 |
| Am053 | P07700 | Aminergic receptor | Beta Adrenoceptors type 1 |
| Am054 | Q91081 | Aminergic receptor | Alpha Adrenoceptors type 2c |
| Am054 | Q90WY6 | Aminergic receptor | Alpha Adrenoceptors type 2c |
| Am055 | O95800 | Aminergic receptor | Trace amine type 9 |
| Am055 | Q6X632 | Aminergic receptor | Trace amine type 9 |
| Am056 | Q923Y9 | Aminergic receptor | Trace amine type 1 |
| Am056 | Q923Y8 | Aminergic receptor | Trace amine type 1 |
| Am057 | Q25322 | Aminergic receptor | Octopamine type 6 |
| Am057 | Q25321 | Aminergic receptor | Octopamine type 6 |
| Am058 | Q16950 | Aminergic receptor | Dopamine Insect type 1 |
| Am058 | Q16951 | Aminergic receptor | Dopamine Insect type 1 |
| Am059 | P31387 | Aminergic receptor | Serotonin type 5b |
| Am059 | P35365 | Aminergic receptor | Serotonin type 5b |
| Am060 | Q61H86 | Aminergic receptor | Dopamine Other |
| Am060 | Q6RYS9 | Aminergic receptor | Dopamine Other |
| Br001 | Q8CGM1 | Brain-specific angiogenesis inhibitor | Brain-specific angiogenesis inhibitor (BAI) type 2 |
| Br001 | Q5R7Y0 | Brain-specific angiogenesis inhibitor | Brain-specific angiogenesis inhibitor (BAI) type 2 |
| Br001 | O60241 | Brain-specific angiogenesis inhibitor | Brain-specific angiogenesis inhibitor (BAI) type 2 |
| Br001 | Q80ZF8 | Brain-specific angiogenesis inhibitor | Brain-specific angiogenesis inhibitor (BAI) type 3 |
| Br001 | O60242 | Brain-specific angiogenesis inhibitor | Brain-specific angiogenesis inhibitor (BAI) type 3 |
| Br001 | O14514 | Brain-specific angiogenesis inhibitor | Brain-specific angiogenesis inhibitor |
| Br001 | Q3UHD1 | Brain-specific angiogenesis inhibitor | Brain-specific angiogenesis inhibitor |
| Ca001 | Q9IB86 | Calcitonin receptor | Calcitonin |
| Ca001 | Q68EK2 | Calcitonin receptor | Calcitonin |
| Ca001 | Q8AXU4 | Calcitonin receptor | Calcitonin |
| Ca001 | Q7ZXS8 | Calcitonin receptor | Calcitonin |
| Ca001 | Q9R1W5 | Calcitonin receptor | Calcitonin |
| Ca001 | Q8WN93 | Calcitonin receptor | Calcitonin |
| Ca001 | Q63118 | Calcitonin receptor | Calcitonin |
| Ca001 | Q16602 | Calcitonin receptor | Calcitonin |
| Ca001 | A6QP74 | Calcitonin receptor | Calcitonin |
| Ca001 | Q0P4Y4 | Calcitonin receptor | Calcitonin |
| cA001 | P13773 | cAMP receptor | cAMP receptors |
| cA001 | P35352 | cAMP receptor | cAMP receptors |
| cA001 | P34907 | cAMP receptor | cAMP receptors |
| cA001 | Q9TX43 | cAMP receptor | cAMP receptors |
| Ca002 | P25117 | Calcitonin receptor | Calcitonin |
| Ca002 | Q60755 | Calcitonin receptor | Calcitonin |
| Ca002 | P30988 | Calcitonin receptor | Calcitonin |
| Ca002 | O08893 | Calcitonin receptor | Calcitonin |
| Ca002 | P79222 | Calcitonin receptor | Calcitonin |
| Ca002 | P32214 | Calcitonin receptor | Calcitonin |
| Cb001 | Q98895 | Cannabinoid receptor | Cannabinoid type 1 |
| Cb001 | Q71SP5 | Cannabinoid receptor | Cannabinoid type 1 |
| Cb001 | P21554 | Cannabinoid receptor | Cannabinoid type 1 |
| Cb001 | O02777 | Cannabinoid receptor | Cannabinoid type 1 |
| Cb001 | P56971 | Cannabinoid receptor | Cannabinoid type 1 |
| Cb001 | Q333S9 | Cannabinoid receptor | Cannabinoid type 1 |
| Cb001 | Q801M1 | Cannabinoid receptor | Cannabinoid type 1 |
| Cb001 | P47746 | Cannabinoid receptor | Cannabinoid type 1 |
| Cb001 | Q5IS73 | Cannabinoid receptor | Cannabinoid type 1 |
| Cb001 | Q9PUI7 | Cannabinoid receptor | Cannabinoid type 1 |
| Cb001 | Q98894 | Cannabinoid receptor | Cannabinoid type 1 |
| Cb001 | P20272 | Cannabinoid receptor | Cannabinoid type 1 |
| Cb002 | Q9QZN9 | Cannabinoid receptor | Cannabinoid type 2 |
| Cb002 | P47936 | Cannabinoid receptor | Cannabinoid type 2 |
| Cb002 | P34972 | Cannabinoid receptor | Cannabinoid type 2 |
| CE001 | Q9R0M0 | Cadherin EGF LAG seven-pass G-type receptor | Cadherin EGF LAG (CELSR) |
| CE001 | Q9NYQ6 | Cadherin EGF LAG seven-pass G-type receptor | Cadherin EGF LAG (CELSR) |
| CE001 | Q9QYP2 | Cadherin EGF LAG seven-pass G-type receptor | Cadherin EGF LAG (CELSR) |
| CE001 | Q91ZI0 | Cadherin EGF LAG seven-pass G-type receptor | Cadherin EGF LAG (CELSR) |
| CE001 | O88278 | Cadherin EGF LAG seven-pass G-type receptor | Cadherin EGF LAG (CELSR) |
| CE001 | Q9NYQ7 | Cadherin EGF LAG seven-pass G-type receptor | Cadherin EGF LAG (CELSR) |
| CE001 | Q9V5N8 | Cadherin EGF LAG seven-pass G-type receptor | Cadherin EGF LAG (CELSR) |
| CE001 | Q9HCU4 | Cadherin EGF LAG seven-pass G-type receptor | Cadherin EGF LAG (CELSR) |
| CE001 | O35161 | Cadherin EGF LAG seven-pass G-type receptor | Cadherin EGF LAG (CELSR) |
| Co001 | P34998 | Corticotropin-releasing factor receptor | Corticotropin-releasing factor receptor |
| Co001 | O42602 | Corticotropin-releasing factor receptor | Corticotropin-releasing factor receptor |
| Co001 | Q60748 | Corticotropin-releasing factor receptor | Corticotropin-releasing factor receptor |
| Co001 | Q13324 | Corticotropin-releasing factor receptor | Corticotropin-releasing factor receptor |
| Co001 | P47866 | Corticotropin-releasing factor receptor | Corticotropin-releasing factor receptor |
| Co001 | O42603 | Corticotropin-releasing factor receptor | Corticotropin-releasing factor receptor |
| Co001 | O62772 | Corticotropin-releasing factor receptor | Corticotropin-releasing factor receptor |
| Co001 | Q90812 | Corticotropin-releasing factor receptor | Corticotropin-releasing factor receptor |
| Co001 | P35347 | Corticotropin-releasing factor receptor | Corticotropin-releasing factor receptor |
| Co001 | P35353 | Corticotropin-releasing factor receptor | Corticotropin-releasing factor receptor |
| Co001 | Q76LL8 | Corticotropin-releasing factor receptor | Corticotropin-releasing factor receptor |
| Di001 | Q16983 | Diuretic hormone receptor | Diuretic hormone |
| Di001 | P35464 | Diuretic hormone receptor | Diuretic hormone |
| EG001 | Q61549 | EGF-like module receptor 1 | ERM1 |
| EG001 | Q5Y4N8 | EGF-like module receptor 1 | ERM1 |
| EG001 | Q14246 | EGF-like module receptor 1 | ERM1 |
| Fr001 | Q9I9M5 | Frizzled receptor | Frizzled-1 |
| Fr001 | O70421 | Frizzled receptor | Frizzled-1 |
| Fr001 | Q08463 | Frizzled receptor | Frizzled-1 |
| Fr001 | O57328 | Frizzled receptor | Frizzled-1 |
| Fr001 | Q9UP38 | Frizzled receptor | Frizzled-1 |
| Fr001 | Q14332 | Frizzled receptor | Frizzled-2 |
| Fr001 | Q9JIP6 | Frizzled receptor | Frizzled-2 |
| Fr001 | Q9PUU6 | Frizzled receptor | Frizzled-2 |
| Fr001 | Q9IA06 | Frizzled receptor | Frizzled-2 |
| Fr001 | O75084 | Frizzled receptor | Frizzled-7 |
| Fr001 | Q5BL72 | Frizzled receptor | Frizzled-7 |
| Fr001 | O57329 | Frizzled receptor | Frizzled-7 |
| Fr001 | Q61090 | Frizzled receptor | Frizzled-7 |
| Fr001 | Q9PUK8 | Frizzled receptor | Frizzled-7 |
| Fr001 | Q8AVJ9 | Frizzled receptor | Frizzled-7 |
| Fr002 | G5ECQ2 | Frizzled receptor | Frizzled-2 |
| Fr002 | Q9VVX3 | Frizzled receptor | Frizzled-2 |
| Fr002 | P58421 | Frizzled receptor | Frizzled-5 |
| Fr002 | Q9EQD0 | Frizzled receptor | Frizzled-5 |
| Fr002 | Q8CHL0 | Frizzled receptor | Frizzled-5 |
| Fr002 | Q13467 | Frizzled receptor | Frizzled-5 |
| Fr002 | Q9H461 | Frizzled receptor | Frizzled-8 |
| Fr002 | Q9IA03 | Frizzled receptor | Frizzled-8 |
| Fr002 | Q61091 | Frizzled receptor | Frizzled-8 |
| Fr002 | Q498S8 | Frizzled receptor | Frizzled-8 |
| Fr002 | O93274 | Frizzled receptor | Frizzled-8 |
| Fr003 | Q8BKG4 | Frizzled receptor | Frizzled-10 |
| Fr003 | Q9ULW2 | Frizzled receptor | Frizzled-10 |
| Fr003 | Q9PWH2 | Frizzled receptor | Frizzled-10 |
| Fr003 | Q9DEB5 | Frizzled receptor | Frizzled-10 |
| Fr003 | Q9W742 | Frizzled receptor | Frizzled-10 |
| Fr003 | O77438 | Frizzled receptor | Frizzled-3 |
| Fr003 | Q9IA02 | Frizzled receptor | Frizzled-9 |
| Fr003 | Q8K4C8 | Frizzled receptor | Frizzled-9 |
| Fr003 | O00144 | Frizzled receptor | Frizzled-9 |
| Fr003 | Q9R216 | Frizzled receptor | Frizzled-9 |
| Fr004 | Q9PTW3 | Frizzled receptor | Frizzled-3 |
| Fr004 | Q61086 | Frizzled receptor | Frizzled-3 |
| Fr004 | O42579 | Frizzled receptor | Frizzled-3 |
| Fr004 | Q9NPG1 | Frizzled receptor | Frizzled-3 |
| Fr004 | Q9PTW1 | Frizzled receptor | Frizzled-6 |
| Fr004 | Q5RCN4 | Frizzled receptor | Frizzled-6 |
| Fr004 | O60353 | Frizzled receptor | Frizzled-6 |
| Fr004 | Q8WMU5 | Frizzled receptor | Frizzled-6 |
| Fr004 | Q61089 | Frizzled receptor | Frizzled-6 |
| Fr005 | Q54P68 | Frizzled receptor | Frizzled and smoothened-like protein |
| Fr005 | Q54H37 | Frizzled receptor | Frizzled/smoothened-like sans CRD protein |
| Fr005 | Q54RQ3 | Frizzled receptor | Frizzled/smoothened-like sans CRD protein |
| Fr005 | Q1ZXA7 | Frizzled receptor | Frizzled/smoothened-like sans CRD protein |
| Fr005 | Q75JP9 | Frizzled receptor | Frizzled/smoothened-like sans CRD protein |
| Fr005 | Q559M5 | Frizzled receptor | Frizzled/smoothened-like sans CRD protein |
| Fr005 | Q54DP3 | Frizzled receptor | Frizzled/smoothened-like sans CRD protein |
| Fr005 | Q54DP2 | Frizzled receptor | Frizzled/smoothened-like sans CRD protein |
| Fr006 | Q9NBW1 | Frizzled receptor | Frizzled-4 |
| Fr006 | Q9ULV1 | Frizzled receptor | Frizzled-4 |
| Fr006 | Q9PT62 | Frizzled receptor | Frizzled-4 |
| Fr006 | Q9QZH0 | Frizzled receptor | Frizzled-4 |
| Fr006 | Q9IA05 | Frizzled receptor | Frizzled-4 |
| Fr006 | Q61088 | Frizzled receptor | Frizzled-4 |
| Fr007 | Q55CD6 | Frizzled receptor | Frizzled and smoothened-like protein |
| Fr007 | Q86J18 | Frizzled receptor | Frizzled and smoothened-like protein |
| Fr007 | Q55DT7 | Frizzled receptor | Frizzled and smoothened-like protein |
| Fr007 | Q54J78 | Frizzled receptor | Frizzled and smoothened-like protein |
| Fr007 | Q54J77 | Frizzled receptor | Frizzled and smoothened-like protein |
| Fr007 | Q54J71 | Frizzled receptor | Frizzled and smoothened-like protein |
| Fr008 | Q54PA5 | Frizzled receptor | Frizzled and smoothened-like protein |
| Fr008 | Q54PF8 | Frizzled receptor | Frizzled and smoothened-like protein |
| Fr008 | Q556N7 | Frizzled receptor | Frizzled and smoothened-like protein |
| Fr009 | Q556C6 | Frizzled receptor | Frizzled and smoothened-like protein |
| Fr009 | Q556J4 | Frizzled receptor | Frizzled and smoothened-like protein |
| Fr010 | Q1ZXB0 | Frizzled receptor | Frizzled/smoothened-like sans CRD protein |
| Fr010 | Q54DR6 | Frizzled receptor | Frizzled/smoothened-like sans CRD protein |
| Fr011 | Q54LJ7 | Frizzled receptor | Frizzled and smoothened-like protein |
| Fr011 | Q1ZXE4 | Frizzled receptor | Frizzled and smoothened-like protein |
| Fr012 | Q55CY2 | Frizzled receptor | Frizzled and smoothened-like protein |
| Fr012 | Q1ZXI9 | Frizzled receptor | Frizzled and smoothened-like protein |
| Fr013 | Q24760 | Frizzled receptor | Frizzled-1 |
| Fr013 | P18537 | Frizzled receptor | Frizzled-1 |
| GB001 | Q9WV18 | GABA-B receptor | GABA-B subtype 1 |
| GB001 | Q9UBS5 | GABA-B receptor | GABA-B subtype 1 |
| GB001 | Q9Z0U4 | GABA-B receptor | GABA-B subtype 1 |
| GB002 | O75899 | GABA-B receptor | GABA-B subtype 2 |
| GB002 | O88871 | GABA-B receptor | GABA-B subtype 2 |
| GB002 | Q80T41 | GABA-B receptor | GABA-B subtype 2 |
| Gi001 | Q0P543 | Gastric inhibitory polypeptide receptor | Gastric inhibitory peptide |
| Gi001 | P48546 | Gastric inhibitory polypeptide receptor | Gastric inhibitory peptide |
| Gi001 | P43218 | Gastric inhibitory polypeptide receptor | Gastric inhibitory peptide |
| Gi001 | P43219 | Gastric inhibitory polypeptide receptor | Gastric inhibitory peptide |
| Gl001 | O35659 | Glucagon receptor | Glucagon |
| Gl001 | P32301 | Glucagon receptor | Glucagon |
| Gl001 | P43220 | Glucagon receptor | Glucagon |
| Gl002 | Q5IXF8 | Glucagon receptor | Glucagon |
| Gl002 | O95838 | Glucagon receptor | Glucagon |
| Gl002 | Q9Z0W0 | Glucagon receptor | Glucagon |
| Gl003 | Q61606 | Glucagon receptor | Glucagon |
| Gl003 | P47871 | Glucagon receptor | Glucagon |
| Gl003 | P30082 | Glucagon receptor | Glucagon |
| Go001 | Q01776 | Gonadotrophin-releasing hormone receptor | Gonadotropin-releasing hormone type I |
| Go001 | Q9MZI6 | Gonadotrophin-releasing hormone receptor | Gonadotropin-releasing hormone type I |
| Go001 | P30968 | Gonadotrophin-releasing hormone receptor | Gonadotropin-releasing hormone type I |
| Go001 | P32236 | Gonadotrophin-releasing hormone receptor | Gonadotropin-releasing hormone type I |
| Go001 | O18821 | Gonadotrophin-releasing hormone receptor | Gonadotropin-releasing hormone type I |
| Go001 | P32237 | Gonadotrophin-releasing hormone receptor | Gonadotropin-releasing hormone type I |
| Go001 | P49922 | Gonadotrophin-releasing hormone receptor | Gonadotropin-releasing hormone type I |
| Go001 | Q9TTI8 | Gonadotrophin-releasing hormone receptor | Gonadotropin-releasing hormone type I |
| Go001 | Q8CH60 | Gonadotrophin-releasing hormone receptor | Gonadotropin-releasing hormone type I |
| Go001 | Q19PY9 | Gonadotrophin-releasing hormone receptor | Gonadotropin-releasing hormone type I |
| Go001 | P30969 | Gonadotrophin-releasing hormone receptor | Gonadotropin-releasing hormone type I |
| Go002 | Q95MG6 | Gonadotrophin-releasing hormone receptor | Gonadotropin-releasing hormone type II |
| Go002 | Q95MH6 | Gonadotrophin-releasing hormone receptor | Gonadotropin-releasing hormone type II |
| Go002 | Q95JG1 | Gonadotrophin-releasing hormone receptor | Gonadotropin-releasing hormone type II |
| Go002 | O42329 | Gonadotrophin-releasing hormone receptor | Gonadotropin-releasing hormone type II |
| GPR001 | Q8K209 | GPR56 | GPR56 |
| GPR001 | Q9Y653 | GPR56 | GPR56 |
| GPR001 | Q8K3V3 | GPR56 | GPR56 |
| GPR001 | Q50DM8 | GPR56 | GPR56 |
| GPR001 | Q50DM7 | GPR56 | GPR56 |
| GPR001 | Q50DM6 | GPR56 | GPR56 |
| GPR001 | Q50DM5 | GPR56 | GPR56 |
| GPR002 | Q8VEC3 | GPR110 | GPR110 |
| GPR002 | Q5T601 | GPR110 | GPR110 |
| GPR003 | Q3V3Z3 | GPR114 | GPR114 |
| GPR003 | Q8IZF4 | GPR114 | GPR114 |
| GPR004 | Q86Y34 | GPR97 | GPR97 |
| GPR004 | Q8R0T6 | GPR97 | GPR97 |
| GPR005 | Q8IZF5 | GPR113 | GPR113 |
| GPR005 | Q58Y75 | GPR113 | GPR113 |
| GPR006 | Q8BM96 | GPR128 | GPR128 |
| GPR006 | Q96K78 | GPR128 | GPR128 |
| Gr001 | P32082 | Growth hormone-releasing hormone receptor | Growth hormone-releasing hormone |
| Gr001 | Q02643 | Growth hormone-releasing hormone receptor | Growth hormone-releasing hormone |
| Gr001 | P34999 | Growth hormone-releasing hormone receptor | Growth hormone-releasing hormone |
| Gr001 | Q02644 | Growth hormone-releasing hormone receptor | Growth hormone-releasing hormone |
| Ha001 | P49019 | Hydroxycarboxylic acid receptor | GPR109 |
| Ha001 | Q8TDS4 | Hydroxycarboxylic acid receptor | GPR109 |
| Ha001 | Q9EP66 | Hydroxycarboxylic acid receptor | GPR109 |
| Ha001 | Q80Z39 | Hydroxycarboxylic acid receptor | GPR109 |
| Ha002 | Q9BXC0 | Hydroxycarboxylic acid receptor | GPR81 |
| Ha002 | Q8C131 | Hydroxycarboxylic acid receptor | GPR81 |
| Ho001 | Q95179 | Hormone receptor | Follicle stimulating hormone |
| Ho001 | P56495 | Hormone receptor | Thyrotropin |
| Ho001 | P23945 | Hormone receptor | Follicle stimulating hormone |
| Ho001 | O02721 | Hormone receptor | Lutropin-choriogonadotropic hormone |
| Ho001 | P16582 | Hormone receptor | Lutropin-choriogonadotropic hormone |
| Ho001 | P79763 | Hormone receptor | Follicle stimulating hormone |
| Ho001 | Q6YNB6 | Hormone receptor | Follicle stimulating hormone |
| Ho001 | P35378 | Hormone receptor | Follicle stimulating hormone |
| Ho001 | Q28005 | Hormone receptor | Lutropin-choriogonadotropic hormone |
| Ho001 | P16235 | Hormone receptor | Lutropin-choriogonadotropic hormone |
| Ho001 | P47799 | Hormone receptor | Follicle stimulating hormone |
| Ho001 | Q6R6L8 | Hormone receptor | Follicle stimulating hormone |
| Ho001 | Q8SPP9 | Hormone receptor | Thyrotropin |
| Ho001 | Q6QMG1 | Hormone receptor | Thyrotropin |
| Ho001 | Q28585 | Hormone receptor | Lutropin-choriogonadotropic hormone |
| Ho001 | P32212 | Hormone receptor | Follicle stimulating hormone |
| Ho001 | P35409 | Hormone receptor | Thyrotropin |
| Ho001 | P35379 | Hormone receptor | Follicle stimulating hormone |
| Ho001 | P47750 | Hormone receptor | Thyrotropin |
| Ho001 | Q8R428 | Hormone receptor | Follicle stimulating hormone |
| Ho001 | P14763 | Hormone receptor | Thyrotropin |
| Ho001 | P30730 | Hormone receptor | Lutropin-choriogonadotropic hormone |
| Ho001 | P35376 | Hormone receptor | Follicle stimulating hormone |
| Ho001 | Q5GJ04 | Hormone receptor | Follicle stimulating hormone |
| Ho001 | Q27987 | Hormone receptor | Thyrotropin |
| Ho001 | P21463 | Hormone receptor | Thyrotropin |
| Ho001 | Q7ZTV5 | Hormone receptor | Follicle stimulating hormone |
| Ho001 | P16473 | Hormone receptor | Thyrotropin |
| Ho001 | P49059 | Hormone receptor | Follicle stimulating hormone |
| Ho001 | P22888 | Hormone receptor | Lutropin-choriogonadotropic hormone |
| Ho001 | P20395 | Hormone receptor | Follicle stimulating hormone |
| Ho001 | Q9BGN4 | Hormone receptor | Thyrotropin |
| La001 | Q80TR1 | Latrophilin receptor | Latrophilin |
| La001 | O88917 | Latrophilin receptor | Latrophilin |
| La001 | O94910 | Latrophilin receptor | Latrophilin |
| La001 | O97831 | Latrophilin receptor | Latrophilin |
| La001 | O97817 | Latrophilin receptor | Latrophilin |
| La001 | O88923 | Latrophilin receptor | Latrophilin |
| La001 | O95490 | Latrophilin receptor | Latrophilin |
| La001 | O97827 | Latrophilin receptor | Latrophilin receptor |
| La001 | Q9Z173 | Latrophilin receptor | Latrophilin receptor |
| La001 | Q9HAR2 | Latrophilin receptor | Latrophilin receptor |
| La001 | Q80TS3 | Latrophilin receptor | Latrophilin receptor |
| La002 | B3MFV7 | Latrophilin receptor | Latrophilin |
| La002 | B3N8M1 | Latrophilin receptor | Latrophilin |
| La002 | B4J780 | Latrophilin receptor | Latrophilin |
| La002 | B4KMZ1 | Latrophilin receptor | Latrophilin |
| La002 | B4GD14 | Latrophilin receptor | Latrophilin |
| La002 | B4LNA8 | Latrophilin receptor | Latrophilin |
| La002 | B4P3A0 | Latrophilin receptor | Latrophilin |
| La002 | Q292N4 | Latrophilin receptor | Latrophilin |
| La002 | A1Z7G7 | Latrophilin receptor | Latrophilin |
| La002 | B4HS00 | Latrophilin receptor | Latrophilin |
| La003 | Q9ESC1 | Latrophilin receptor | Latrophilin |
| La003 | Q923X1 | Latrophilin receptor | Latrophilin |
| La003 | Q9HBW9 | Latrophilin receptor | Latrophilin |
| Le001 | Q9JJL9 | Leukotriene B4 receptor | Leukotriene B4 receptor type 2 |
| Le001 | Q924U0 | Leukotriene B4 receptor | Leukotriene B4 receptor type 2 |
| Le001 | Q9NPC1 | Leukotriene B4 receptor | Leukotriene B4 receptor type 2 |
| Le002 | Q3T181 | Leukotriene B4 receptor | Leukotriene B4 receptor type 1 |
| Le002 | Q15722 | Leukotriene B4 receptor | Leukotriene B4 receptor type 1 |
| Le003 | Q9R0Q2 | Leukotriene B4 receptor | Leukotriene B4 receptor type 1 |
| Le003 | O88855 | Leukotriene B4 receptor | Leukotriene B4 receptor type 1 |
| Ly001 | Q9PU17 | Lysophospholipid (LPA) receptor | Lysophosphatidic acid Edg-2 |
| Ly001 | P61793 | Lysophospholipid (LPA) receptor | Lysophosphatidic acid Edg-2 |
| Ly001 | P61794 | Lysophospholipid (LPA) receptor | Lysophosphatidic acid Edg-2 |
| Ly001 | Q92633 | Lysophospholipid (LPA) receptor | Lysophosphatidic acid Edg-2 |
| Ly001 | Q9PU16 | Lysophospholipid (LPA) receptor | Lysophosphatidic acid Edg-2 |
| Ly001 | Q28031 | Lysophospholipid (LPA) receptor | Lysophosphatidic acid Edg-2 |
| Ly001 | P46628 | Lysophospholipid (LPA) receptor | Lysophosphatidic acid Edg-2 |
| Ly002 | Q9DDK4 | Lysophospholipid (LPA) receptor | Sphingosine 1-phosphate Edg-1 |
| Ly002 | P21453 | Lysophospholipid (LPA) receptor | Sphingosine 1-phosphate Edg-1 |
| Ly002 | O08530 | Lysophospholipid (LPA) receptor | Sphingosine 1-phosphate Edg-1 |
| Ly002 | Q5E9P3 | Lysophospholipid (LPA) receptor | Sphingosine 1-phosphate Edg-1 |
| Ly002 | P48303 | Lysophospholipid (LPA) receptor | Sphingosine 1-phosphate Edg-1 |
| Ly003 | Q9I8K8 | Lysophospholipid (LPA) receptor | Sphingosine 1-phosphate Edg-5 |
| Ly003 | P47752 | Lysophospholipid (LPA) receptor | Sphingosine 1-phosphate Edg-5 |
| Ly003 | P52592 | Lysophospholipid (LPA) receptor | Sphingosine 1-phosphate Edg-5 |
| Ly003 | O95136 | Lysophospholipid (LPA) receptor | Sphingosine 1-phosphate Edg-5 |
| Ly004 | Q9H228 | Lysophospholipid (LPA) receptor | Sphingosine 1-phosphate Edg-8 |
| Ly004 | Q9JKM5 | Lysophospholipid (LPA) receptor | Sphingosine 1-phosphate Edg-8 |
| Ly004 | Q684M3 | Lysophospholipid (LPA) receptor | Sphingosine 1-phosphate Edg-8 |
| Ly004 | Q91X56 | Lysophospholipid (LPA) receptor | Sphingosine 1-phosphate Edg-8 |
| Ly005 | Q99500 | Lysophospholipid (LPA) receptor | Sphingosine 1-phosphate Edg-3 |
| Ly005 | Q9Z0U9 | Lysophospholipid (LPA) receptor | Sphingosine 1-phosphate Edg-3 |
| Ly005 | Q9PUQ8 | Lysophospholipid (LPA) receptor | Sphingosine 1-phosphate Edg-3 |
| Ly006 | Q9EQ31 | Lysophospholipid (LPA) receptor | Lysophosphatidic acid Edg-7 |
| Ly006 | Q9UBY5 | Lysophospholipid (LPA) receptor | Lysophosphatidic acid Edg-7 |
| Ly006 | Q8K5E0 | Lysophospholipid (LPA) receptor | Lysophosphatidic acid Edg-7 |
| Ly007 | Q9HBW0 | Lysophospholipid (LPA) receptor | Lysophosphatidic acid Edg-4 |
| Ly007 | Q9JL06 | Lysophospholipid (LPA) receptor | Lysophosphatidic acid Edg-4 |
| Ly007 | Q95KH4 | Lysophospholipid (LPA) receptor | Lysophosphatidic acid Edg-4 |
| Ly008 | Q9EQQ4 | Lysophospholipid (LPA) receptor | GPR45 |
| Ly008 | Q9Y5Y3 | Lysophospholipid (LPA) receptor | GPR45 |
| Ly008 | P79945 | Lysophospholipid (LPA) receptor | GPR45 |
| Ly009 | O95977 | Lysophospholipid (LPA) receptor | Sphingosine 1-phosphate Edg-6 |
| Ly009 | Q9Z0L1 | Lysophospholipid (LPA) receptor | Sphingosine 1-phosphate Edg-6 |
| Ly010 | Q9BZJ6 | Lysophospholipid (LPA) receptor | GPR63 |
| Ly010 | Q9EQQ3 | Lysophospholipid (LPA) receptor | GPR63 |
| Me001 | P48039 | Melatonin receptor | Melatonin type 1 |
| Me001 | P49217 | Melatonin receptor | Melatonin type 1 |
| Me001 | P51049 | Melatonin receptor | Melatonin type 1 |
| Me001 | Q61184 | Melatonin receptor | Melatonin type 1 |
| Me001 | Q90456 | Melatonin receptor | Melatonin type 1 |
| Me001 | P51050 | Melatonin receptor | Melatonin type 1 |
| Me001 | O02769 | Melatonin receptor | Melatonin type 1 |
| Me001 | P49285 | Melatonin receptor | Melatonin type 1 |
| Me001 | P48040 | Melatonin receptor | Melatonin type 1 |
| Me001 | P51046 | Melatonin receptor | Melatonin type 1 |
| Me002 | O88495 | Melatonin receptor | Melatonin-like (GPR50) |
| Me002 | Q28558 | Melatonin receptor | Melatonin-like (GPR50) |
| Me002 | Q13585 | Melatonin receptor | Melatonin-like (GPR50) |
| Me003 | P49219 | Melatonin receptor | Melatonin type 1 |
| Me003 | P49288 | Melatonin receptor | Melatonin type 1 |
| Me004 | P49286 | Melatonin receptor | Melatonin type 1 |
| Me004 | Q8CIQ6 | Melatonin receptor | Melatonin type 1 |
| MiG001 | P97772 | Metabotropic glutamate receptor | Metabotropic glutamate type 1 3 |
| MiG001 | P23385 | Metabotropic glutamate receptor | Metabotropic glutamate type 1 3 |
| MiG001 | Q13255 | Metabotropic glutamate receptor | Metabotropic glutamate type 1 3 |
| MiG001 | P41594 | Metabotropic glutamate receptor | Metabotropic glutamate type 5 2 |
| MiG001 | P31424 | Metabotropic glutamate receptor | Metabotropic glutamate type 5 2 |
| MiG001 | Q3UVX5 | Metabotropic glutamate receptor | Metabotropic glutamate type 5 2 |
| MiG001 | Q14BI2 | Metabotropic glutamate receptor | Metabotropic glutamate type 2 3 |
| MiG001 | P31421 | Metabotropic glutamate receptor | Metabotropic glutamate type 2 3 |
| MiG001 | Q14416 | Metabotropic glutamate receptor | Metabotropic glutamate type 2 3 |
| MiG001 | P35349 | Metabotropic glutamate receptor | Metabotropic glutamate type 6 |
| MiG001 | O15303 | Metabotropic glutamate receptor | Metabotropic glutamate type 6 |
| MiG001 | Q863I4 | Metabotropic glutamate receptor | Metabotropic glutamate type 6 |
| MiG001 | Q5NCH9 | Metabotropic glutamate receptor | Metabotropic glutamate type 6 |
| MiG001 | Q14833 | Metabotropic glutamate receptor | Metabotropic glutamate type 8 |
| MiG001 | Q14831 | Metabotropic glutamate receptor | Metabotropic glutamate type 8 |
| MiG001 | P31423 | Metabotropic glutamate receptor | Metabotropic glutamate type 8 |
| MiG001 | P35400 | Metabotropic glutamate receptor | Metabotropic glutamate type 8 |
| MiG001 | O00222 | Metabotropic glutamate receptor | Metabotropic glutamate type 8 |
| MiG001 | P70579 | Metabotropic glutamate receptor | Metabotropic glutamate type 8 |
| MiG001 | P47743 | Metabotropic glutamate receptor | Metabotropic glutamate type 8 |
| MiG001 | Q68ED2 | Metabotropic glutamate receptor | Metabotropic glutamate type 8 |
| MiG001 | Q5RDQ8 | Metabotropic glutamate receptor | Metabotropic glutamate type 8 |
| MiG001 | Q1ZZH0 | Metabotropic glutamate receptor | Metabotropic glutamate type 8 |
| MiG001 | Q68EF4 | Metabotropic glutamate receptor | Metabotropic glutamate type 8 |
| MiG001 | Q9QYS2 | Metabotropic glutamate receptor | Metabotropic glutamate type 3 1 |
| MiG001 | P31422 | Metabotropic glutamate receptor | Metabotropic glutamate type 3 1 |
| MiG001 | Q14832 | Metabotropic glutamate receptor | Metabotropic glutamate type 3 1 |
| MiG001 | Q5RAL3 | Metabotropic glutamate receptor | Metabotropic glutamate type 3 1 |
| MiG001 | Q1ZZH1 | Metabotropic glutamate receptor | Metabotropic glutamate type 3 1 |
| MiG001 | Q09630 | Class C Other | Other 16 |
| MiG001 | P91685 | Metabotropic glutamate receptor | Metabotropic glutamate receptor |
| MiG002 | Q7RTX1 | Class C Other | Taste receptor type 1 |
| MiG002 | Q99PG6 | Class C Other | Taste receptor type 1 |
| MiG002 | Q9Z0R8 | Class C Other | Taste receptor type 1 |
| MiG002 | Q925I4 | Taste receptor type 1 | Taste 2 |
| MiG002 | Q9Z0R7 | Taste receptor type 1 | Taste 2 |
| MiG002 | Q8TE23 | Taste receptor type 1 | Taste 2 |
| MiG002 | Q49HI0 | Taste receptor type 1 | Taste 2 |
| MiG002 | A3QNZ8 | Taste receptor type 1 | Taste 2 |
| MiG002 | A3QNZ9 | Taste receptor type 1 | Taste 2 |
| MiG002 | A3QP00 | Taste receptor type 1 | Taste 2 |
| MiG002 | A3QP01 | Taste receptor type 1 | Taste 2 |
| MiG002 | A3QP07 | Taste receptor type 1 | Taste 2 |
| MiG002 | A3QP08 | Taste receptor type 1 | Taste 2 |
| MiG003 | Q6TAC4 | Vomeronasal type-2 receptor 1b | Vomeronasal 9 |
| MiG003 | Q9QY96 | Calcium-sensing receptor | Calcium sensing 1 |
| MiG003 | P35384 | Calcium-sensing receptor | Calcium sensing 1 |
| MiG003 | P41180 | Calcium-sensing receptor | Calcium sensing 1 |
| MiG003 | P48442 | Calcium-sensing receptor | Calcium sensing 1 |
| MiG003 | O70410 | Calcium-sensing receptor | Calcium sensing 2 |
| MiG004 | Q5T6X5 | Class C Odorant | Odorant 5 |
| MiG004 | Q5U9X3 | Class C Odorant | Odorant 4 |
| MiG004 | Q8K4Z6 | Class C Other | Other 6 |
| MiG004 | Q70VB1 | Class C Other | Other 6 |
| MiG004 | Q9PW88 | Class C Other | Other 5 |
| MiR001 | Q6R6I7 | Class A Orphan/other | LGR like (hormone receptors) type 7 and 8 |
| MiR001 | Q5XM32 | Class A Orphan/other | LGR like (hormone receptors) type 7 and 8 |
| MiR001 | Q6R6I6 | Class A Orphan/other | LGR like (hormone receptors) type 7 and 8 |
| MiR001 | Q91ZZ5 | Class A Orphan/other | LGR like (hormone receptors) type 7 and 8 |
| MiR001 | P46023 | Hormone receptor | Gonadotropin type I |
| MiR001 | Q9HBX9 | Class A Orphan/other | LGR like (hormone receptors) type 7 and 8 |
| MiR001 | Q5R5V8 | Class A Orphan/other | LGR like (hormone receptors) type 7 and 8 |
| MiR001 | Q8WXD0 | Class A Orphan/other | LGR like (hormone receptors) type 7 and 8 |
| MiR002 | Q923Y7 | Aminergic receptor | Trace amine type 4 |
| MiR002 | Q5QNP2 | Class A Orphan/other | Other 509 |
| MiR002 | Q5QD15 | Aminergic receptor | Trace amine type 4 |
| MiR003 | Q96P66 | Peptide receptor | C-C Chemokine type 5 |
| MiR003 | Q80T62 | Gonadotrophin-releasing hormone receptor | Gonadotropin-releasing hormone type II |
| MiS001 | Q9UHX3 | EGF-like module receptor 1 | ERM1 |
| MiS001 | Q2Q426 | EGF-like module receptor 1 | ERM1 |
| MiS001 | Q2Q421 | EGF-like module receptor 1 | ERM1 |
| MiS001 | Q9BY15 | EGF-like module receptor 1 | ERM1 |
| MiS001 | Q86SQ3 | EGF-like module receptor 1 | ERM1 |
| MiS001 | Q91ZE5 | EGF-like module receptor 1 | ERM1 |
| MiS001 | P48960 | CD97 | CD97 |
| MiS001 | Q9Z0M6 | CD97 | CD97 |
| MiS001 | Q8SQA4 | CD97 | CD97 |
| MiS002 | Q8CJ12 | GPR64 | GPR64 |
| MiS002 | Q8CJ11 | GPR64 | GPR64 |
| MiS002 | Q8IZP9 | GPR64 | GPR64 |
| MiS002 | Q6F3F9 | GPR126 | GPR126 |
| MiS002 | Q86SQ4 | GPR126 | GPR126 |
| MiS002 | C6KFA3 | GPR126 | GPR126 |
| MiS003 | Q8IWK6 | GPR124 | GPR124 |
| MiS003 | Q7TT36 | GPR124 | GPR124 |
| MiS003 | Q96PE1 | GPR124 | GPR124 |
| MiS003 | Q91ZV8 | GPR124 | GPR124 |
| MiS003 | Q86SQ6 | GPR123 | GPR123 |
| MiS004 | Q80T32 | GPR133 | GPR133 |
| MiS004 | A6QLU6 | GPR133 | GPR133 |
| MiS004 | Q6QNK2 | To be sorted | To be sorted |
| MiS005 | B7ZCC9 | GPR126 | GPR126 |
| MiS005 | Q8IZF6 | GPR64 | GPR64 |
| MiV001 | Q8NFZ6 | Vomeronasal type-1 receptor L | Vomeronasal receptors V1RL |
| MiV001 | Q7Z5H5 | Vomeronasal type-1 receptor F | Vomeronasal receptors V1RF |
| MiV001 | Q7YRP2 | Vomeronasal type-1 receptor F | Vomeronasal receptors V1RF |
| MiV002 | Q5J3M4 | Vomeronasal type-1 receptor A | Vomeronasal receptors V1RA |
| MiV002 | Q5J3L6 | Vomeronasal type-1 receptor A | Vomeronasal receptors V1RA |
| MiV002 | Q5J3F6 | Vomeronasal type-1 receptor other | Vomeronasal receptors other |
| Ml001 | O97148 | Methuselah-like proteins | Methuselah-like proteins (MTH) type 1 |
| Ml001 | P83118 | Methuselah-like proteins | Methuselah-like proteins (MTH) type 1 |
| Ml001 | Q9VRN2 | Methuselah-like proteins | Methuselah-like proteins (MTH) type 1 |
| Ml001 | Q9GT50 | Methuselah-like proteins | Methuselah-like proteins (MTH) type 1 |
| Ml001 | P83120 | Methuselah-like proteins | Methuselah-like proteins (MTH) type 1 |
| Ml001 | Q9V818 | Methuselah-like proteins | Methuselah-like proteins (MTH) type 3 |
| Ml001 | Q9V817 | Methuselah-like proteins | Methuselah-like proteins (MTH) type 4 |
| Ml002 | Q9VS77 | Methuselah-like proteins | Methuselah-like proteins (MTH) type 1 |
| Ml002 | Q9VSE7 | Methuselah-like proteins | Methuselah-like proteins (MTH) type 1 |
| Ml002 | P83119 | Methuselah-like proteins | Methuselah-like proteins (MTH) type 1 |
| Ml003 | Q95NT6 | Methuselah-like proteins | Methuselah-like proteins (MTH) type 2 |
| Ml003 | Q95NQ0 | Methuselah-like proteins | Methuselah-like proteins (MTH) type 2 |
| Ml003 | Q9W0R5 | Methuselah-like proteins | Methuselah-like proteins (MTH) type 2 |
| Nu001 | P28190 | Nucleotide receptor | Adenosine type 1 |
| Nu001 | P11616 | Nucleotide receptor | Adenosine type 1 |
| Nu001 | P49892 | Nucleotide receptor | Adenosine type 1 |
| Nu001 | P34970 | Nucleotide receptor | Adenosine type 1 |
| Nu001 | P47745 | Nucleotide receptor | Adenosine type 1 |
| Nu001 | Q5RF57 | Nucleotide receptor | Adenosine type 1 |
| Nu001 | Q60612 | Nucleotide receptor | Adenosine type 1 |
| Nu001 | P30542 | Nucleotide receptor | Adenosine type 1 |
| Nu001 | P25099 | Nucleotide receptor | Adenosine type 1 |
| Nu002 | Q60614 | Nucleotide receptor | Adenosine type 2 |
| Nu002 | P29276 | Nucleotide receptor | Adenosine type 2 |
| Nu002 | P29275 | Nucleotide receptor | Adenosine type 2 |
| Nu002 | Q32ZE2 | Nucleotide receptor | Adenosine type 2 |
| Nu002 | Q1LZD0 | Nucleotide receptor | Adenosine type 2 |
| Nu002 | O13076 | Nucleotide receptor | Adenosine type 2 |
| Nu002 | Q6W3F4 | Nucleotide receptor | Adenosine type 2 |
| Nu003 | P49651 | Nucleotide receptor | P2RY1 |
| Nu003 | P34996 | Nucleotide receptor | P2RY1 |
| Nu003 | P49650 | Nucleotide receptor | P2RY1 |
| Nu003 | P49652 | Nucleotide receptor | P2RY1 |
| Nu003 | P47900 | Nucleotide receptor | P2RY1 |
| Nu003 | P48042 | Nucleotide receptor | P2RY1 |
| Nu003 | P59902 | Nucleotide receptor | P2RY1 |
| Nu004 | P29274 | Nucleotide receptor | Adenosine type 2 |
| Nu004 | P11617 | Nucleotide receptor | Adenosine type 2 |
| Nu004 | P30543 | Nucleotide receptor | Adenosine type 2 |
| Nu004 | Q60613 | Nucleotide receptor | Adenosine type 2 |
| Nu004 | Q6TLI7 | Nucleotide receptor | Adenosine type 2 |
| Nu004 | P46616 | Nucleotide receptor | Adenosine type 2 |
| Nu005 | Q0VC81 | Nucleotide receptor | Adenosine type 3 |
| Nu005 | O02667 | Nucleotide receptor | Adenosine type 3 |
| Nu005 | P33765 | Nucleotide receptor | Adenosine type 3 |
| Nu005 | P35342 | Nucleotide receptor | Adenosine type 3 |
| Nu005 | Q28309 | Nucleotide receptor | Adenosine type 3 |
| Nu006 | Q8BUD0 | Nucleotide receptor | P2RY5 |
| Nu006 | Q4KLH9 | Nucleotide receptor | P2RY5 |
| Nu006 | Q1JQB3 | Nucleotide receptor | P2RY5 |
| Nu006 | P46093 | Nucleotide receptor | P2RY5 |
| Nu006 | P50132 | Nucleotide receptor | P2RY5 |
| Nu007 | B3DM66 | Nucleotide receptor | Adenosine type 1 |
| Nu007 | B2RPY5 | Nucleotide receptor | Adenosine type 1 |
| Nu007 | Q90X46 | Nucleotide receptor | Adenosine type 1 |
| Nu007 | Q8N6U8 | Nucleotide receptor | Adenosine type 1 |
| Nu007 | Q2YDN1 | Nucleotide receptor | Adenosine type 1 |
| Nu008 | Q9JJS7 | Nucleotide receptor | P2RY4 |
| Nu008 | P51582 | Nucleotide receptor | P2RY4 |
| Nu008 | O35811 | Nucleotide receptor | P2RY4 |
| Nu008 | P79928 | Nucleotide receptor | P2RY4 |
| Nu009 | Q9CPV9 | Nucleotide receptor | Purinoceptor P2RY12-14 GPR87 (UDP-Glucose) |
| Nu009 | Q9H244 | Nucleotide receptor | Purinoceptor P2RY12-14 GPR87 (UDP-Glucose) |
| Nu009 | Q9EPX4 | Nucleotide receptor | Purinoceptor P2RY12-14 GPR87 (UDP-Glucose) |
| Nu009 | Q95KC3 | Nucleotide receptor | Purinoceptor P2RY12-14 GPR87 (UDP-Glucose) |
| Nu010 | Q8BMC0 | Nucleotide receptor | P2RY5 |
| Nu010 | P43657 | Nucleotide receptor | P2RY5 |
| Nu010 | Q4G072 | Nucleotide receptor | P2RY5 |
| Nu010 | P32250 | Nucleotide receptor | P2RY9 |
| Nu011 | P41232 | Nucleotide receptor | P2RY2 |
| Nu011 | Q5YA25 | Nucleotide receptor | P2RY2 |
| Nu011 | P41231 | Nucleotide receptor | P2RY2 |
| Nu011 | P35383 | Nucleotide receptor | P2RY2 |
| Nu012 | Q9ESG6 | Nucleotide receptor | Purinoceptor P2RY12-14 GPR87 (UDP-Glucose) |
| Nu012 | O35881 | Nucleotide receptor | Purinoceptor P2RY12-14 GPR87 (UDP-Glucose) |
| Nu012 | Q3SX17 | Nucleotide receptor | Purinoceptor P2RY12-14 GPR87 (UDP-Glucose) |
| Nu012 | Q15391 | Nucleotide receptor | Purinoceptor P2RY12-14 GPR87 (UDP-Glucose) |
| Nu013 | Q149R9 | Nucleotide receptor | P2RY5 |
| Nu013 | Q3ZC80 | Nucleotide receptor | P2RY5 |
| Nu013 | Q9H1C0 | Nucleotide receptor | P2RY5 |
| Nu014 | Q99MT6 | Nucleotide receptor | Succinate receptors |
| Nu014 | Q6IYF9 | Nucleotide receptor | Succinate receptors |
| Nu014 | Q9BXA5 | Nucleotide receptor | Succinate receptors |
| Nu015 | A6QLE7 | Nucleotide receptor | P2RY5 |
| Nu015 | Q9Y2T5 | Nucleotide receptor | P2RY5 |
| Nu015 | P0C5J4 | Nucleotide receptor | P2RY5 |
| Nu016 | Q6IYF8 | Nucleotide receptor | P2RY1 |
| Nu016 | Q6Y1R5 | Nucleotide receptor | P2RY1 |
| Nu016 | Q96P68 | Nucleotide receptor | P2RY1 |
| Nu017 | Q8BFQ3 | Nucleotide receptor | P2RY5 |
| Nu017 | O46685 | Nucleotide receptor | P2RY5 |
| Nu017 | Q15743 | Nucleotide receptor | P2RY5 |
| Nu018 | Q9D8I2 | Nucleotide receptor | Purinoceptor P2RY12-14 GPR87 (UDP-Glucose) |
| Nu018 | Q6GUG4 | Nucleotide receptor | Purinoceptor P2RY12-14 GPR87 (UDP-Glucose) |
| Nu018 | Q9BPV8 | Nucleotide receptor | Purinoceptor P2RY12-14 GPR87 (UDP-Glucose) |
| Nu019 | Q8BG55 | Nucleotide receptor | Purinoceptor P2RY12-14 GPR87 (UDP-Glucose) |
| Nu019 | Q3ZBK9 | Nucleotide receptor | Purinoceptor P2RY12-14 GPR87 (UDP-Glucose) |
| Nu019 | O14626 | Nucleotide receptor | Purinoceptor P2RY12-14 GPR87 (UDP-Glucose) |
| Nu020 | Q9ERK9 | Nucleotide receptor | P2RY3and6 |
| Nu020 | Q63371 | Nucleotide receptor | P2RY3and6 |
| Nu020 | Q15077 | Nucleotide receptor | P2RY3and6 |
| Nu021 | Q9UNW8 | Nucleotide receptor | P2RY5 |
| Nu021 | Q9Z282 | Nucleotide receptor | P2RY5 |
| Nu022 | Q61038 | Nucleotide receptor | P2RY5 |
| Nu022 | Q8IYL9 | Nucleotide receptor | P2RY5 |
| Nu023 | O00398 | Nucleotide receptor | P2RY5 other |
| Nu023 | Q8BFU7 | Nucleotide receptor | P2RY5 other |
| Nu024 | Q99MT7 | Nucleotide receptor | Purinoceptor P2RY12-14 GPR87 (UDP-Glucose) |
| Nu024 | Q9BY21 | Nucleotide receptor | Purinoceptor P2RY12-14 GPR87 (UDP-Glucose) |
| Nu025 | Q8BLG2 | Nucleotide receptor | P2RY9 |
| Nu025 | Q99677 | Nucleotide receptor | P2RY9 |
| Nu026 | Q99679 | Nucleotide receptor | P2RY5 |
| Nu026 | Q8BX79 | Nucleotide receptor | P2RY5 |
| Nu027 | Q99678 | Nucleotide receptor | P2RY5 |
| Nu027 | Q8BYC4 | Nucleotide receptor | P2RY5 |
| Nu028 | Q61618 | Nucleotide receptor | Adenosine type 3 |
| Nu028 | P28647 | Nucleotide receptor | Adenosine type 3 |
| Nu029 | Q8BZL4 | Nucleotide receptor | Purinoceptor P2RY12-14 GPR87 (UDP-Glucose) |
| Nu029 | Q99680 | Nucleotide receptor | Purinoceptor P2RY12-14 GPR87 (UDP-Glucose) |
| Nu030 | Q98907 | Nucleotide receptor | P2RY3and6 |
| Nu030 | O93361 | Nucleotide receptor | P2RY3and6 |
| Ol001 | Q8NGA2 | Olfactory receptor | Olfactory 71 |
| Ol001 | Q95157 | Olfactory receptor | Olfactory 71 |
| Ol001 | O76100 | Olfactory receptor | Olfactory 71 |
| Ol001 | Q9JHB2 | Olfactory receptor | Olfactory 71 |
| Ol001 | P35898 | Olfactory receptor | Olfactory 71 |
| Ol001 | O14581 | Olfactory receptor | Olfactory 71 |
| Ol001 | Q60892 | Olfactory receptor | Olfactory 107 |
| Ol001 | P23265 | Olfactory receptor | Olfactory 108 |
| Ol001 | Q15622 | Olfactory receptor | Olfactory 71 |
| Ol002 | Q9H346 | Olfactory receptor | Olfactory 241 |
| Ol002 | Q6IFG1 | Olfactory receptor | Olfactory 240 |
| Ol002 | Q8NH55 | Olfactory receptor | Olfactory 246 |
| Ol002 | Q8NH57 | Olfactory receptor | Olfactory II fam 12 / MOR250 |
| Ol002 | P0C646 | Olfactory receptor | Olfactory 241 |
| Ol002 | Q96RD3 | Olfactory receptor | Olfactory 240 |
| Ol002 | Q8NGH9 | Olfactory receptor | Olfactory 240 |
| Ol002 | Q8NH60 | Olfactory receptor | Olfactory 246 |
| Ol003 | A6NDH6 | Olfactory receptor | Olfactory 185 |
| Ol003 | Q8VEX6 | Olfactory receptor | Olfactory 185 |
| Ol003 | A6NHG9 | Olfactory receptor | Olfactory 279 |
| Ol003 | A6NKK0 | Olfactory receptor | Olfactory 185 |
| Ol003 | Q8NGV7 | Olfactory receptor | Olfactory 279 |
| Ol003 | Q8NGV6 | Olfactory receptor | Olfactory 279 |
| Ol003 | Q8VFB9 | Olfactory receptor | Olfactory 143 |
| Ol003 | Q8VEX5 | Olfactory receptor | Olfactory 279 |
| Ol004 | Q8WZA6 | Olfactory receptor | Olfactory II fam 1 / MOR125-138,156 |
| Ol004 | P30953 | Olfactory receptor | Olfactory II fam 1 / MOR125-138,156 |
| Ol004 | P30955 | Olfactory receptor | Olfactory 71 |
| Ol004 | Q9TQX4 | Olfactory receptor | Olfactory 71 |
| Ol004 | Q9TU94 | Olfactory receptor | Olfactory 71 |
| Ol004 | Q9TUA2 | Olfactory receptor | Olfactory 71 |
| Ol004 | Q9TUA9 | Olfactory receptor | Olfactory 71 |
| Ol004 | P47887 | Olfactory receptor | Olfactory II fam 1 / MOR125-138,156 |
| Ol005 | Q8NGZ2 | Olfactory receptor | Olfactory 232 |
| Ol005 | Q8NHC6 | Olfactory receptor | Olfactory 232 |
| Ol005 | Q96R54 | Olfactory receptor | Olfactory 293 |
| Ol005 | Q8NHC7 | Olfactory receptor | Olfactory 183 |
| Ol005 | A6ND48 | Olfactory receptor | Olfactory 86 |
| Ol005 | Q8NHC5 | Olfactory receptor | Olfactory 86 |
| Ol005 | Q9UGF5 | Olfactory receptor | Olfactory 201 |
| Ol006 | Q8NH73 | Olfactory receptor | Olfactory 314 |
| Ol006 | Q8NH49 | Olfactory receptor | Olfactory 25 |
| Ol006 | Q8NGF9 | Olfactory receptor | Olfactory 25 |
| Ol006 | Q60881 | Olfactory receptor | Olfactory 219 |
| Ol006 | Q8NGF8 | Olfactory receptor | Olfactory 219 |
| Ol006 | Q8NGB4 | Olfactory receptor | Olfactory 314 |
| Ol006 | Q8NGL7 | Olfactory receptor | Olfactory 314 |
| Ol007 | Q9TU88 | Olfactory receptor | Olfactory II fam 2 / MOR256-262,270-285 |
| Ol007 | Q9TUA0 | Olfactory receptor | Olfactory II fam 2 / MOR256-262,270-285 |
| Ol007 | P47883 | Olfactory receptor | Olfactory II fam 2 / MOR256-262,270-285 |
| Ol007 | P47893 | Olfactory receptor | Olfactory II fam 2 / MOR256-262,270-285 |
| Ol007 | P47888 | Olfactory receptor | Olfactory II fam 2 / MOR256-262,270-285 |
| Ol007 | Q9TU97 | Olfactory receptor | Olfactory II fam 2 / MOR256-262,270-285 |
| Ol007 | Q60891 | Olfactory receptor | Olfactory II fam 2 / MOR256-262,270-285 |
| Ol008 | Q8NGK1 | Olfactory receptor | Olfactory 247 |
| Ol008 | Q8NGK0 | Olfactory receptor | Olfactory II fam 10 / MOR263-269 |
| Ol008 | Q8NGJ5 | Olfactory receptor | Olfactory 247 |
| Ol008 | Q9H341 | Olfactory receptor | Olfactory 133 |
| Ol008 | Q8NH63 | Olfactory receptor | Olfactory 242 |
| Ol008 | Q8NH59 | Olfactory receptor | Olfactory 247 |
| Ol009 | Q8NGD5 | Olfactory receptor | Olfactory II fam 8 / MOR161-171 |
| Ol009 | Q8NGC6 | Olfactory receptor | Olfactory II fam 8 / MOR161-171 |
| Ol009 | Q96R72 | Olfactory receptor | Olfactory II fam 8 / MOR161-171 |
| Ol009 | Q8NH43 | Olfactory receptor | Olfactory 118 |
| Ol009 | Q8NGD2 | Olfactory receptor | Olfactory II fam 8 / MOR161-171 |
| Ol009 | Q8NGD4 | Olfactory receptor | Olfactory II fam 8 / MOR161-171 |
| Ol010 | Q8NGP6 | Olfactory receptor | Olfactory 225 |
| Ol010 | Q8NGE7 | Olfactory receptor | Olfactory 290 |
| Ol010 | Q96RB7 | Olfactory receptor | Olfactory 60 |
| Ol010 | Q8VFL5 | Olfactory receptor | Olfactory 226 |
| Ol010 | Q8NGP3 | Olfactory receptor | Olfactory 225 |
| Ol010 | Q8NGP4 | Olfactory receptor | Olfactory 225 |
| Ol011 | Q8NG78 | Olfactory receptor | Olfactory 147 |
| Ol011 | Q15617 | Olfactory receptor | Olfactory 147 |
| Ol011 | Q15614 | Olfactory receptor | Olfactory 312 |
| Ol011 | Q60895 | Olfactory receptor | Olfactory 307 |
| Ol011 | P34983 | Olfactory receptor | Olfactory 147 |
| Ol011 | Q60884 | Olfactory receptor | Olfactory 147 |
| Ol012 | O60431 | Olfactory receptor | Olfactory 71 |
| Ol012 | Q8NH06 | Olfactory receptor | Olfactory 145 |
| Ol012 | Q8NGR9 | Olfactory receptor | Olfactory 134 |
| Ol012 | Q8VFM9 | Olfactory receptor | Olfactory 71 |
| Ol012 | Q8NGA1 | Olfactory receptor | Olfactory 71 |
| Ol012 | Q8NGS0 | Olfactory receptor | Olfactory 134 |
| Ol013 | Q8NGE5 | Olfactory receptor | Olfactory 284 |
| Ol013 | Q8NH19 | Olfactory receptor | Olfactory 51 |
| Ol013 | Q9H207 | Olfactory receptor | Olfactory 125 |
| Ol013 | Q9H208 | Olfactory receptor | Olfactory 125 |
| Ol013 | Q96KK4 | Olfactory receptor | Olfactory 149 |
| Ol013 | Q9H209 | Olfactory receptor | Olfactory 300 |
| Ol014 | Q8NG81 | Olfactory receptor | Olfactory 136 |
| Ol014 | A3KFT3 | Olfactory receptor | Olfactory 136 |
| Ol014 | Q8NHA4 | Olfactory receptor | Olfactory 136 |
| Ol014 | Q8NG83 | Olfactory receptor | Olfactory 136 |
| Ol014 | Q96R27 | Olfactory receptor | Olfactory 136 |
| Ol014 | Q96R28 | Olfactory receptor | Olfactory 136 |
| Ol015 | P37072 | Olfactory receptor | Olfactory II fam 5 / MOR172-224,249,254 |
| Ol015 | P37067 | Olfactory receptor | Olfactory II fam 5 / MOR172-224,249,254 |
| Ol015 | P37069 | Olfactory receptor | Olfactory II fam 5 / MOR172-224,249,254 |
| Ol015 | P37071 | Olfactory receptor | Olfactory II fam 5 / MOR172-224,249,254 |
| Ol015 | P37070 | Olfactory receptor | Olfactory II fam 5 / MOR172-224,249,254 |
| Ol015 | P37068 | Olfactory receptor | Olfactory II fam 5 / MOR172-224,249,254 |
| Ol016 | Q8NGC9 | Olfactory receptor | Olfactory 206 |
| Ol016 | Q8NGX0 | Olfactory receptor | Olfactory 316 |
| Ol016 | Q8NGC1 | Olfactory receptor | Olfactory 117 |
| Ol016 | Q8NGC7 | Olfactory receptor | Olfactory 235 |
| Ol016 | Q8NGC8 | Olfactory receptor | Olfactory 206 |
| Ol017 | Q9Y5P0 | Olfactory receptor | Olfactory 244 |
| Ol017 | Q9H2C8 | Olfactory receptor | Olfactory 247 |
| Ol017 | Q9Y5P1 | Olfactory receptor | Olfactory 244 |
| Ol017 | Q9H339 | Olfactory receptor | Olfactory 244 |
| Ol017 | Q9H340 | Olfactory receptor | Olfactory 244 |
| Ol018 | Q8NH56 | Olfactory receptor | Olfactory II fam 12 / MOR250 |
| Ol018 | Q8NGF1 | Olfactory receptor | Olfactory II fam 12 / MOR250 |
| Ol018 | Q8NH53 | Olfactory receptor | Olfactory II fam 12 / MOR250 |
| Ol018 | Q8NGI2 | Olfactory receptor | Olfactory II fam 12 / MOR250 |
| Ol018 | Q8NGI0 | Olfactory receptor | Olfactory II fam 12 / MOR250 |
| Ol019 | O43749 | Olfactory receptor | Olfactory 134 |
| Ol019 | Q15619 | Olfactory receptor | Olfactory 71 |
| Ol019 | Q96R84 | Olfactory receptor | Olfactory 134 |
| Ol019 | Q8NHA8 | Olfactory receptor | Olfactory 71 |
| Ol019 | P23266 | Olfactory receptor | Olfactory 134 |
| Ol020 | Q96R47 | Olfactory receptor | Olfactory II fam 3 / MOR255 |
| Ol020 | Q6IF42 | Olfactory receptor | Olfactory II fam 3 / MOR255 |
| Ol020 | Q8NGT9 | Olfactory receptor | Olfactory II fam 3 / MOR255 |
| Ol020 | Q96R48 | Olfactory receptor | Olfactory II fam 3 / MOR255 |
| Ol020 | Q8NGT7 | Olfactory receptor | Olfactory II fam 3 / MOR255 |
| Ol021 | Q8NGM1 | Olfactory receptor | Olfactory 25 |
| Ol021 | A6NMZ5 | Olfactory receptor | Olfactory 25 |
| Ol021 | A6NHA9 | Olfactory receptor | Olfactory 90 |
| Ol021 | Q96R67 | Olfactory receptor | Olfactory 220 |
| Ol021 | Q8NGP0 | Olfactory receptor | Olfactory 25 |
| Ol022 | Q8NGI4 | Olfactory receptor | Olfactory 19 |
| Ol022 | Q8NGE8 | Olfactory receptor | Olfactory 19 |
| Ol022 | Q8NGJ1 | Olfactory receptor | Olfactory 92 |
| Ol022 | Q8NGN0 | Olfactory receptor | Olfactory 297 |
| Ol022 | Q8NGI6 | Olfactory receptor | Olfactory 19 |
| Ol023 | Q9GZK3 | Olfactory receptor | Olfactory 190 |
| Ol023 | O76000 | Olfactory receptor | Olfactory 192 |
| Ol023 | Q5JQS5 | Olfactory receptor | Olfactory 204 |
| Ol023 | Q60890 | Olfactory receptor | Olfactory 105 |
| Ol023 | P58173 | Olfactory receptor | Olfactory 105 |
| Ol024 | Q8NH72 | Olfactory receptor | Olfactory 45 |
| Ol024 | Q8NGL6 | Olfactory receptor | Olfactory 25 |
| Ol024 | Q8NGB2 | Olfactory receptor | Olfactory 25 |
| Ol024 | Q6IF82 | Olfactory receptor | Olfactory 25 |
| Ol024 | Q8NGN8 | Olfactory receptor | Olfactory 288 |
| Ol025 | P35896 | Olfactory receptor | Olfactory 70 |
| Ol025 | Q96RA2 | Olfactory receptor | Olfactory 71 |
| Ol025 | P34987 | Olfactory receptor | Olfactory 70 |
| Ol025 | Q8NG98 | Olfactory receptor | Olfactory 71 |
| Ol025 | Q7TRF3 | Olfactory receptor | Olfactory 70 |
| Ol026 | Q8NGQ2 | Olfactory receptor | Olfactory 103 |
| Ol026 | P34986 | Olfactory receptor | Olfactory 138 |
| Ol026 | O95007 | Olfactory receptor | Olfactory 156 |
| Ol026 | Q8NGW1 | Olfactory receptor | Olfactory 103 |
| Ol026 | Q6IFH4 | Olfactory receptor | Olfactory 103 |
| Ol027 | Q8VFK7 | Olfactory receptor | Olfactory 226 |
| Ol027 | Q9UGF6 | Olfactory receptor | Olfactory 234 |
| Ol027 | Q8NGF4 | Olfactory receptor | Olfactory 226 |
| Ol027 | Q8NGC0 | Olfactory receptor | Olfactory 211 |
| Ol027 | O95221 | Olfactory receptor | Olfactory 193 |
| Ol028 | Q8NGX9 | Olfactory receptor | Olfactory 252 |
| Ol028 | Q60889 | Olfactory receptor | Olfactory 109 |
| Ol028 | Q8NGX8 | Olfactory receptor | Olfactory 187 |
| Ol028 | O95222 | Olfactory receptor | Olfactory 156 |
| Ol028 | P23270 | Olfactory receptor | Olfactory 156 |
| Ol029 | Q8NGT0 | Olfactory receptor | Olfactory 122 |
| Ol029 | Q8NGT2 | Olfactory receptor | Olfactory 263 |
| Ol029 | Q8NGS8 | Olfactory receptor | Olfactory 122 |
| Ol029 | Q8NGV5 | Olfactory receptor | Olfactory 37 |
| Ol029 | Q8NGS9 | Olfactory receptor | Olfactory 122 |
| Ol030 | Q8NGN2 | Olfactory receptor | Olfactory 269 |
| Ol030 | Q8NGN6 | Olfactory receptor | Olfactory II fam 6 / MOR103-105,107-119 |
| Ol030 | Q8NGN4 | Olfactory receptor | Olfactory II fam 6 / MOR103-105,107-119 |
| Ol030 | Q8NGN5 | Olfactory receptor | Olfactory II fam 6 / MOR103-105,107-119 |
| Ol030 | Q8NGN3 | Olfactory receptor | Olfactory II fam 6 / MOR103-105,107-119 |
| Ol031 | Q8VGS3 | Olfactory receptor | Olfactory 28 |
| Ol031 | Q8NGR4 | Olfactory receptor | Olfactory 39 |
| Ol031 | Q60894 | Olfactory receptor | Olfactory 291 |
| Ol031 | Q8NGP9 | Olfactory receptor | Olfactory 28 |
| Ol031 | Q8N127 | Olfactory receptor | Olfactory 54 |
| Ol032 | O88628 | Olfactory receptor | Olfactory 174 |
| Ol032 | Q8TCB6 | Olfactory receptor | Olfactory 75 |
| Ol032 | Q8VBV9 | Olfactory receptor | Olfactory 174 |
| Ol032 | Q9H255 | Olfactory receptor | Olfactory 174 |
| Ol032 | Q8NGF3 | Olfactory receptor | Olfactory 248 |
| Ol033 | Q8NH80 | Olfactory receptor | Olfactory II fam 6 / MOR103-105,107-119 |
| Ol033 | Q60888 | Olfactory receptor | Olfactory II fam 6 / MOR103-105,107-119 |
| Ol033 | Q60887 | Olfactory receptor | Olfactory II fam 6 / MOR103-105,107-119 |
| Ol033 | Q8NGN7 | Olfactory receptor | Olfactory II fam 6 / MOR103-105,107-119 |
| Ol033 | Q8VEY3 | Olfactory receptor | Olfactory II fam 6 / MOR103-105,107-119 |
| Ol034 | Q9TUA6 | Olfactory receptor | Olfactory 78 |
| Ol034 | P58170 | Olfactory receptor | Olfactory 78 |
| Ol034 | P47884 | Olfactory receptor | Olfactory 78 |
| Ol034 | Q9TU90 | Olfactory receptor | Olfactory 78 |
| Ol034 | Q9TU95 | Olfactory receptor | Olfactory 78 |
| Ol035 | Q8VG03 | Olfactory receptor | Olfactory II fam 4 / MOR225-248 |
| Ol035 | Q8VFD3 | Olfactory receptor | Olfactory II fam 4 / MOR225-248 |
| Ol035 | Q8VEW5 | Olfactory receptor | Olfactory II fam 4 / MOR225-248 |
| Ol035 | Q8VFD1 | Olfactory receptor | Olfactory II fam 4 / MOR225-248 |
| Ol035 | Q8VFD2 | Olfactory receptor | Olfactory II fam 4 / MOR225-248 |
| Ol036 | Q8NGM8 | Olfactory receptor | Olfactory 129 |
| Ol036 | Q8NH40 | Olfactory receptor | Olfactory 177 |
| Ol036 | Q8NH79 | Olfactory receptor | Olfactory 129 |
| Ol036 | Q8NGC5 | Olfactory receptor | Olfactory 129 |
| Ol037 | Q96RD2 | Olfactory receptor | Olfactory 241 |
| Ol037 | Q8NGJ2 | Olfactory receptor | Olfactory 304 |
| Ol037 | Q8NGK2 | Olfactory receptor | Olfactory 91 |
| Ol037 | Q8NGF0 | Olfactory receptor | Olfactory 241 |
| Ol038 | Q8NGZ5 | Olfactory receptor | Olfactory 20 |
| Ol038 | Q8N628 | Olfactory receptor | Olfactory 194 |
| Ol038 | Q5TZ20 | Olfactory receptor | Olfactory 106 |
| Ol038 | Q8NGZ4 | Olfactory receptor | Olfactory 106 |
| Ol039 | Q8NGY1 | Olfactory receptor | Olfactory 215 |
| Ol039 | P30954 | Olfactory receptor | Olfactory 215 |
| Ol039 | Q8NHC4 | Olfactory receptor | Olfactory 215 |
| Ol039 | Q8NGY7 | Olfactory receptor | Olfactory 215 |
| Ol040 | Q8NGL1 | Olfactory receptor | Olfactory 26 |
| Ol040 | Q8NGL4 | Olfactory receptor | Olfactory 287 |
| Ol040 | Q8NGL3 | Olfactory receptor | Olfactory 47 |
| Ol040 | Q8NGK9 | Olfactory receptor | Olfactory 47 |
| Ol041 | A6NJZ3 | Olfactory receptor | Olfactory 320 |
| Ol041 | A6NIJ9 | Olfactory receptor | Olfactory 77 |
| Ol041 | A6NF89 | Olfactory receptor | Olfactory 77 |
| Ol041 | A6NM76 | Olfactory receptor | Olfactory 320 |
| Ol042 | Q8NH48 | Olfactory receptor | Olfactory 226 |
| Ol042 | Q96R08 | Olfactory receptor | Olfactory 226 |
| Ol042 | Q96R09 | Olfactory receptor | Olfactory 226 |
| Ol042 | Q8NGF7 | Olfactory receptor | Olfactory 226 |
| Ol043 | Q96RD1 | Olfactory receptor | Olfactory 229 |
| Ol043 | A6NL08 | Olfactory receptor | Olfactory 320 |
| Ol043 | A6NCV1 | Olfactory receptor | Olfactory 320 |
| Ol043 | Q9NZP0 | Olfactory receptor | Olfactory 320 |
| Ol044 | Q8NH94 | Olfactory receptor | Olfactory 257 |
| Ol044 | Q8NH93 | Olfactory receptor | Olfactory 257 |
| Ol044 | Q15612 | Olfactory receptor | Olfactory 258 |
| Ol044 | Q8NGR8 | Olfactory receptor | Olfactory 259 |
| Ol045 | Q8NGE2 | Olfactory receptor | Olfactory 272 |
| Ol045 | Q8NGE1 | Olfactory receptor | Olfactory 272 |
| Ol045 | Q9Z1V0 | Olfactory receptor | Olfactory 216 |
| Ol045 | Q8NGN1 | Olfactory receptor | Olfactory 216 |
| Ol046 | P59922 | Olfactory receptor | Olfactory 106 |
| Ol046 | Q60883 | Olfactory receptor | Olfactory 106 |
| Ol046 | Q8NGV0 | Olfactory receptor | Olfactory 106 |
| Ol046 | Q8NGU4 | Olfactory receptor | Olfactory 295 |
| Ol047 | Q15620 | Olfactory receptor | Olfactory 68 |
| Ol047 | Q8NGG6 | Olfactory receptor | Olfactory 68 |
| Ol047 | Q60882 | Olfactory receptor | Olfactory 68 |
| Ol047 | P34985 | Olfactory receptor | Olfactory 228 |
| Ol048 | Q8NH54 | Olfactory receptor | Olfactory 15 |
| Ol048 | Q8NGH8 | Olfactory receptor | Olfactory 15 |
| Ol048 | P0C7T3 | Olfactory receptor | Olfactory 15 |
| Ol048 | Q8NGH5 | Olfactory receptor | Olfactory 15 |
| Ol049 | Q60880 | Olfactory receptor | Olfactory 18 |
| Ol049 | Q8VES2 | Olfactory receptor | Olfactory 18 |
| Ol049 | Q8VF13 | Olfactory receptor | Olfactory 18 |
| Ol049 | Q8VFL9 | Olfactory receptor | Olfactory 18 |
| Ol050 | Q8VEZ0 | Olfactory receptor | Olfactory II fam 4 / MOR225-248 |
| Ol050 | Q8WZ94 | Olfactory receptor | Olfactory II fam 4 / MOR225-248 |
| Ol050 | Q8VG42 | Olfactory receptor | Olfactory II fam 4 / MOR225-248 |
| Ol050 | Q8VGI5 | Olfactory receptor | Olfactory 202 |
| Ol051 | Q8VFC9 | Olfactory receptor | Olfactory 180 |
| Ol051 | Q8VG44 | Olfactory receptor | Olfactory 180 |
| Ol051 | Q8VG06 | Olfactory receptor | Olfactory II fam 4 / MOR225-248 |
| Ol051 | Q8VG43 | Olfactory receptor | Olfactory 180 |
| Ol052 | Q7TR96 | Olfactory receptor | Olfactory 267 |
| Ol052 | Q8NGQ1 | Olfactory receptor | Olfactory 29 |
| Ol052 | P0C7N8 | Olfactory receptor | Olfactory 313 |
| Ol052 | Q8NH87 | Olfactory receptor | Olfactory 313 |
| Ol053 | Q8NGB8 | Olfactory receptor | Olfactory II fam 7 / MOR139-155 |
| Ol053 | Q8NGB9 | Olfactory receptor | Olfactory 283 |
| Ol053 | Q6IEY1 | Olfactory receptor | Olfactory 218 |
| Ol053 | O95013 | Olfactory receptor | Olfactory 218 |
| Ol054 | Q8N349 | Olfactory receptor | Olfactory 16 |
| Ol054 | Q8NG80 | Olfactory receptor | Olfactory 16 |
| Ol054 | Q8NH16 | Olfactory receptor | Olfactory 16 |
| Ol054 | Q8NGZ0 | Olfactory receptor | Olfactory 16 |
| Ol055 | P34984 | Olfactory receptor | Olfactory II fam 3 / MOR255 |
| Ol055 | Q96R45 | Olfactory receptor | Olfactory II fam 3 / MOR255 |
| Ol055 | O95047 | Olfactory receptor | Olfactory II fam 3 / MOR255 |
| Ol055 | A4D2G3 | Olfactory receptor | Olfactory II fam 3 / MOR255 |
| Ol056 | Q96RC9 | Olfactory receptor | Olfactory 64 |
| Ol056 | Q60886 | Olfactory receptor | Olfactory 64 |
| Ol056 | Q8NGG8 | Olfactory receptor | Olfactory 64 |
| Ol056 | Q96RD0 | Olfactory receptor | Olfactory 64 |
| Ol057 | P0C623 | Olfactory receptor | Olfactory 297 |
| Ol057 | Q8NGB6 | Olfactory receptor | Olfactory 297 |
| Ol057 | Q8NH05 | Olfactory receptor | Olfactory 297 |
| Ol057 | Q8NGD0 | Olfactory receptor | Olfactory 297 |
| Ol058 | Q8VGI1 | Olfactory receptor | Olfactory 71 |
| Ol058 | P23274 | Olfactory receptor | Olfactory 71 |
| Ol058 | P70526 | Olfactory receptor | Olfactory 71 |
| Ol058 | P23272 | Olfactory receptor | Olfactory 71 |
| Ol059 | Q8NH10 | Olfactory receptor | Olfactory 152 |
| Ol059 | P0C7N5 | Olfactory receptor | Olfactory 90 |
| Ol059 | P0C7N1 | Olfactory receptor | Olfactory 152 |
| Ol059 | Q8VGR9 | Olfactory receptor | Olfactory 152 |
| Ol060 | Q8NH02 | Olfactory receptor | Olfactory II fam 13 / MOR253 |
| Ol060 | Q6IEZ7 | Olfactory receptor | Olfactory II fam 13 / MOR253 |
| Ol060 | Q8NGZ9 | Olfactory receptor | Olfactory II fam 13 / MOR253 |
| Ol060 | Q8NH00 | Olfactory receptor | Olfactory II fam 13 / MOR253 |
| Ol061 | Q8NH04 | Olfactory receptor | Olfactory 34 |
| Ol061 | P0C7T2 | Olfactory receptor | Olfactory 34 |
| Ol061 | Q8NHC8 | Olfactory receptor | Olfactory 35 |
| Ol061 | O43869 | Olfactory receptor | Olfactory 35 |
| Ol062 | A6NH00 | Olfactory receptor | Olfactory 38 |
| Ol062 | Q8NG76 | Olfactory receptor | Olfactory 38 |
| Ol062 | Q8NG97 | Olfactory receptor | Olfactory 23 |
| Ol062 | Q8NG77 | Olfactory receptor | Olfactory 38 |
| Ol063 | Q9TU92 | Olfactory receptor | Olfactory 319 |
| Ol063 | Q9TUA7 | Olfactory receptor | Olfactory 319 |
| Ol063 | Q9Y585 | Olfactory receptor | Olfactory 319 |
| Ol063 | Q9P1Q5 | Olfactory receptor | Olfactory 319 |
| Ol064 | Q8N162 | Olfactory receptor | Olfactory 82 |
| Ol064 | Q8N0Y5 | Olfactory receptor | Olfactory 82 |
| Ol064 | Q8NGG4 | Olfactory receptor | Olfactory 82 |
| Ol064 | Q8N146 | Olfactory receptor | Olfactory 82 |
| Ol065 | Q8NGF6 | Olfactory receptor | Olfactory II fam 3 / MOR255 |
| Ol065 | Q8NGI7 | Olfactory receptor | Olfactory II fam 3 / MOR255 |
| Ol065 | Q8NGQ4 | Olfactory receptor | Olfactory 227 |
| Ol066 | Q8NGY0 | Olfactory receptor | Olfactory 210 |
| Ol066 | Q8NGX3 | Olfactory receptor | Olfactory 210 |
| Ol066 | Q8NGX6 | Olfactory receptor | Olfactory 214 |
| Ol067 | A6NGY5 | Olfactory receptor | Olfactory 172 |
| Ol067 | Q8NGJ9 | Olfactory receptor | Olfactory 21 |
| Ol067 | Q8NH61 | Olfactory receptor | Olfactory 247 |
| Ol068 | Q8NGY2 | Olfactory receptor | Olfactory 251 |
| Ol068 | Q8NGW6 | Olfactory receptor | Olfactory 251 |
| Ol068 | Q8NGY3 | Olfactory receptor | Olfactory 251 |
| Ol069 | Q7Z3T1 | Olfactory receptor | Olfactory 73 |
| Ol069 | Q9Y3N9 | Olfactory receptor | Olfactory 189 |
| Ol069 | Q8NHA6 | Olfactory receptor | Olfactory 73 |
| Ol070 | Q8NH50 | Olfactory receptor | Olfactory 59 |
| Ol070 | Q8NH51 | Olfactory receptor | Olfactory 57 |
| Ol070 | Q8NGG5 | Olfactory receptor | Olfactory 113 |
| Ol071 | Q8NH76 | Olfactory receptor | Olfactory 158 |
| Ol071 | Q8NGI3 | Olfactory receptor | Olfactory 88 |
| Ol071 | Q8NGI1 | Olfactory receptor | Olfactory 88 |
| Ol072 | Q8NGD3 | Olfactory receptor | Olfactory II fam 8 / MOR161-171 |
| Ol072 | Q8NH42 | Olfactory receptor | Olfactory II fam 8 / MOR161-171 |
| Ol072 | Q8NH41 | Olfactory receptor | Olfactory II fam 8 / MOR161-171 |
| Ol073 | Q8NGC4 | Olfactory receptor | Olfactory 269 |
| Ol073 | Q8NGC3 | Olfactory receptor | Olfactory 269 |
| Ol073 | Q8NH81 | Olfactory receptor | Olfactory 269 |
| Ol074 | Q8NG95 | Olfactory receptor | Olfactory 71 |
| Ol074 | Q8NGA0 | Olfactory receptor | Olfactory 71 |
| Ol074 | Q8NG99 | Olfactory receptor | Olfactory 71 |
| Ol075 | Q8NGQ5 | Olfactory receptor | Olfactory 18 |
| Ol075 | Q8NGE9 | Olfactory receptor | Olfactory 18 |
| Ol075 | Q8NGQ6 | Olfactory receptor | Olfactory 18 |
| Ol076 | Q8NH70 | Olfactory receptor | Olfactory 288 |
| Ol076 | P0C604 | Olfactory receptor | Olfactory 288 |
| Ol076 | Q8NH83 | Olfactory receptor | Olfactory 288 |
| Ol077 | Q9UKL2 | Olfactory receptor | Olfactory 120 |
| Ol077 | A6NMU1 | Olfactory receptor | Olfactory 120 |
| Ol077 | Q9H2C5 | Olfactory receptor | Olfactory 200 |
| Ol078 | Q8NGS1 | Olfactory receptor | Olfactory 71 |
| Ol078 | Q8VGK5 | Olfactory receptor | Olfactory 87 |
| Ol078 | Q8NGS2 | Olfactory receptor | Olfactory 71 |
| Ol079 | Q8NGT5 | Olfactory receptor | Olfactory 129 |
| Ol079 | Q8NGU2 | Olfactory receptor | Olfactory 129 |
| Ol079 | Q8NGU1 | Olfactory receptor | Olfactory 129 |
| Ol080 | Q8NGG7 | Olfactory receptor | Olfactory 226 |
| Ol080 | Q8NGM9 | Olfactory receptor | Olfactory 226 |
| Ol080 | Q60893 | Olfactory receptor | Olfactory 226 |
| Ol081 | Q8NGG3 | Olfactory receptor | Olfactory 18 |
| Ol081 | Q8NGG2 | Olfactory receptor | Olfactory 18 |
| Ol081 | Q8NG75 | Olfactory receptor | Olfactory 18 |
| Ol082 | P23271 | Olfactory receptor | Olfactory 71 |
| Ol082 | P23273 | Olfactory receptor | Olfactory 71 |
| Ol082 | P23269 | Olfactory receptor | Olfactory 71 |
| Ol083 | Q8IXE1 | Olfactory receptor | Olfactory 297 |
| Ol083 | Q8N0Y3 | Olfactory receptor | Olfactory 297 |
| Ol083 | Q8NGD1 | Olfactory receptor | Olfactory 297 |
| Ol084 | O60404 | Olfactory receptor | Olfactory 37 |
| Ol084 | Q8VBW9 | Olfactory receptor | Olfactory 37 |
| Ol084 | Q8NGA5 | Olfactory receptor | Olfactory 37 |
| Ol085 | Q8NGA6 | Olfactory receptor | Olfactory 37 |
| Ol085 | Q9Y4A9 | Olfactory receptor | Olfactory 37 |
| Ol085 | O60403 | Olfactory receptor | Olfactory 37 |
| Ol086 | Q8VG09 | Olfactory receptor | Olfactory II fam 4 / MOR225-248 |
| Ol086 | Q8VG04 | Olfactory receptor | Olfactory II fam 4 / MOR225-248 |
| Ol086 | Q8WZ92 | Olfactory receptor | Olfactory II fam 4 / MOR225-248 |
| Ol087 | Q8NGP2 | Olfactory receptor | Olfactory 27 |
| Ol087 | Q8NGG0 | Olfactory receptor | Olfactory 27 |
| Ol087 | Q8NGG1 | Olfactory receptor | Olfactory 27 |
| Ol088 | Q8NGK3 | Olfactory receptor | Olfactory 22 |
| Ol088 | Q8NGK5 | Olfactory receptor | Olfactory 89 |
| Ol088 | Q8NGK4 | Olfactory receptor | Olfactory 22 |
| Ol089 | Q9QY00 | Olfactory receptor | Olfactory 61 |
| Ol089 | Q8VFK2 | Olfactory receptor | Olfactory 61 |
| Ol089 | Q8VFK1 | Olfactory receptor | Olfactory 268 |
| Ol090 | A6NET4 | Olfactory receptor | Olfactory 142 |
| Ol090 | Q8NHB7 | Olfactory receptor | Olfactory 188 |
| Ol090 | Q8NHB8 | Olfactory receptor | Olfactory 188 |
| Ol091 | Q13607 | Olfactory receptor | Olfactory 31 |
| Ol091 | Q95156 | Olfactory receptor | Olfactory 31 |
| Ol091 | O95006 | Olfactory receptor | Olfactory 31 |
| Ol092 | O76001 | Olfactory receptor | Olfactory 191 |
| Ol092 | O76002 | Olfactory receptor | Olfactory 191 |
| Ol092 | Q9GZK6 | Olfactory receptor | Olfactory 191 |
| Ol093 | B2RN74 | Olfactory receptor | Olfactory 235 |
| Ol093 | Q8NG94 | Olfactory receptor | Olfactory 235 |
| Ol093 | Q8NH07 | Olfactory receptor | Olfactory 235 |
| Ol094 | Q8NGA8 | Olfactory receptor | Olfactory II fam 8 / MOR161-171 |
| Ol094 | Q8NH21 | Olfactory receptor | Olfactory II fam 8 / MOR161-171 |
| Ol094 | Q96R69 | Olfactory receptor | Olfactory II fam 8 / MOR161-171 |
| Ol095 | Q96R30 | Olfactory receptor | Olfactory 38 |
| Ol095 | Q8NHB1 | Olfactory receptor | Olfactory 38 |
| Ol095 | Q8VGD6 | Olfactory receptor | Olfactory 38 |
| Ol096 | Q8NH64 | Olfactory receptor | Olfactory 209 |
| Ol096 | Q8NGJ6 | Olfactory receptor | Olfactory 209 |
| Ol096 | Q8NGJ7 | Olfactory receptor | Olfactory 209 |
| Ol097 | Q7TS48 | Olfactory receptor | Olfactory 280 |
| Ol097 | A6NMS3 | Olfactory receptor | Olfactory 80 |
| Ol097 | Q8VGQ7 | Olfactory receptor | Olfactory 280 |
| Ol098 | Q8NH85 | Olfactory receptor | Olfactory 224 |
| Ol098 | P0C617 | Olfactory receptor | Olfactory 224 |
| Ol098 | Q8VGS1 | Olfactory receptor | Olfactory 224 |
| Ol099 | Q9TU93 | Olfactory receptor | Olfactory 317 |
| Ol099 | Q9TUA8 | Olfactory receptor | Olfactory 317 |
| Ol099 | P34982 | Olfactory receptor | Olfactory 317 |
| Ol100 | Q9TUA4 | Olfactory receptor | Olfactory II fam 2 / MOR256-262,270-285 |
| Ol100 | Q9TU89 | Olfactory receptor | Olfactory II fam 2 / MOR256-262,270-285 |
| Ol100 | P47881 | Olfactory receptor | Olfactory II fam 2 / MOR256-262,270-285 |
| Ol101 | Q6IF00 | Olfactory receptor | Olfactory 33 |
| Ol101 | Q8NH01 | Olfactory receptor | Olfactory 315 |
| Ol101 | Q8NGX2 | Olfactory receptor | Olfactory 33 |
| Ol102 | Q8NGR2 | Olfactory receptor | Olfactory 96 |
| Ol102 | Q8NGR5 | Olfactory receptor | Olfactory 96 |
| Ol102 | Q8NGR3 | Olfactory receptor | Olfactory 257 |
| Ol103 | Q8NGY9 | Olfactory receptor | Olfactory 16 |
| Ol103 | Q8NG84 | Olfactory receptor | Olfactory 301 |
| Ol103 | Q8NG85 | Olfactory receptor | Olfactory 16 |
| Ol104 | Q8VG08 | Olfactory receptor | Olfactory II fam 4 / MOR225-248 |
| Ol104 | Q8VG05 | Olfactory receptor | Olfactory II fam 4 / MOR225-248 |
| Ol104 | Q8VG07 | Olfactory receptor | Olfactory II fam 4 / MOR225-248 |
| Ol105 | Q8NGR1 | Olfactory receptor | Olfactory 298 |
| Ol105 | Q8NGZ3 | Olfactory receptor | Olfactory 277 |
| Ol106 | Q60885 | Olfactory receptor | Olfactory 154 |
| Ol106 | Q8NGE3 | Olfactory receptor | Olfactory 154 |
| Ol107 | Q8NGS4 | Olfactory receptor | Olfactory 37 |
| Ol107 | Q8NGT1 | Olfactory receptor | Olfactory 37 |
| Ol108 | Q8NGC2 | Olfactory receptor | Olfactory 212 |
| Ol108 | P0C645 | Olfactory receptor | Olfactory 212 |
| Ol109 | Q9H210 | Olfactory receptor | Olfactory 238 |
| Ol109 | Q8NGH3 | Olfactory receptor | Olfactory 119 |
| Ol110 | Q9H343 | Olfactory receptor | Olfactory 243 |
| Ol110 | Q9H344 | Olfactory receptor | Olfactory 242 |
| Ol111 | Q8NGY5 | Olfactory receptor | Olfactory 251 |
| Ol111 | Q8NGY6 | Olfactory receptor | Olfactory 251 |
| Ol112 | Q8NGI9 | Olfactory receptor | Olfactory 18 |
| Ol112 | Q8NGJ0 | Olfactory receptor | Olfactory 18 |
| Ol113 | Q5JRS4 | Olfactory receptor | Olfactory 266 |
| Ol113 | P0C629 | Olfactory receptor | Olfactory 266 |
| Ol114 | Q8WZ84 | Olfactory receptor | Olfactory 63 |
| Ol114 | Q9GZM6 | Olfactory receptor | Olfactory 63 |
| Ol115 | P58182 | Olfactory receptor | Olfactory 281 |
| Ol115 | Q9UGF7 | Olfactory receptor | Olfactory 281 |
| Ol116 | P23267 | Olfactory receptor | Olfactory 177 |
| Ol116 | Q8NGZ6 | Olfactory receptor | Olfactory 262 |
| Ol117 | Q8NGI8 | Olfactory receptor | Olfactory 101 |
| Ol117 | Q8VFV4 | Olfactory receptor | Olfactory 101 |
| Ol118 | Q9NQN1 | Olfactory receptor | Olfactory 95 |
| Ol118 | Q8NGS7 | Olfactory receptor | Olfactory 36 |
| Ol119 | Q8NGL9 | Olfactory receptor | Olfactory 17 |
| Ol119 | Q6IEV9 | Olfactory receptor | Olfactory 17 |
| Ol120 | Q8NGJ4 | Olfactory receptor | Olfactory 246 |
| Ol120 | Q8NGJ3 | Olfactory receptor | Olfactory 246 |
| Ol121 | Q60878 | Olfactory receptor | Olfactory 25 |
| Ol121 | Q8NH37 | Olfactory receptor | Olfactory 25 |
| Ol122 | P0C628 | Olfactory receptor | Olfactory 79 |
| Ol122 | Q9NZP5 | Olfactory receptor | Olfactory 79 |
| Ol123 | Q6IFN5 | Olfactory receptor | Olfactory 24 |
| Ol123 | Q0VAX9 | Olfactory receptor | Olfactory 24 |
| Ol124 | Q8NGS3 | Olfactory receptor | Olfactory 71 |
| Ol124 | Q60879 | Olfactory receptor | Olfactory 71 |
| Ol125 | Q9NZP2 | Olfactory receptor | Olfactory 320 |
| Ol125 | A6NDL8 | Olfactory receptor | Olfactory 115 |
| Ol126 | Q8VGR8 | Olfactory receptor | Olfactory 58 |
| Ol126 | Q8NH18 | Olfactory receptor | Olfactory 58 |
| Ol127 | Q8NH89 | Olfactory receptor | Olfactory 226 |
| Ol127 | Q8NH90 | Olfactory receptor | Olfactory 226 |
| Ol128 | Q8NH69 | Olfactory receptor | Olfactory II fam 5 / MOR172-224,249,254 |
| Ol128 | Q95155 | Olfactory receptor | Olfactory II fam 5 / MOR172-224,249,254 |
| Ol129 | Q15615 | Olfactory receptor | Olfactory 182 |
| Ol129 | P58180 | Olfactory receptor | Olfactory 40 |
| Ol130 | O60412 | Olfactory receptor | Olfactory 217 |
| Ol130 | O76099 | Olfactory receptor | Olfactory 71 |
| Ol131 | A6NL26 | Olfactory receptor | Olfactory 226 |
| Ol131 | Q8VFX2 | Olfactory receptor | Olfactory 213 |
| Ol132 | Q8NGH7 | Olfactory receptor | Olfactory II fam 12 / MOR250 |
| Ol132 | Q8NGH6 | Olfactory receptor | Olfactory II fam 12 / MOR250 |
| Ol133 | P0C626 | Olfactory receptor | Olfactory 62 |
| Ol133 | Q8VF76 | Olfactory receptor | Olfactory 62 |
| Ol134 | Q8NGX5 | Olfactory receptor | Olfactory 215 |
| Ol134 | Q6IF99 | Olfactory receptor | Olfactory 215 |
| Ol135 | Q8VFD0 | Olfactory receptor | Olfactory II fam 4 / MOR225-248 |
| Ol135 | Q8VG02 | Olfactory receptor | Olfactory II fam 4 / MOR225-248 |
| Ol136 | O95371 | Olfactory receptor | Olfactory 159 |
| Ol136 | P23275 | Olfactory receptor | Olfactory 159 |
| Ol137 | Q8VEW6 | Olfactory receptor | Olfactory II fam 4 / MOR225-248 |
| Ol137 | Q8VG13 | Olfactory receptor | Olfactory II fam 4 / MOR225-248 |
| Ol138 | P58181 | Olfactory receptor | Olfactory 178 |
| Ol138 | Q8NH74 | Olfactory receptor | Olfactory 178 |
| Ol139 | Q95154 | Olfactory receptor | Olfactory 49 |
| Ol139 | Q13606 | Olfactory receptor | Olfactory 49 |
| Ol140 | Q9GZK4 | Olfactory receptor | Olfactory 20 |
| Ol140 | O95918 | Olfactory receptor | Olfactory 20 |
| Ol141 | Q9H205 | Olfactory receptor | Olfactory 14 |
| Ol141 | A6NM03 | Olfactory receptor | Olfactory 14 |
| Ol142 | Q8NGS5 | Olfactory receptor | Olfactory 36 |
| Ol142 | Q8NGS6 | Olfactory receptor | Olfactory 36 |
| Ol143 | Q8VGI6 | Olfactory receptor | Olfactory II fam 4 / MOR225-248 |
| Ol143 | Q8VGI4 | Olfactory receptor | Olfactory II fam 4 / MOR225-248 |
| Ol144 | Q8VF66 | Olfactory receptor | Olfactory II fam 4 / MOR225-248 |
| Ol144 | Q8VF65 | Olfactory receptor | Olfactory II fam 4 / MOR225-248 |
| Ol145 | Q8NH67 | Olfactory receptor | Olfactory 285 |
| Ol145 | Q8NGK6 | Olfactory receptor | Olfactory 285 |
| Ol146 | Q8VEW2 | Olfactory receptor | Olfactory II fam 4 / MOR225-248 |
| Ol146 | Q8VF12 | Olfactory receptor | Olfactory II fam 4 / MOR225-248 |
| Ol147 | P47890 | Olfactory receptor | Olfactory 134 |
| Ol147 | Q9TU86 | Olfactory receptor | Olfactory 71 |
| Ol148 | Q8NGL0 | Olfactory receptor | Olfactory 286 |
| Ol148 | Q8NGL2 | Olfactory receptor | Olfactory 286 |
| Ol149 | Q8NH03 | Olfactory receptor | Olfactory II fam 13 / MOR253 |
| Ol149 | Q8NGX1 | Olfactory receptor | Olfactory II fam 13 / MOR253 |
| Ol150 | Q8NGQ3 | Olfactory receptor | Olfactory 71 |
| Ol150 | Q8NH92 | Olfactory receptor | Olfactory 71 |
| Ol151 | Q6IEU7 | Olfactory receptor | Olfactory 60 |
| Ol151 | Q8NGP8 | Olfactory receptor | Olfactory 60 |
| OtR001 | A2ARI4 | Class A Orphan/other | LGR like (hormone receptors) type 4 and 5 |
| OtR001 | O75473 | Class A Orphan/other | LGR like (hormone receptors) type 4 and 5 |
| OtR001 | Q9HBX8 | Class A Orphan/other | LGR like (hormone receptors) type 4 and 5 |
| OtR001 | B0BLW3 | Class A Orphan/other | LGR like (hormone receptors) type 4 and 5 |
| OtR001 | Q3UVD5 | Class A Orphan/other | LGR like (hormone receptors) type 4 and 5 |
| OtR001 | D4AC13 | Class A Orphan/other | LGR like (hormone receptors) type 4 and 5 |
| OtR001 | Q9Z1P4 | Class A Orphan/other | LGR like (hormone receptors) type 4 and 5 |
| OtR001 | Q9BXB1 | Class A Orphan/other | LGR like (hormone receptors) type 4 and 5 |
| OtR001 | Q9Z2H4 | Class A Orphan/other | LGR like (hormone receptors) type 4 and 5 |
| OtR002 | O88280 | Class A Orphan/other | LGR like (hormone receptors) type 4 and 5 |
| OtR002 | O75093 | Class A Orphan/other | LGR like (hormone receptors) type 4 and 5 |
| OtR002 | Q80TR4 | Class A Orphan/other | LGR like (hormone receptors) type 4 and 5 |
| OtR002 | Q9WVB4 | Class A Orphan/other | LGR like (hormone receptors) type 4 and 5 |
| OtR002 | Q9R1B9 | Class A Orphan/other | LGR like (hormone receptors) type 4 and 5 |
| OtR002 | O94813 | Class A Orphan/other | LGR like (hormone receptors) type 4 and 5 |
| OtR002 | O75094 | Class A Orphan/other | LGR like (hormone receptors) type 4 and 5 |
| OtR002 | O88279 | Class A Orphan/other | LGR like (hormone receptors) type 4 and 5 |
| OtR003 | Q5U9D9 | Class A Orphan/other | Mas proto-oncogene and Mas-related (MRGs) |
| OtR003 | Q4QXU4 | Class A Orphan/other | Mas proto-oncogene and Mas-related (MRGs) |
| OtR003 | Q4QXU2 | Class A Orphan/other | Mas proto-oncogene and Mas-related (MRGs) |
| OtR003 | Q96LB1 | Class A Orphan/other | Mas proto-oncogene and Mas-related (MRGs) |
| OtR003 | Q4QXU0 | Class A Orphan/other | Mas proto-oncogene and Mas-related (MRGs) |
| OtR003 | Q4QXU3 | Class A Orphan/other | Mas proto-oncogene and Mas-related (MRGs) |
| OtR003 | Q4QXU5 | Class A Orphan/other | Mas proto-oncogene and Mas-related (MRGs) |
| OtR003 | Q4QXX9 | Class A Orphan/other | Mas proto-oncogene and Mas-related (MRGs) |
| OtR004 | Q7TN49 | Class A Orphan/other | Mas proto-oncogene and Mas-related (MRGs) |
| OtR004 | Q91WW4 | Class A Orphan/other | Mas proto-oncogene and Mas-related (MRGs) |
| OtR004 | Q91ZC7 | Class A Orphan/other | Mas proto-oncogene and Mas-related (MRGs) |
| OtR004 | Q91ZC6 | Class A Orphan/other | Mas proto-oncogene and Mas-related (MRGs) |
| OtR004 | Q91WW5 | Class A Orphan/other | Mas proto-oncogene and Mas-related (MRGs) |
| OtR005 | Q8K1Z6 | Class A Orphan/other | GPR18 |
| OtR005 | Q3T0E9 | Class A Orphan/other | GPR18 |
| OtR005 | Q4R613 | Class A Orphan/other | GPR18 |
| OtR005 | A1A5S3 | Class A Orphan/other | GPR18 |
| OtR005 | Q14330 | Class A Orphan/other | GPR18 |
| OtR006 | Q9I919 | Class A Orphan/other | SREB |
| OtR006 | P60894 | Class A Orphan/other | SREB |
| OtR006 | P60893 | Class A Orphan/other | SREB |
| OtR006 | P60895 | Class A Orphan/other | SREB |
| OtR006 | Q5RBG7 | Class A Orphan/other | SREB |
| OtR007 | P60019 | Class A Orphan/other | GPR34 |
| OtR007 | Q9R1K6 | Class A Orphan/other | GPR34 |
| OtR007 | Q3SAG9 | Class A Orphan/other | GPR34 |
| OtR007 | Q9UPC5 | Class A Orphan/other | GPR34 |
| OtR007 | Q6XCF2 | Class A Orphan/other | GPR34 |
| OtR008 | Q9I918 | Class A Orphan/other | SREB |
| OtR008 | Q9JJH2 | Class A Orphan/other | SREB |
| OtR008 | Q9NS66 | Class A Orphan/other | SREB |
| OtR008 | Q6PI62 | Class A Orphan/other | SREB |
| OtR008 | Q5E9H8 | Class A Orphan/other | SREB |
| OtR009 | Q91ZC5 | Class A Orphan/other | Mas proto-oncogene and Mas-related (MRGs) |
| OtR009 | Q91WW2 | Class A Orphan/other | Mas proto-oncogene and Mas-related (MRGs) |
| OtR009 | Q91WW3 | Class A Orphan/other | Mas proto-oncogene and Mas-related (MRGs) |
| OtR009 | Q91ZC4 | Class A Orphan/other | Mas proto-oncogene and Mas-related (MRGs) |
| OtR010 | Q3UG61 | Class A Orphan/other | Mas proto-oncogene and Mas-related (MRGs) |
| OtR010 | Q3UG50 | Class A Orphan/other | Mas proto-oncogene and Mas-related (MRGs) |
| OtR010 | Q91ZC1 | Class A Orphan/other | Mas proto-oncogene and Mas-related (MRGs) |
| OtR010 | Q3KNA1 | Class A Orphan/other | Mas proto-oncogene and Mas-related (MRGs) |
| OtR011 | Q9Y271 | Class A Orphan/other | Cysteinyl leukotriene type 1 |
| OtR011 | Q99JA4 | Class A Orphan/other | Cysteinyl leukotriene type 1 |
| OtR011 | Q2NNR5 | Class A Orphan/other | Cysteinyl leukotriene type 1 |
| OtR011 | Q924T8 | Class A Orphan/other | Cysteinyl leukotriene type 1 |
| OtR012 | Q3U6B2 | Class A Orphan/other | EBV-induced |
| OtR012 | Q1RMI1 | Class A Orphan/other | EBV-induced |
| OtR012 | D4A7K7 | Class A Orphan/other | EBV-induced |
| OtR012 | P32249 | Class A Orphan/other | EBV-induced |
| OtR013 | Q5NUL3 | Class A Orphan/other | Other 512 |
| OtR013 | C8YUV0 | Class A Orphan/other | Other 512 |
| OtR013 | Q7TMA4 | Class A Orphan/other | Other 512 |
| OtR013 | Q2AC31 | Class A Orphan/other | Other 512 |
| OtR014 | P11613 | Class A Orphan/other | RDC1 |
| OtR014 | P25106 | Class A Orphan/other | RDC1 |
| OtR014 | O89039 | Class A Orphan/other | RDC1 |
| OtR014 | P56485 | Class A Orphan/other | RDC1 |
| OtR015 | Q5U431 | Class A Orphan/other | Other 511 |
| OtR015 | B2ZHY2 | Class A Orphan/other | Other 511 |
| OtR015 | O43194 | Class A Orphan/other | Other 511 |
| OtR015 | B4XF06 | Class A Orphan/other | Other 511 |
| OtR016 | Q96LB0 | Class A Orphan/other | Mas proto-oncogene and Mas-related (MRGs) |
| OtR016 | Q96LB2 | Class A Orphan/other | Mas proto-oncogene and Mas-related (MRGs) |
| OtR016 | Q96LA9 | Class A Orphan/other | Mas proto-oncogene and Mas-related (MRGs) |
| OtR016 | Q2LL16 | Class A Orphan/other | Mas proto-oncogene and Mas-related (MRGs) |
| OtR017 | Q86SM5 | Class A Orphan/other | Mas proto-oncogene and Mas-related (MRGs) |
| OtR017 | Q7TN39 | Class A Orphan/other | Mas proto-oncogene and Mas-related (MRGs) |
| OtR017 | Q91ZB5 | Class A Orphan/other | Mas proto-oncogene and Mas-related (MRGs) |
| OtR018 | B5X337 | Class A Orphan/other | EBV-induced |
| OtR018 | B0UXR0 | Class A Orphan/other | EBV-induced |
| OtR018 | A5PLE7 | Class A Orphan/other | EBV-induced |
| OtR019 | Q80SS6 | Class A Orphan/other | G-protein coupled bile acid receptor |
| OtR019 | Q862A9 | Class A Orphan/other | G-protein coupled bile acid receptor |
| OtR019 | Q80T02 | Class A Orphan/other | G-protein coupled bile acid receptor |
| OtR020 | Q924T9 | Class A Orphan/other | GPR17 |
| OtR020 | Q9NS75 | Class A Orphan/other | GPR17 |
| OtR020 | Q920A1 | Class A Orphan/other | GPR17 |
| OtR021 | Q76JU8 | Class A Orphan/other | Free fatty acid receptor 3 |
| OtR021 | Q8K3T4 | Class A Orphan/other | Free fatty acid receptor 3 |
| OtR021 | Q76JU9 | Class A Orphan/other | Free fatty acid receptor 3 |
| OtR022 | Q8C010 | Class A Orphan/other | Other 519 |
| OtR022 | Q9BZJ8 | Class A Orphan/other | Other 519 |
| OtR022 | Q91178 | Class A Orphan/other | Other 519 |
| OtR023 | P0C0W8 | Class A Orphan/other | GPR139 |
| OtR023 | Q80UC8 | Class A Orphan/other | GPR139 |
| OtR023 | Q6DWJ6 | Class A Orphan/other | GPR139 |
| OtR024 | P30554 | Class A Orphan/other | Mas proto-oncogene and Mas-related (MRGs) |
| OtR024 | P12526 | Class A Orphan/other | Mas proto-oncogene and Mas-related (MRGs) |
| OtR024 | P04201 | Class A Orphan/other | Mas proto-oncogene and Mas-related (MRGs) |
| OtR025 | Q8VCK6 | Class A Orphan/other | Free fatty acid receptor 3 |
| OtR025 | Q76EI6 | Class A Orphan/other | Free fatty acid receptor 3 |
| OtR025 | O15552 | Class A Orphan/other | Free fatty acid receptor 3 |
| OtR026 | Q8TDV0 | Class A Orphan/other | GPR151 like |
| OtR026 | Q7TSN6 | Class A Orphan/other | GPR151 like |
| OtR026 | Q7TSN5 | Class A Orphan/other | GPR151 like |
| OtR027 | Q96AM1 | Class A Orphan/other | Mas proto-oncogene and Mas-related (MRGs) |
| OtR027 | P23749 | Class A Orphan/other | Mas proto-oncogene and Mas-related (MRGs) |
| OtR027 | Q8VCJ6 | Class A Orphan/other | Mas proto-oncogene and Mas-related (MRGs) |
| OtR028 | Q09QM4 | Class A Orphan/other | GPR17 |
| OtR028 | Q13304 | Class A Orphan/other | GPR17 |
| OtR028 | Q6NS65 | Class A Orphan/other | GPR17 |
| OtR029 | Q9EPB7 | Class A Orphan/other | GPR88 |
| OtR029 | Q9GZN0 | Class A Orphan/other | GPR88 |
| OtR029 | Q9ESP4 | Class A Orphan/other | GPR88 |
| OtR030 | Q9JJH3 | Class A Orphan/other | SREB |
| OtR030 | Q9NS67 | Class A Orphan/other | SREB |
| OtR030 | O54897 | Class A Orphan/other | SREB |
| OtR031 | Q5QD04 | Class A Orphan/other | GPR3, 6 and 12 |
| OtR031 | Q96RI9 | Class A Orphan/other | GPR3, 6 and 12 |
| OtR031 | Q923Y6 | Class A Orphan/other | GPR3, 6 and 12 |
| OtR032 | Q14439 | Class A Orphan/other | ORPH_MSC429 |
| OtR032 | Q80WT4 | Class A Orphan/other | ORPH_MSC429 |
| OtR032 | Q64017 | Class A Orphan/other | ORPH_MSC429 |
| OtR033 | Q7TN50 | Class A Orphan/other | Mas proto-oncogene and Mas-related (MRGs) |
| OtR033 | Q7TN51 | Class A Orphan/other | Mas proto-oncogene and Mas-related (MRGs) |
| OtR034 | F8VQN3 | Class A Orphan/other | GPR31 |
| OtR034 | O00270 | Class A Orphan/other | GPR31 |
| OtR035 | Q9ES90 | Class A Orphan/other | Other 519 |
| OtR035 | Q9HC97 | Class A Orphan/other | Other 519 |
| OtR036 | Q9BZJ7 | Class A Orphan/other | Other 519 |
| OtR036 | Q80UC6 | Class A Orphan/other | Other 519 |
| OtR037 | Q91ZC0 | Class A Orphan/other | Mas proto-oncogene and Mas-related (MRGs) |
| OtR037 | Q91ZB9 | Class A Orphan/other | Mas proto-oncogene and Mas-related (MRGs) |
| OtR038 | Q862A8 | Class A Orphan/other | G-protein coupled bile acid receptor |
| OtR038 | Q8TDU6 | Class A Orphan/other | G-protein coupled bile acid receptor |
| OtR039 | Q7TQN9 | Class A Orphan/other | GPR139 |
| OtR039 | Q7Z601 | Class A Orphan/other | GPR139 |
| OtR040 | Q7TN40 | Class A Orphan/other | Mas proto-oncogene and Mas-related (MRGs) |
| OtR040 | Q91ZB7 | Class A Orphan/other | Mas proto-oncogene and Mas-related (MRGs) |
| OtR041 | Q86SM8 | Class A Orphan/other | Mas proto-oncogene and Mas-related (MRGs) |
| OtR041 | Q5U9D7 | Class A Orphan/other | Mas proto-oncogene and Mas-related (MRGs) |
| OtR042 | Q76JV1 | Class A Orphan/other | Free fatty acid receptor 3 |
| OtR042 | O14842 | Class A Orphan/other | Free fatty acid receptor 3 |
| OtR043 | Q7TN44 | Class A Orphan/other | Mas proto-oncogene and Mas-related (MRGs) |
| OtR043 | Q7TN45 | Class A Orphan/other | Mas proto-oncogene and Mas-related (MRGs) |
| OtR044 | Q8CIP3 | Class A Orphan/other | Mas proto-oncogene and Mas-related (MRGs) |
| OtR044 | Q8R4G1 | Class A Orphan/other | Mas proto-oncogene and Mas-related (MRGs) |
| OtR045 | Q7TN41 | Class A Orphan/other | Mas proto-oncogene and Mas-related (MRGs) |
| OtR045 | Q91ZB8 | Class A Orphan/other | Mas proto-oncogene and Mas-related (MRGs) |
| OtR046 | Q8TDS7 | Class A Orphan/other | Mas proto-oncogene and Mas-related (MRGs) |
| OtR046 | Q6L786 | Class A Orphan/other | Mas proto-oncogene and Mas-related (MRGs) |
| OtR047 | O14843 | Class A Orphan/other | Free fatty acid receptor 3 |
| OtR047 | O15529 | Class A Orphan/other | Free fatty acid receptor 3 |
| OtR048 | Q3UFD7 | Class A Orphan/other | Free fatty acid receptor 3 |
| OtR048 | B2GV46 | Class A Orphan/other | Free fatty acid receptor 3 |
| OtR049 | Q99MT8 | Class A Orphan/other | Mas proto-oncogene and Mas-related (MRGs) |
| OtR049 | Q7TN38 | Class A Orphan/other | Mas proto-oncogene and Mas-related (MRGs) |
| OtR050 | Q9BXC1 | Class A Orphan/other | Other 519 |
| OtR050 | Q3U507 | Class A Orphan/other | Other 519 |
| Pa001 | P41593 | Parathyroid hormone receptor | Parathyroid hormone |
| Pa001 | Q03431 | Parathyroid hormone receptor | Parathyroid hormone |
| Pa001 | Q9TU31 | Parathyroid hormone receptor | Parathyroid hormone |
| Pa001 | P25107 | Parathyroid hormone receptor | Parathyroid hormone |
| Pa001 | P50133 | Parathyroid hormone receptor | Parathyroid hormone |
| Pa001 | P25961 | Parathyroid hormone receptor | Parathyroid hormone |
| Pa001 | Q5RAQ1 | Parathyroid hormone receptor | Parathyroid hormone |
| Pa001 | Q1LZF7 | Parathyroid hormone receptor | Parathyroid hormone |
| Pa002 | P49190 | Parathyroid hormone receptor | Parathyroid hormone |
| Pa002 | Q91V95 | Parathyroid hormone receptor | Parathyroid hormone |
| Pe001 | Q95NC7 | Peptide receptor | C-C Chemokine type 5 |
| Pe001 | O97879 | Peptide receptor | C-C Chemokine type 5 |
| Pe001 | P56493 | Peptide receptor | C-C Chemokine type 5 |
| Pe001 | Q95NC4 | Peptide receptor | C-C Chemokine type 5 |
| Pe001 | Q95NC1 | Peptide receptor | C-C Chemokine type 5 |
| Pe001 | O08556 | Peptide receptor | C-C Chemokine type 5 |
| Pe001 | Q2HJ17 | Peptide receptor | C-C Chemokine type 5 |
| Pe001 | O62743 | Peptide receptor | C-C Chemokine type 5 |
| Pe001 | Q9XT76 | Peptide receptor | C-C Chemokine type 5 |
| Pe001 | Q95NC9 | Peptide receptor | C-C Chemokine type 5 |
| Pe001 | Q5ECR9 | Peptide receptor | C-C Chemokine type 5 |
| Pe001 | P68269 | Peptide receptor | C-C Chemokine type 5 |
| Pe001 | P61815 | Peptide receptor | C-C Chemokine type 5 |
| Pe001 | P51681 | Peptide receptor | C-C Chemokine type 5 |
| Pe001 | Q9TV49 | Peptide receptor | C-C Chemokine type 5 |
| Pe001 | P56440 | Peptide receptor | C-C Chemokine type 5 |
| Pe001 | O97883 | Peptide receptor | C-C Chemokine type 5 |
| Pe001 | Q95NC3 | Peptide receptor | C-C Chemokine type 5 |
| Pe001 | P61814 | Peptide receptor | C-C Chemokine type 5 |
| Pe001 | Q95NC8 | Peptide receptor | C-C Chemokine type 5 |
| Pe001 | Q95ND2 | Peptide receptor | C-C Chemokine type 5 |
| Pe001 | Q9TV45 | Peptide receptor | C-C Chemokine type 5 |
| Pe001 | O97878 | Peptide receptor | C-C Chemokine type 5 |
| Pe001 | P68270 | Peptide receptor | C-C Chemokine type 5 |
| Pe001 | P61756 | Peptide receptor | C-C Chemokine type 5 |
| Pe001 | O97882 | Peptide receptor | C-C Chemokine type 5 |
| Pe001 | Q9TV48 | Peptide receptor | C-C Chemokine type 5 |
| Pe001 | Q95NC6 | Peptide receptor | C-C Chemokine type 5 |
| Pe001 | Q1ZY22 | Peptide receptor | C-C Chemokine type 5 |
| Pe001 | P61757 | Peptide receptor | C-C Chemokine type 5 |
| Pe001 | Q95ND1 | Peptide receptor | C-C Chemokine type 5 |
| Pe001 | Q9TV43 | Peptide receptor | C-C Chemokine type 5 |
| Pe001 | Q9BGN6 | Peptide receptor | C-C Chemokine type 5 |
| Pe001 | O97880 | Peptide receptor | C-C Chemokine type 5 |
| Pe001 | O97881 | Peptide receptor | C-C Chemokine type 5 |
| Pe001 | Q8HZT9 | Peptide receptor | C-C Chemokine type 5 |
| Pe001 | P51682 | Peptide receptor | C-C Chemokine type 5 |
| Pe001 | Q9TV42 | Peptide receptor | C-C Chemokine type 5 |
| Pe001 | O97962 | Peptide receptor | C-C Chemokine type 5 |
| Pe001 | Q95NC0 | Peptide receptor | C-C Chemokine type 5 |
| Pe001 | P61755 | Peptide receptor | C-C Chemokine type 5 |
| Pe001 | P60574 | Peptide receptor | C-C Chemokine type 5 |
| Pe001 | O97975 | Peptide receptor | C-C Chemokine type 5 |
| Pe001 | Q95NE8 | Peptide receptor | C-C Chemokine type 5 |
| Pe001 | P61813 | Peptide receptor | C-C Chemokine type 5 |
| Pe001 | P56439 | Peptide receptor | C-C Chemokine type 5 |
| Pe001 | Q9TV47 | Peptide receptor | C-C Chemokine type 5 |
| Pe001 | Q95NC2 | Peptide receptor | C-C Chemokine type 5 |
| Pe001 | Q95ND0 | Peptide receptor | C-C Chemokine type 5 |
| Pe001 | Q6WN98 | Peptide receptor | C-C Chemokine type 5 |
| Pe001 | Q95NC5 | Peptide receptor | C-C Chemokine type 5 |
| Pe002 | Q864J4 | Peptide receptor | Melanocortin type 1 |
| Pe002 | Q864J2 | Peptide receptor | Melanocortin type 1 |
| Pe002 | Q01726 | Peptide receptor | Melanocortin type 1 |
| Pe002 | Q864L0 | Peptide receptor | Melanocortin type 1 |
| Pe002 | Q864J8 | Peptide receptor | Melanocortin type 1 |
| Pe002 | Q9TUK4 | Peptide receptor | Melanocortin type 1 |
| Pe002 | Q864G6 | Peptide receptor | Melanocortin type 1 |
| Pe002 | Q864G3 | Peptide receptor | Melanocortin type 1 |
| Pe002 | Q864J1 | Peptide receptor | Melanocortin type 1 |
| Pe002 | Q864I3 | Peptide receptor | Melanocortin type 1 |
| Pe002 | Q864I2 | Peptide receptor | Melanocortin type 1 |
| Pe002 | Q864H7 | Peptide receptor | Melanocortin type 1 |
| Pe002 | Q864G1 | Peptide receptor | Melanocortin type 1 |
| Pe002 | Q864K9 | Peptide receptor | Melanocortin type 1 |
| Pe002 | Q864K1 | Peptide receptor | Melanocortin type 1 |
| Pe002 | Q864I7 | Peptide receptor | Melanocortin type 1 |
| Pe002 | Q864H5 | Peptide receptor | Melanocortin type 1 |
| Pe002 | Q864H9 | Peptide receptor | Melanocortin type 1 |
| Pe002 | Q864K8 | Peptide receptor | Melanocortin type 1 |
| Pe002 | Q864J5 | Peptide receptor | Melanocortin type 1 |
| Pe002 | Q864I9 | Peptide receptor | Melanocortin type 1 |
| Pe002 | Q864J6 | Peptide receptor | Melanocortin type 1 |
| Pe002 | Q864I8 | Peptide receptor | Melanocortin type 1 |
| Pe002 | Q864G9 | Peptide receptor | Melanocortin type 1 |
| Pe002 | Q864K4 | Peptide receptor | Melanocortin type 1 |
| Pe002 | Q864I4 | Peptide receptor | Melanocortin type 1 |
| Pe002 | Q864I0 | Peptide receptor | Melanocortin type 1 |
| Pe002 | Q864K2 | Peptide receptor | Melanocortin type 1 |
| Pe002 | Q0Q460 | Peptide receptor | Melanocortin type 1 |
| Pe002 | Q864K6 | Peptide receptor | Melanocortin type 1 |
| Pe002 | Q864G4 | Peptide receptor | Melanocortin type 1 |
| Pe002 | Q864K5 | Peptide receptor | Melanocortin type 1 |
| Pe002 | Q864I6 | Peptide receptor | Melanocortin type 1 |
| Pe002 | Q864H2 | Peptide receptor | Melanocortin type 1 |
| Pe002 | Q864I5 | Peptide receptor | Melanocortin type 1 |
| Pe002 | Q864H3 | Peptide receptor | Melanocortin type 1 |
| Pe002 | Q864J3 | Peptide receptor | Melanocortin type 1 |
| Pe002 | Q864I1 | Peptide receptor | Melanocortin type 1 |
| Pe002 | Q864H4 | Peptide receptor | Melanocortin type 1 |
| Pe002 | Q864K0 | Peptide receptor | Melanocortin type 1 |
| Pe002 | Q864J7 | Peptide receptor | Melanocortin type 1 |
| Pe002 | Q864G2 | Peptide receptor | Melanocortin type 1 |
| Pe002 | Q864J9 | Peptide receptor | Melanocortin type 1 |
| Pe002 | Q864H0 | Peptide receptor | Melanocortin type 1 |
| Pe002 | Q864J0 | Peptide receptor | Melanocortin type 1 |
| Pe002 | Q864K3 | Peptide receptor | Melanocortin type 1 |
| Pe002 | Q864H6 | Peptide receptor | Melanocortin type 1 |
| Pe003 | P61072 | Peptide receptor | C-X-C Chemokine type 4 |
| Pe003 | Q8HZU0 | Peptide receptor | C-X-C Chemokine type 4 |
| Pe003 | P25930 | Peptide receptor | C-X-C Chemokine type 4 |
| Pe003 | P70658 | Peptide receptor | C-X-C Chemokine type 4 |
| Pe003 | P56491 | Peptide receptor | C-X-C Chemokine type 4 |
| Pe003 | Q764M9 | Peptide receptor | C-X-C Chemokine type 4 |
| Pe003 | O08565 | Peptide receptor | C-X-C Chemokine type 4 |
| Pe003 | P79394 | Peptide receptor | C-X-C Chemokine type 4 |
| Pe003 | Q9TSQ8 | Peptide receptor | C-X-C Chemokine type 4 |
| Pe003 | Q28474 | Peptide receptor | C-X-C Chemokine type 4 |
| Pe003 | Q8HZU1 | Peptide receptor | C-X-C Chemokine type 4 |
| Pe003 | Q3LSL6 | Peptide receptor | C-X-C Chemokine type 4 |
| Pe003 | P56498 | Peptide receptor | C-X-C Chemokine type 4 |
| Pe003 | P61073 | Peptide receptor | C-X-C Chemokine type 4 |
| Pe003 | Q7YS92 | Peptide receptor | C-X-C Chemokine type 4 |
| Pe003 | O62747 | Peptide receptor | C-X-C Chemokine type 4 |
| Pe004 | Q9WV26 | Peptide receptor | Angiotensin type 1 |
| Pe004 | P29754 | Peptide receptor | Angiotensin type 1 |
| Pe004 | P43240 | Peptide receptor | Angiotensin type 1 |
| Pe004 | P25095 | Peptide receptor | Angiotensin type 1 |
| Pe004 | P30555 | Peptide receptor | Angiotensin type 1 |
| Pe004 | Q9GLN9 | Peptide receptor | Angiotensin type 1 |
| Pe004 | P34976 | Peptide receptor | Angiotensin type 1 |
| Pe004 | P29089 | Peptide receptor | Angiotensin type 1 |
| Pe004 | O77590 | Peptide receptor | Angiotensin type 1 |
| Pe004 | P29755 | Peptide receptor | Angiotensin type 1 |
| Pe004 | P33396 | Peptide receptor | Angiotensin type 1 |
| Pe004 | P25104 | Peptide receptor | Angiotensin type 1 |
| Pe004 | O35210 | Peptide receptor | Angiotensin type 1 |
| Pe004 | P30556 | Peptide receptor | Angiotensin type 1 |
| Pe004 | P79785 | Peptide receptor | Angiotensin type 1 |
| Pe005 | O19037 | Peptide receptor | Melanocortin type 1 |
| Pe005 | P55167 | Peptide receptor | Melanocortin type 1 |
| Pe005 | P56447 | Peptide receptor | Melanocortin type 1 |
| Pe005 | P56446 | Peptide receptor | Melanocortin type 1 |
| Pe005 | P56445 | Peptide receptor | Melanocortin type 1 |
| Pe005 | P56442 | Peptide receptor | Melanocortin type 1 |
| Pe005 | P56448 | Peptide receptor | Melanocortin type 1 |
| Pe005 | P47798 | Peptide receptor | Melanocortin type 1 |
| Pe005 | P56444 | Peptide receptor | Melanocortin type 1 |
| Pe005 | P56443 | Peptide receptor | Melanocortin type 1 |
| Pe006 | Q9N0W7 | Peptide receptor | Endothelin type B |
| Pe006 | P21451 | Peptide receptor | Endothelin type B |
| Pe006 | P35463 | Peptide receptor | Endothelin type B |
| Pe006 | Q90328 | Peptide receptor | Endothelin type B |
| Pe006 | P32940 | Peptide receptor | Endothelin type B |
| Pe006 | O62709 | Peptide receptor | Endothelin type B |
| Pe006 | P48302 | Peptide receptor | Endothelin type B |
| Pe006 | P28088 | Peptide receptor | Endothelin type B |
| Pe006 | P56497 | Peptide receptor | Endothelin type B |
| Pe006 | P24530 | Peptide receptor | Endothelin type B |
| Pe007 | Q95LF7 | Peptide receptor | Duffy antigen |
| Pe007 | Q95LG5 | Peptide receptor | Duffy antigen |
| Pe007 | Q9QUI6 | Peptide receptor | Duffy antigen |
| Pe007 | Q95LF5 | Peptide receptor | Duffy antigen |
| Pe007 | Q95LF4 | Peptide receptor | Duffy antigen |
| Pe007 | Q16570 | Peptide receptor | Duffy antigen |
| Pe007 | Q95LF2 | Peptide receptor | Duffy antigen |
| Pe007 | Q95LF9 | Peptide receptor | Duffy antigen |
| Pe007 | Q95LF3 | Peptide receptor | Duffy antigen |
| Pe008 | Q90334 | Peptide receptor | Isotocin 8 |
| Pe008 | P32306 | Peptide receptor | Mesotocin |
| Pe008 | Q90252 | Peptide receptor | Mesotocin |
| Pe008 | P97926 | Peptide receptor | Mesotocin |
| Pe008 | P56449 | Peptide receptor | Mesotocin |
| Pe008 | P56494 | Peptide receptor | Mesotocin |
| Pe008 | Q28756 | Peptide receptor | Mesotocin |
| Pe008 | P30559 | Peptide receptor | Mesotocin |
| Pe008 | P70536 | Peptide receptor | Mesotocin |
| Pe009 | Q95247 | Peptide receptor | Opioid type M |
| Pe009 | P79350 | Peptide receptor | Opioid type M |
| Pe009 | Q9MYW9 | Peptide receptor | Opioid type M |
| Pe009 | P33535 | Peptide receptor | Opioid type M |
| Pe009 | Q5IS84 | Peptide receptor | Opioid type M |
| Pe009 | Q95M54 | Peptide receptor | Opioid type M |
| Pe009 | Q5IS39 | Peptide receptor | Opioid type M |
| Pe009 | P35372 | Peptide receptor | Opioid type M |
| Pe009 | P42866 | Peptide receptor | Opioid type M |
| Pe010 | P46663 | Peptide receptor | Bradykinin type B1 |
| Pe010 | Q95L01 | Peptide receptor | Bradykinin type B1 |
| Pe010 | Q8HZP2 | Peptide receptor | Bradykinin type B1 |
| Pe010 | Q8HZN9 | Peptide receptor | Bradykinin type B1 |
| Pe010 | Q3BCU0 | Peptide receptor | Bradykinin type B1 |
| Pe010 | P48748 | Peptide receptor | Bradykinin type B1 |
| Pe010 | Q9BDQ5 | Peptide receptor | Bradykinin type B1 |
| Pe010 | Q8HZP1 | Peptide receptor | Bradykinin type B1 |
| Pe010 | Q8HZP3 | Peptide receptor | Bradykinin type B1 |
| Pe011 | Q5IS62 | Peptide receptor | Neuropeptide Y type 2 |
| Pe011 | P97295 | Peptide receptor | Neuropeptide Y type 2 |
| Pe011 | Q9DDN6 | Peptide receptor | Neuropeptide Y type 2 |
| Pe011 | P49146 | Peptide receptor | Neuropeptide Y type 2 |
| Pe011 | Q9Z2D5 | Peptide receptor | Neuropeptide Y type 2 |
| Pe011 | O02836 | Peptide receptor | Neuropeptide Y type 2 |
| Pe011 | Q9GK74 | Peptide receptor | Neuropeptide Y type 2 |
| Pe011 | P79113 | Peptide receptor | Neuropeptide Y type 2 |
| Pe012 | Q8K458 | Peptide receptor | Prokineticin receptors |
| Pe012 | Q8R416 | Peptide receptor | Prokineticin receptors |
| Pe012 | Q8TCW9 | Peptide receptor | Prokineticin receptors |
| Pe012 | Q8SPN1 | Peptide receptor | Prokineticin receptors |
| Pe012 | Q8R415 | Peptide receptor | Prokineticin receptors |
| Pe012 | Q9JKL1 | Peptide receptor | Prokineticin receptors |
| Pe012 | Q8SPN2 | Peptide receptor | Prokineticin receptors |
| Pe012 | Q8NFJ6 | Peptide receptor | Prokineticin receptors |
| Pe013 | P16610 | Peptide receptor | Substance K (NK2) |
| Pe013 | P05363 | Peptide receptor | Substance K (NK2) |
| Pe013 | Q64077 | Peptide receptor | Substance K (NK2) |
| Pe013 | Q5DUB2 | Peptide receptor | Substance K (NK2) |
| Pe013 | P21452 | Peptide receptor | Substance K (NK2) |
| Pe013 | P51144 | Peptide receptor | Substance K (NK2) |
| Pe013 | P30549 | Peptide receptor | Substance K (NK2) |
| Pe013 | P79218 | Peptide receptor | Substance K (NK2) |
| Pe014 | Q2YEG0 | Peptide receptor | Interleukin-8 type A |
| Pe014 | P55919 | Peptide receptor | Interleukin-8 type A |
| Pe014 | Q28003 | Peptide receptor | Interleukin-8 type B |
| Pe014 | Q2YEG2 | Peptide receptor | Interleukin-8 type A |
| Pe014 | P55920 | Peptide receptor | Interleukin-8 type A |
| Pe014 | P21109 | Peptide receptor | Interleukin-8 type A |
| Pe014 | P25024 | Peptide receptor | Interleukin-8 type A |
| Pe014 | Q2YEF9 | Peptide receptor | Interleukin-8 type A |
| Pe015 | O43614 | Peptide receptor | Orexin type 2 |
| Pe015 | Q0GBZ5 | Peptide receptor | Orexin type 1 |
| Pe015 | P58307 | Peptide receptor | Orexin type 1 |
| Pe015 | P58308 | Peptide receptor | Orexin type 2 |
| Pe015 | P56719 | Peptide receptor | Orexin type 2 |
| Pe015 | O43613 | Peptide receptor | Orexin type 1 |
| Pe015 | P56718 | Peptide receptor | Orexin type 1 |
| Pe015 | Q9TUP7 | Peptide receptor | Orexin type 2 |
| Pe016 | Q1RMU8 | Peptide receptor | Neuropeptide Y type 1 |
| Pe016 | P25929 | Peptide receptor | Neuropeptide Y type 1 |
| Pe016 | Q04573 | Peptide receptor | Neuropeptide Y type 1 |
| Pe016 | P34992 | Peptide receptor | Neuropeptide Y type 1 |
| Pe016 | O02835 | Peptide receptor | Neuropeptide Y type 1 |
| Pe016 | Q9WVD0 | Peptide receptor | Neuropeptide Y type 1 |
| Pe016 | O02813 | Peptide receptor | Neuropeptide Y type 1 |
| Pe016 | P21555 | Peptide receptor | Neuropeptide Y type 1 |
| Pe017 | P56481 | Peptide receptor | CCK 2 |
| Pe017 | P30552 | Peptide receptor | CCK 2 |
| Pe017 | P46627 | Peptide receptor | CCK 2 |
| Pe017 | P30796 | Peptide receptor | CCK 2 |
| Pe017 | P70031 | Peptide receptor | CCK 3 |
| Pe017 | P32239 | Peptide receptor | CCK 2 |
| Pe017 | P30553 | Peptide receptor | CCK 2 |
| Pe017 | P79266 | Peptide receptor | CCK 2 |
| Pe018 | P79234 | Peptide receptor | C5a anaphylatoxin |
| Pe018 | P30992 | Peptide receptor | C5a anaphylatoxin |
| Pe018 | O70129 | Peptide receptor | C5a anaphylatoxin |
| Pe018 | P79175 | Peptide receptor | C5a anaphylatoxin |
| Pe018 | P79240 | Peptide receptor | C5a anaphylatoxin |
| Pe018 | P21730 | Peptide receptor | C5a anaphylatoxin |
| Pe018 | Q9TUE1 | Peptide receptor | C5a anaphylatoxin |
| Pe018 | P79188 | Peptide receptor | C5a anaphylatoxin |
| Pe019 | Q8HXX3 | Peptide receptor | Melanocortin type 4 |
| Pe019 | P56450 | Peptide receptor | Melanocortin type 4 |
| Pe019 | Q9GLJ8 | Peptide receptor | Melanocortin type 4 |
| Pe019 | P70596 | Peptide receptor | Melanocortin type 4 |
| Pe019 | P32245 | Peptide receptor | Melanocortin type 4 |
| Pe019 | O97504 | Peptide receptor | Melanocortin type 4 |
| Pe019 | Q0Z8I9 | Peptide receptor | Melanocortin type 4 |
| Pe019 | B0V1P1 | Peptide receptor | Melanocortin type 4 |
| Pe020 | Q9TV16 | Peptide receptor | C-X-C Chemokine type 6 (Bonzo) |
| Pe020 | Q9EQ16 | Peptide receptor | C-X-C Chemokine type 6 (Bonzo) |
| Pe020 | O19024 | Peptide receptor | C-X-C Chemokine type 6 (Bonzo) |
| Pe020 | Q9XT45 | Peptide receptor | C-X-C Chemokine type 6 (Bonzo) |
| Pe020 | Q9N0Z0 | Peptide receptor | C-X-C Chemokine type 6 (Bonzo) |
| Pe020 | O00574 | Peptide receptor | C-X-C Chemokine type 6 (Bonzo) |
| Pe020 | O18983 | Peptide receptor | C-X-C Chemokine type 6 (Bonzo) |
| Pe020 | Q9BDS6 | Peptide receptor | C-X-C Chemokine type 6 (Bonzo) |
| Pe021 | Q5DUB3 | Peptide receptor | Substance P (NK1) |
| Pe021 | Q98982 | Peptide receptor | Substance P (NK1) |
| Pe021 | P25103 | Peptide receptor | Substance P (NK1) |
| Pe021 | P14600 | Peptide receptor | Substance P (NK1) |
| Pe021 | Q5DUB1 | Peptide receptor | Substance P (NK1) |
| Pe021 | P30548 | Peptide receptor | Substance P (NK1) |
| Pe021 | P30547 | Peptide receptor | Substance P (NK1) |
| Pe022 | Q61614 | Peptide receptor | Endothelin type A |
| Pe022 | P26684 | Peptide receptor | Endothelin type A |
| Pe022 | Q95L55 | Peptide receptor | Endothelin type A |
| Pe022 | Q5KSU9 | Peptide receptor | Endothelin type A |
| Pe022 | P21450 | Peptide receptor | Endothelin type A |
| Pe022 | Q29010 | Peptide receptor | Endothelin type A |
| Pe022 | P25101 | Peptide receptor | Endothelin type A |
| Pe023 | P21462 | Peptide receptor | Fmet-leu-phe |
| Pe023 | Q05394 | Peptide receptor | Fmet-leu-phe |
| Pe023 | P79235 | Peptide receptor | Fmet-leu-phe |
| Pe023 | P79176 | Peptide receptor | Fmet-leu-phe |
| Pe023 | P79189 | Peptide receptor | Fmet-leu-phe |
| Pe023 | P33766 | Peptide receptor | Fmet-leu-phe |
| Pe023 | P79241 | Peptide receptor | Fmet-leu-phe |
| Pe024 | Q9BG77 | Peptide receptor | GPR15 |
| Pe024 | Q9BDS7 | Peptide receptor | GPR15 |
| Pe024 | P56412 | Peptide receptor | GPR15 |
| Pe024 | P49685 | Peptide receptor | GPR15 |
| Pe024 | O18982 | Peptide receptor | GPR15 |
| Pe024 | Q0VDU3 | Peptide receptor | APJ like Non Vertebrate type 1 |
| Pe024 | O97663 | Peptide receptor | GPR15 |
| Pe025 | P30558 | Peptide receptor | Proteinase-activated type 1 |
| Pe025 | P25116 | Peptide receptor | Proteinase-activated type 1 |
| Pe025 | P56488 | Peptide receptor | Proteinase-activated type 1 |
| Pe025 | P47749 | Peptide receptor | Proteinase-activated type 1 |
| Pe025 | P26824 | Peptide receptor | Proteinase-activated type 1 |
| Pe025 | A7YY44 | Peptide receptor | Proteinase-activated type 1 |
| Pe025 | Q00991 | Peptide receptor | Proteinase-activated type 1 |
| Pe026 | P30975 | Peptide receptor | Tachykinin like 2 |
| Pe026 | P16177 | Peptide receptor | Neuromedin K (NK3) |
| Pe026 | O97512 | Peptide receptor | Neuromedin K (NK3) |
| Pe026 | P30098 | Peptide receptor | Neuromedin K (NK3) |
| Pe026 | P47937 | Peptide receptor | Neuromedin K (NK3) |
| Pe026 | P30974 | Peptide receptor | Tachykinin like 1 |
| Pe026 | P29371 | Peptide receptor | Neuromedin K (NK3) |
| Pe027 | Q6H2Y3 | Peptide receptor | Bombesin type 3 |
| Pe027 | O97967 | Peptide receptor | Bombesin type 3 |
| Pe027 | P35371 | Peptide receptor | Bombesin type 3 |
| Pe027 | O54798 | Peptide receptor | Bombesin type 3 |
| Pe027 | Q8K418 | Peptide receptor | Bombesin type 3 |
| Pe027 | P47751 | Peptide receptor | Bombesin type 4 |
| Pe027 | P32247 | Peptide receptor | Bombesin type 3 |
| Pe028 | Q9WTV8 | Peptide receptor | Vasopressin type 1a |
| Pe028 | Q90352 | Peptide receptor | Vasotocin |
| Pe028 | P48043 | Peptide receptor | Vasopressin type 1a |
| Pe028 | Q62463 | Peptide receptor | Vasopressin type 1a |
| Pe028 | P30560 | Peptide receptor | Vasopressin type 1a |
| Pe028 | Q9WTV9 | Peptide receptor | Vasopressin type 1a |
| Pe028 | P37288 | Peptide receptor | Vasopressin type 1a |
| Pe029 | P30874 | Peptide receptor | Somatostatin type 2 |
| Pe029 | P34993 | Peptide receptor | Somatostatin type 2 |
| Pe029 | P30875 | Peptide receptor | Somatostatin type 2 |
| Pe029 | P34994 | Peptide receptor | Somatostatin type 2 |
| Pe029 | P30680 | Peptide receptor | Somatostatin type 2 |
| Pe029 | Q49LX6 | Peptide receptor | Somatostatin type 2 |
| Pe030 | O08786 | Peptide receptor | CCK 1 |
| Pe030 | Q63931 | Peptide receptor | CCK 1 |
| Pe030 | P30551 | Peptide receptor | CCK 1 |
| Pe030 | O97772 | Peptide receptor | CCK 1 |
| Pe030 | P32238 | Peptide receptor | CCK 1 |
| Pe030 | Q5D0K2 | Peptide receptor | CCK 1 |
| Pe031 | Q28642 | Peptide receptor | Bradykinin type B2 |
| Pe031 | O70526 | Peptide receptor | Bradykinin type B2 |
| Pe031 | P25023 | Peptide receptor | Bradykinin type B2 |
| Pe031 | Q9GLX8 | Peptide receptor | Bradykinin type B2 |
| Pe031 | P32299 | Peptide receptor | Bradykinin type B2 |
| Pe031 | P30411 | Peptide receptor | Bradykinin type B2 |
| Pe032 | Q00788 | Peptide receptor | Vasopressin type 2 |
| Pe032 | O77808 | Peptide receptor | Vasopressin type 2 |
| Pe032 | P48044 | Peptide receptor | Vasopressin type 2 |
| Pe032 | P32307 | Peptide receptor | Vasopressin type 2 |
| Pe032 | P30518 | Peptide receptor | Vasopressin type 2 |
| Pe032 | O88721 | Peptide receptor | Vasopressin type 2 |
| Pe033 | O88410 | Peptide receptor | C-X-C Chemokine type 3 |
| Pe033 | Q867B2 | Peptide receptor | C-X-C Chemokine type 3 |
| Pe033 | P49682 | Peptide receptor | C-X-C Chemokine type 3 |
| Pe033 | Q9JII9 | Peptide receptor | C-X-C Chemokine type 3 |
| Pe033 | Q5MD61 | Peptide receptor | C-X-C Chemokine type 3 |
| Pe033 | Q5KSK8 | Peptide receptor | C-X-C Chemokine type 3 |
| Pe034 | O88680 | Peptide receptor | C3a anaphylatoxin |
| Pe034 | Q16581 | Peptide receptor | C3a anaphylatoxin |
| Pe034 | O55197 | Peptide receptor | C3a anaphylatoxin |
| Pe034 | Q6TAC8 | Peptide receptor | C3a anaphylatoxin |
| Pe034 | Q5REI5 | Peptide receptor | C3a anaphylatoxin |
| Pe034 | O09047 | Peptide receptor | C3a anaphylatoxin |
| Pe035 | P25025 | Peptide receptor | Interleukin-8 type B |
| Pe035 | P35344 | Peptide receptor | Interleukin-8 type B |
| Pe035 | Q28807 | Peptide receptor | Interleukin-8 type B |
| Pe035 | O97571 | Peptide receptor | Interleukin-8 type B |
| Pe035 | Q28519 | Peptide receptor | Interleukin-8 type B |
| Pe035 | Q28422 | Peptide receptor | Interleukin-8 type B |
| Pe036 | P25089 | Peptide receptor | Fmet-leu-phe |
| Pe036 | P79191 | Peptide receptor | Fmet-leu-phe |
| Pe036 | P79243 | Peptide receptor | Fmet-leu-phe |
| Pe036 | P79178 | Peptide receptor | Fmet-leu-phe |
| Pe036 | P79237 | Peptide receptor | Fmet-leu-phe |
| Pe037 | P51678 | Peptide receptor | C-C Chemokine type 3 |
| Pe037 | O54814 | Peptide receptor | C-C Chemokine type 3 |
| Pe037 | Q64H34 | Peptide receptor | C-C Chemokine type 3 |
| Pe037 | Q9Z2I3 | Peptide receptor | C-C Chemokine type 3 |
| Pe037 | Q89609 | Peptide receptor | C-C Chemokine type 3 |
| Pe038 | Q8MJ89 | Peptide receptor | Melanin-concentrating hormone receptors |
| Pe038 | P97639 | Peptide receptor | Melanin-concentrating hormone receptors |
| Pe038 | Q8JZL2 | Peptide receptor | Melanin-concentrating hormone receptors |
| Pe038 | Q5IJ49 | Peptide receptor | Melanin-concentrating hormone receptors |
| Pe038 | Q99705 | Peptide receptor | Melanin-concentrating hormone receptors |
| Pe039 | P46090 | Peptide receptor | Fmet-leu-phe |
| Pe039 | O97664 | Peptide receptor | Fmet-leu-phe |
| Pe039 | P46091 | Peptide receptor | Fmet-leu-phe |
| Pe039 | Q8K087 | Peptide receptor | Fmet-leu-phe |
| Pe039 | Q95LH1 | Peptide receptor | Fmet-leu-phe |
| Pe040 | Q865E5 | Peptide receptor | Melanocortin type 1 |
| Pe040 | Q9TU05 | Peptide receptor | Melanocortin type 1 |
| Pe040 | Q865E8 | Peptide receptor | Melanocortin type 1 |
| Pe040 | P79166 | Peptide receptor | Melanocortin type 1 |
| Pe040 | Q865E9 | Peptide receptor | Melanocortin type 1 |
| Pe041 | P35370 | Peptide receptor | Opioid type X |
| Pe041 | P79292 | Peptide receptor | Opioid type X |
| Pe041 | P35377 | Peptide receptor | Opioid type X |
| Pe041 | P41146 | Peptide receptor | Opioid type X |
| Pe041 | P47748 | Peptide receptor | Opioid type X |
| Pe042 | Q2Y2P0 | Peptide receptor | C-C Chemokine type 1 |
| Pe042 | P51675 | Peptide receptor | C-C Chemokine type 1 |
| Pe042 | P51676 | Peptide receptor | C-C Chemokine type 1 |
| Pe042 | P32246 | Peptide receptor | C-C Chemokine type 1 |
| Pe042 | P56482 | Peptide receptor | C-C Chemokine type 1 |
| Pe043 | B1PHQ8 | Peptide receptor | Fmet-leu-phe |
| Pe043 | B9VR26 | Peptide receptor | Fmet-leu-phe |
| Pe043 | Q99788 | Peptide receptor | Fmet-leu-phe |
| Pe043 | P97468 | Peptide receptor | Fmet-leu-phe |
| Pe043 | O35786 | Peptide receptor | Fmet-leu-phe |
| Pe044 | Q28929 | Peptide receptor | Angiotensin type 2 |
| Pe044 | P35374 | Peptide receptor | Angiotensin type 2 |
| Pe044 | P50052 | Peptide receptor | Angiotensin type 2 |
| Pe044 | P35351 | Peptide receptor | Angiotensin type 2 |
| Pe044 | Q9Z0Z6 | Peptide receptor | Angiotensin type 2 |
| Pe045 | Q2KIP6 | Peptide receptor | Opioid type K |
| Pe045 | P41144 | Peptide receptor | Opioid type K |
| Pe045 | P34975 | Peptide receptor | Opioid type K |
| Pe045 | P41145 | Peptide receptor | Opioid type K |
| Pe045 | P33534 | Peptide receptor | Opioid type K |
| Pe046 | P79177 | Peptide receptor | Fmet-leu-phe |
| Pe046 | P79236 | Peptide receptor | Fmet-leu-phe |
| Pe046 | P79190 | Peptide receptor | Fmet-leu-phe |
| Pe046 | P25090 | Peptide receptor | Fmet-leu-phe |
| Pe046 | P79242 | Peptide receptor | Fmet-leu-phe |
| Pe047 | Q15761 | Peptide receptor | Neuropeptide Y type 5 |
| Pe047 | O62729 | Peptide receptor | Neuropeptide Y type 5 |
| Pe047 | O70342 | Peptide receptor | Neuropeptide Y type 5 |
| Pe047 | O97969 | Peptide receptor | Neuropeptide Y type 5 |
| Pe047 | Q63634 | Peptide receptor | Neuropeptide Y type 5 |
| Pe048 | P30873 | Peptide receptor | Somatostatin type 1 |
| Pe048 | P30872 | Peptide receptor | Somatostatin type 1 |
| Pe048 | P28646 | Peptide receptor | Somatostatin type 1 |
| Pe048 | Q49LX5 | Peptide receptor | Somatostatin type 1 |
| Pe049 | Q6BD04 | Peptide receptor | Kiss receptor (GPR54) |
| Pe049 | Q91V45 | Peptide receptor | Kiss receptor (GPR54) |
| Pe049 | Q969F8 | Peptide receptor | Kiss receptor (GPR54) |
| Pe049 | Q924U1 | Peptide receptor | Kiss receptor (GPR54) |
| Pe050 | O18793 | Peptide receptor | C-C Chemokine type 2 |
| Pe050 | P41597 | Peptide receptor | C-C Chemokine type 2 |
| Pe050 | P51683 | Peptide receptor | C-C Chemokine type 2 |
| Pe050 | O55193 | Peptide receptor | C-C Chemokine type 2 |
| Pe051 | Q9BDS8 | Peptide receptor | C-C Chemokine type 3 |
| Pe051 | P56483 | Peptide receptor | C-C Chemokine type 3 |
| Pe051 | P51677 | Peptide receptor | C-C Chemokine type 3 |
| Pe051 | P56492 | Peptide receptor | C-C Chemokine type 3 |
| Pe052 | Q9Z1S9 | Peptide receptor | Adrenocorticotropic hormone |
| Pe052 | P70115 | Peptide receptor | Adrenocorticotropic hormone |
| Pe052 | Q01718 | Peptide receptor | Adrenocorticotropic hormone |
| Pe052 | Q64326 | Peptide receptor | Adrenocorticotropic hormone |
| Pe053 | O97666 | Peptide receptor | APJ like type 1 |
| Pe053 | Q9JHG3 | Peptide receptor | APJ like type 1 |
| Pe053 | Q9WV08 | Peptide receptor | APJ like type 1 |
| Pe053 | P35414 | Peptide receptor | APJ like type 1 |
| Pe054 | Q4EW11 | Peptide receptor | Prolactin-releasing peptide (GPR10) |
| Pe054 | P49683 | Peptide receptor | Prolactin-releasing peptide (GPR10) |
| Pe054 | Q6VMN6 | Peptide receptor | Prolactin-releasing peptide (GPR10) |
| Pe054 | Q64121 | Peptide receptor | Prolactin-releasing peptide (GPR10) |
| Pe055 | Q8BZP8 | Peptide receptor | Vasotocin |
| Pe055 | Q6W5P4 | Peptide receptor | Vasotocin |
| Pe055 | P0C0L6 | Peptide receptor | Vasotocin |
| Pe055 | Q56H79 | Peptide receptor | Vasotocin |
| Pe056 | Q8BZ39 | Peptide receptor | Neuromedin U |
| Pe056 | Q9ESQ4 | Peptide receptor | Neuromedin U |
| Pe056 | Q58CW4 | Peptide receptor | Neuromedin U |
| Pe056 | Q9GZQ4 | Peptide receptor | Neuromedin U |
| Pe057 | P49681 | Peptide receptor | Somatostatin type 5 |
| Pe057 | P48145 | Peptide receptor | Somatostatin type 5 |
| Pe057 | Q8MJV3 | Peptide receptor | Somatostatin type 5 |
| Pe057 | Q56UD9 | Peptide receptor | Somatostatin type 5 |
| Pe058 | O60883 | Peptide receptor | GPR37 / endothelin B-like |
| Pe058 | Q9QYC5 | Peptide receptor | GPR37 / endothelin B-like |
| Pe058 | Q17QD8 | Peptide receptor | GPR37 / endothelin B-like |
| Pe058 | Q99JG2 | Peptide receptor | GPR37 / endothelin B-like |
| Pe059 | Q63645 | Peptide receptor | Proteinase-activated type 2 |
| Pe059 | Q2HJA4 | Peptide receptor | Proteinase-activated type 2 |
| Pe059 | P55086 | Peptide receptor | Proteinase-activated type 2 |
| Pe059 | P55085 | Peptide receptor | Proteinase-activated type 2 |
| Pe060 | Q864F6 | Peptide receptor | Melanocortin type 1 |
| Pe060 | Q864F4 | Peptide receptor | Melanocortin type 1 |
| Pe060 | Q864F5 | Peptide receptor | Melanocortin type 1 |
| Pe061 | Q66H29 | Peptide receptor | Melanin-concentrating hormone receptors |
| Pe061 | Q9UJ42 | Peptide receptor | Melanin-concentrating hormone receptors |
| Pe061 | Q3U3F9 | Peptide receptor | Melanin-concentrating hormone receptors |
| Pe062 | O55040 | Peptide receptor | Neuromedin U |
| Pe062 | Q9JJI5 | Peptide receptor | Neuromedin U |
| Pe062 | Q9HB89 | Peptide receptor | Neuromedin U |
| Pe063 | P56484 | Peptide receptor | C-C Chemokine type 8 |
| Pe063 | P51685 | Peptide receptor | C-C Chemokine type 8 |
| Pe063 | O97665 | Peptide receptor | C-C Chemokine type 8 |
| Pe064 | O95665 | Peptide receptor | Neurotensin type 2 |
| Pe064 | P70310 | Peptide receptor | Neurotensin type 2 |
| Pe064 | Q63384 | Peptide receptor | Neurotensin type 2 |
| Pe065 | Q9Z2J6 | Peptide receptor | Fmet-leu-phe |
| Pe065 | Q9Y5Y4 | Peptide receptor | Fmet-leu-phe |
| Pe065 | Q6XKD3 | Peptide receptor | Fmet-leu-phe |
| Pe066 | O08878 | Peptide receptor | Chemokine receptor-like 2 |
| Pe066 | Q8BMP4 | Peptide receptor | Chemokine receptor-like 2 |
| Pe066 | Q99527 | Peptide receptor | Chemokine receptor-like 2 |
| Pe067 | Q4VA82 | Peptide receptor | APJ like Non Vertebrate type 1 |
| Pe067 | Q2TAD5 | Peptide receptor | APJ like Non Vertebrate type 1 |
| Pe067 | P79960 | Peptide receptor | APJ like Non Vertebrate type 1 |
| Pe068 | P32248 | Peptide receptor | C-C Chemokine type 7 |
| Pe068 | P47774 | Peptide receptor | C-C Chemokine type 7 |
| Pe068 | Q5MD62 | Peptide receptor | C-C Chemokine type 7 |
| Pe069 | Q01727 | Peptide receptor | Melanocortin type 1 |
| Pe069 | Q80SS9 | Peptide receptor | Melanocortin type 1 |
| Pe069 | Q80SZ5 | Peptide receptor | Melanocortin type 1 |
| Pe070 | P30731 | Peptide receptor | GPR83 like |
| Pe070 | Q9TTQ9 | Peptide receptor | GPR83 like |
| Pe070 | Q9NYM4 | Peptide receptor | GPR83 like |
| Pe071 | Q7ZXJ7 | Peptide receptor | C-X-C Chemokine type 4 |
| Pe071 | Q9YGC3 | Peptide receptor | C-X-C Chemokine type 4 |
| Pe071 | Q07FZ4 | Peptide receptor | C-X-C Chemokine type 4 |
| Pe072 | Q96P65 | Peptide receptor | Orexigenic neuropeptide QRFP |
| Pe072 | P83858 | Peptide receptor | Orexigenic neuropeptide QRFP |
| Pe072 | P83861 | Peptide receptor | Orexigenic neuropeptide QRFP |
| Pe073 | Q99463 | Peptide receptor | Neuropeptide Y / peptide YY |
| Pe073 | Q61212 | Peptide receptor | Neuropeptide Y / peptide YY |
| Pe073 | P79217 | Peptide receptor | Neuropeptide Y / peptide YY |
| Pe074 | O88634 | Peptide receptor | Proteinase-activated type 4 |
| Pe074 | Q920E0 | Peptide receptor | Proteinase-activated type 4 |
| Pe074 | Q96RI0 | Peptide receptor | Proteinase-activated type 4 |
| Pe076 | P47211 | Peptide receptor | Galanin type 1 |
| Pe076 | P56479 | Peptide receptor | Galanin type 1 |
| Pe076 | Q62805 | Peptide receptor | Galanin type 1 |
| Pe077 | O00590 | Peptide receptor | C-C Chemokine type D6 |
| Pe077 | O08707 | Peptide receptor | C-C Chemokine type D6 |
| Pe077 | O09027 | Peptide receptor | C-C Chemokine type D6 |
| Pe078 | Q1WLP9 | Peptide receptor | C-C Chemokine type 9 |
| Pe078 | P51686 | Peptide receptor | C-C Chemokine type 9 |
| Pe078 | Q9WUT7 | Peptide receptor | C-C Chemokine type 9 |
| Pe079 | Q9XSD7 | Peptide receptor | C-C Chemokine type 2-like |
| Pe079 | O35457 | Peptide receptor | C-C Chemokine 102 |
| Pe079 | O00421 | Peptide receptor | C-C Chemokine type 2-like |
| Pe080 | P32745 | Peptide receptor | Somatostatin type 3 |
| Pe080 | P30935 | Peptide receptor | Somatostatin type 3 |
| Pe080 | P30936 | Peptide receptor | Somatostatin type 3 |
| Pe081 | P20789 | Peptide receptor | Neurotensin type 1 |
| Pe081 | O88319 | Peptide receptor | Neurotensin type 1 |
| Pe081 | P30989 | Peptide receptor | Neurotensin type 1 |
| Pe082 | Q8MJ88 | Peptide receptor | Melanin-concentrating hormone receptors |
| Pe082 | Q969V1 | Peptide receptor | Melanin-concentrating hormone receptors |
| Pe082 | Q8SQ54 | Peptide receptor | Melanin-concentrating hormone receptors |
| Pe083 | Q9MZV8 | Peptide receptor | Melanocortin type 5 |
| Pe083 | P56451 | Peptide receptor | Melanocortin type 5 |
| Pe083 | P41983 | Peptide receptor | Melanocortin type 5 |
| Pe084 | Q695P6 | Peptide receptor | C5a anaphylatoxin C5L2 |
| Pe084 | Q9P296 | Peptide receptor | C5a anaphylatoxin C5L2 |
| Pe084 | Q8BW93 | Peptide receptor | C5a anaphylatoxin C5L2 |
| Pe085 | Q9WU02 | Peptide receptor | Vasopressin type 1b |
| Pe085 | P47901 | Peptide receptor | Vasopressin type 1b |
| Pe085 | P48974 | Peptide receptor | Vasopressin type 1b |
| Pe086 | P33533 | Peptide receptor | Opioid type D |
| Pe086 | P41143 | Peptide receptor | Opioid type D |
| Pe086 | P32300 | Peptide receptor | Opioid type D |
| Pe087 | Q63447 | Peptide receptor | Neuropeptide Y type 4 |
| Pe087 | P50391 | Peptide receptor | Neuropeptide Y type 4 |
| Pe087 | Q61041 | Peptide receptor | Neuropeptide Y type 4 |
| Pe088 | P28336 | Peptide receptor | Neuromedin B receptor |
| Pe088 | P24053 | Peptide receptor | Neuromedin B receptor |
| Pe088 | O54799 | Peptide receptor | Neuromedin B receptor |
| Pe089 | Q04683 | Peptide receptor | C-X-C Chemokine type 5 |
| Pe089 | P32302 | Peptide receptor | C-X-C Chemokine type 5 |
| Pe089 | P34997 | Peptide receptor | C-X-C Chemokine type 5 |
| Pe090 | P51679 | Peptide receptor | C-C Chemokine type 4 |
| Pe090 | Q8MJW8 | Peptide receptor | C-C Chemokine type 4 |
| Pe090 | P51680 | Peptide receptor | C-C Chemokine type 4 |
| Pe091 | Q9QY42 | Peptide receptor | GPR37 / endothelin B-like |
| Pe091 | O15354 | Peptide receptor | GPR37 / endothelin B-like |
| Pe091 | Q9QYC6 | Peptide receptor | GPR37 / endothelin B-like |
| Pe092 | P34974 | Peptide receptor | Adrenocorticotropic hormone |
| Pe092 | Q9TU77 | Peptide receptor | Adrenocorticotropic hormone |
| Pe092 | Q8HYN8 | Peptide receptor | Adrenocorticotropic hormone |
| Pe093 | P43142 | Peptide receptor | Adrenomedullin (G10D) 1 |
| Pe093 | O15218 | Peptide receptor | Adrenomedullin (G10D) 1 |
| Pe093 | P31392 | Peptide receptor | Adrenomedullin (G10D) 1 |
| Pe094 | P49660 | Peptide receptor | Somatostatin type 4 |
| Pe094 | P30937 | Peptide receptor | Somatostatin type 4 |
| Pe094 | P31391 | Peptide receptor | Somatostatin type 4 |
| Pe095 | P35346 | Peptide receptor | Somatostatin type 5 |
| Pe095 | O08858 | Peptide receptor | Somatostatin type 5 |
| Pe095 | P30938 | Peptide receptor | Somatostatin type 5 |
| Pe096 | Q8CIM5 | Peptide receptor | Kiss receptor (GPR54) |
| Pe096 | Q9NQS5 | Peptide receptor | Kiss receptor (GPR54) |
| Pe096 | Q2KI97 | Peptide receptor | Kiss receptor (GPR54) |
| Pe097 | Q924H0 | Peptide receptor | Neuropeptide FF type 2 |
| Pe097 | Q9Y5X5 | Peptide receptor | Neuropeptide FF type 2 |
| Pe097 | Q9EQD2 | Peptide receptor | Neuropeptide FF type 2 |
| Pe098 | O88853 | Peptide receptor | Galanin type 3 |
| Pe098 | O60755 | Peptide receptor | Galanin type 3 |
| Pe098 | O88626 | Peptide receptor | Galanin type 3 |
| Pe099 | Q924I3 | Peptide receptor | C-C Chemokine type 11 |
| Pe099 | P35350 | Peptide receptor | C-C Chemokine type 11 |
| Pe099 | Q9NPB9 | Peptide receptor | C-C Chemokine type 11 |
| Pe100 | Q2KTE1 | Peptide receptor | C-X3-C Chemokine |
| Pe100 | A6QNL7 | Peptide receptor | C-X3-C Chemokine |
| Pe100 | P49238 | Peptide receptor | C-X3-C Chemokine |
| Pe101 | O88854 | Peptide receptor | Galanin type 2 |
| Pe101 | O43603 | Peptide receptor | Galanin type 2 |
| Pe101 | O08726 | Peptide receptor | Galanin type 2 |
| Pe102 | Q8VIH9 | Peptide receptor | Urotensin II |
| Pe102 | P49220 | Peptide receptor | Urotensin II |
| Pe102 | P49684 | Peptide receptor | Urotensin II |
| Pe103 | Q29154 | Peptide receptor | Melanocortin type 1 |
| Pe103 | Q6A155 | Peptide receptor | Melanocortin type 1 |
| Pe103 | O77616 | Peptide receptor | Melanocortin type 1 |
| Pe104 | Q49SQ3 | Peptide receptor | Chemokine receptor-like 1 |
| Pe104 | Q49SQ1 | Peptide receptor | Chemokine receptor-like 1 |
| Pe104 | Q49SQ2 | Peptide receptor | Chemokine receptor-like 1 |
| Pe105 | P70585 | Peptide receptor | Somatostatin type 5 |
| Pe105 | Q61121 | Peptide receptor | Somatostatin type 5 |
| Pe105 | Q15760 | Peptide receptor | Somatostatin type 5 |
| Pe106 | P52500 | Peptide receptor | Gastrin-releasing peptide receptor |
| Pe106 | P21729 | Peptide receptor | Gastrin-releasing peptide receptor |
| Pe106 | P30550 | Peptide receptor | Gastrin-releasing peptide receptor |
| Pe107 | Q6UNA4 | Peptide receptor | C5a anaphylatoxin |
| Pe107 | P0C7U5 | Peptide receptor | C5a anaphylatoxin |
| Pe108 | P0C7U4 | Peptide receptor | C5a anaphylatoxin |
| Pe108 | Q2WED0 | Peptide receptor | C5a anaphylatoxin |
| Pe109 | Q86VZ1 | Peptide receptor | Proteinase-activated type 2 |
| Pe109 | Q5ZI82 | Peptide receptor | Proteinase-activated type 2 |
| Pe110 | O75388 | Peptide receptor | Fmet-leu-phe |
| Pe110 | Q8NGA4 | Peptide receptor | Fmet-leu-phe |
| Pe111 | Q9R0M1 | Peptide receptor | XC Chemokine |
| Pe111 | P46094 | Peptide receptor | XC Chemokine |
| Pe112 | P0C5I1 | Peptide receptor | GPR25 |
| Pe112 | O00155 | Peptide receptor | GPR25 |
| Pe113 | P46092 | Peptide receptor | C-C Chemokine type 10 |
| Pe113 | Q9JL21 | Peptide receptor | C-C Chemokine type 10 |
| Pe114 | Q8MJV2 | Peptide receptor | Somatostatin type 5 |
| Pe114 | P48146 | Peptide receptor | Somatostatin type 5 |
| Pe115 | Q8TDU9 | Peptide receptor | Relaxin-3 type 2 |
| Pe115 | Q7TQP4 | Peptide receptor | Relaxin-3 type 2 |
| Pe116 | Q08520 | Peptide receptor | C-C Chemokine type 8 |
| Pe116 | P32229 | Peptide receptor | C-C Chemokine type 8 |
| Pe117 | Q75ZH0 | Peptide receptor | C-C Chemokine type 2-like |
| Pe117 | Q0II78 | Peptide receptor | C-C Chemokine type 2-like |
| Pe118 | A4FUQ5 | Peptide receptor | Fmet-leu-phe |
| Pe118 | O88537 | Peptide receptor | Fmet-leu-phe |
| Pe119 | Q6NV75 | Peptide receptor | Melanin-concentrating hormone receptors |
| Pe119 | Q8K0Z9 | Peptide receptor | Melanin-concentrating hormone receptors |
| Pe120 | B3G515 | Peptide receptor | Chemokine receptor-like 2 |
| Pe120 | B0F9W3 | Peptide receptor | Chemokine receptor-like 2 |
| Pe121 | Q16538 | Peptide receptor | Melanin-concentrating hormone receptors |
| Pe121 | Q3UN16 | Peptide receptor | Melanin-concentrating hormone receptors |
| Pe122 | Q71MR7 | Peptide receptor | Fmet-leu-phe |
| Pe122 | Q3SXG2 | Peptide receptor | Fmet-leu-phe |
| Pe123 | P33032 | Peptide receptor | Melanocortin type 5 |
| Pe123 | Q9TT23 | Peptide receptor | Melanocortin type 5 |
| Pe124 | P32303 | Peptide receptor | Angiotensin type 1 |
| Pe124 | P35373 | Peptide receptor | Angiotensin type 1 |
| Pe125 | P51684 | Peptide receptor | C-C Chemokine type 6 |
| Pe125 | O54689 | Peptide receptor | C-C Chemokine type 6 |
| Pe126 | Q8HYC3 | Peptide receptor | Urotensin II |
| Pe126 | Q9UKP6 | Peptide receptor | Urotensin II |
| Pe127 | O88416 | Peptide receptor | Chemokine receptor-like 1 |
| Pe127 | Q49SP8 | Peptide receptor | Chemokine receptor-like 1 |
| Pe128 | P35343 | Peptide receptor | Interleukin-8 type B |
| Pe128 | P35407 | Peptide receptor | Interleukin-8 type B |
| Pe129 | P35411 | Peptide receptor | C-X3-C Chemokine |
| Pe129 | Q9Z0D9 | Peptide receptor | C-X3-C Chemokine |
| Pe130 | P41149 | Peptide receptor | Melanocortin type 5 |
| Pe130 | P35345 | Peptide receptor | Melanocortin type 5 |
| Pe131 | O08790 | Peptide receptor | Fmet-leu-phe |
| Pe131 | O88536 | Peptide receptor | Fmet-leu-phe |
| Pe132 | P97583 | Peptide receptor | Bradykinin type B1 |
| Pe132 | Q61125 | Peptide receptor | Bradykinin type B1 |
| Pe133 | A0T2N3 | Peptide receptor | APJ like 3 |
| Pe133 | Q7SZP9 | Peptide receptor | APJ like 3 |
| Pe134 | Q9EP86 | Peptide receptor | Neuropeptide FF type 1 |
| Pe134 | Q9GZQ6 | Peptide receptor | Neuropeptide FF type 1 |
| Pe135 | Q864F8 | Peptide receptor | Melanocortin type 1 |
| Pe135 | Q864F7 | Peptide receptor | Melanocortin type 1 |
| Pe136 | Q96P67 | Peptide receptor | Angiotensin type 1 |
| Pe136 | Q8BZR0 | Peptide receptor | Angiotensin type 1 |
| Pe137 | O08675 | Peptide receptor | Proteinase-activated type 3 |
| Pe137 | Q920E1 | Peptide receptor | Proteinase-activated type 3 |
| Pe138 | P32244 | Peptide receptor | Melanocortin type 3 |
| Pe138 | P33033 | Peptide receptor | Melanocortin type 3 |
| Pe139 | Q9NSD7 | Peptide receptor | Relaxin-3 type 1 |
| Pe139 | Q8BGE9 | Peptide receptor | Relaxin-3 type 1 |
| Pe140 | Q863H8 | Peptide receptor | Duffy antigen |
| Pe140 | Q9GLX0 | Peptide receptor | Duffy antigen |
| Pe141 | P70612 | Peptide receptor | Interleukin-8 type A |
| Pe141 | Q810W6 | Peptide receptor | Interleukin-8 type A |
| Pe142 | Q58D85 | Peptide receptor | Proteinase-activated type 3 |
| Pe142 | O00254 | Peptide receptor | Proteinase-activated type 3 |
| Pe143 | P30993 | Peptide receptor | C5a anaphylatoxin |
| Pe143 | P97520 | Peptide receptor | C5a anaphylatoxin |
| Pe144 | Q29J90 | Peptide receptor | Melanin-concentrating hormone receptors |
| Pe144 | Q9W534 | Peptide receptor | Melanin-concentrating hormone receptors |
| Pe145 | Q8BXS7 | Peptide receptor | Melanin-concentrating hormone receptors |
| Pe145 | Q8TDT2 | Peptide receptor | Melanin-concentrating hormone receptors |
| Pi001 | P70205 | Pituitary adenylate cyclase-activating polypeptide type I receptor | PACAP |
| Pi001 | P41586 | Pituitary adenylate cyclase-activating polypeptide type I receptor | PACAP |
| Pi001 | Q29627 | Pituitary adenylate cyclase-activating polypeptide type I receptor | PACAP |
| Pi001 | P32215 | Pituitary adenylate cyclase-activating polypeptide type I receptor | PACAP |
| Pl001 | P21556 | Platelet-activating factor receptor | Platelet activating factor |
| Pl001 | Q9TTY5 | Platelet-activating factor receptor | Platelet activating factor |
| Pl001 | Q62035 | Platelet-activating factor receptor | Platelet activating factor |
| Pl001 | Q9XSD4 | Platelet-activating factor receptor | Platelet activating factor |
| Pl001 | P25105 | Platelet-activating factor receptor | Platelet activating factor |
| Pl001 | Q9GK76 | Platelet-activating factor receptor | Platelet activating factor |
| Pl001 | P46002 | Platelet-activating factor receptor | Platelet activating factor |
| Pr001 | P43114 | Prostanoid receptor | Prostaglandin E2 subtype EP4 |
| Pr001 | P32240 | Prostanoid receptor | Prostaglandin E2 subtype EP4 |
| Pr001 | Q95KZ0 | Prostanoid receptor | Prostaglandin E2 subtype EP4 |
| Pr001 | Q8MJ08 | Prostanoid receptor | Prostaglandin E2 subtype EP4 |
| Pr001 | P35408 | Prostanoid receptor | Prostaglandin E2 subtype EP4 |
| Pr001 | Q28691 | Prostanoid receptor | Prostaglandin E2 subtype EP4 |
| Pr002 | P43115 | Prostanoid receptor | Prostaglandin E2 subtype EP3 |
| Pr002 | P50131 | Prostanoid receptor | Prostaglandin E2 subtype EP3 |
| Pr002 | P46069 | Prostanoid receptor | Prostaglandin E2 subtype EP3 |
| Pr002 | P34979 | Prostanoid receptor | Prostaglandin E2 subtype EP3 |
| Pr002 | P34980 | Prostanoid receptor | Prostaglandin E2 subtype EP3 |
| Pr002 | P30557 | Prostanoid receptor | Prostaglandin E2 subtype EP3 |
| Pr003 | P43088 | Prostanoid receptor | Prostaglandin F2-alpha |
| Pr003 | P37289 | Prostanoid receptor | Prostaglandin F2-alpha |
| Pr003 | P43117 | Prostanoid receptor | Prostaglandin F2-alpha |
| Pr003 | P43118 | Prostanoid receptor | Prostaglandin F2-alpha |
| Pr003 | Q28905 | Prostanoid receptor | Prostaglandin F2-alpha |
| Pr004 | P35375 | Prostanoid receptor | Prostaglandin E2 subtype EP1 |
| Pr004 | P34995 | Prostanoid receptor | Prostaglandin E2 subtype EP1 |
| Pr004 | Q9BGL8 | Prostanoid receptor | Prostaglandin E2 subtype EP1 |
| Pr004 | P70597 | Prostanoid receptor | Prostaglandin E2 subtype EP1 |
| Pr005 | Q62928 | Prostanoid receptor | Prostaglandin E2/D2 subtype EP2 |
| Pr005 | Q62053 | Prostanoid receptor | Prostaglandin E2/D2 subtype EP2 |
| Pr005 | P43116 | Prostanoid receptor | Prostaglandin E2/D2 subtype EP2 |
| Pr005 | Q9XT82 | Prostanoid receptor | Prostaglandin E2/D2 subtype EP2 |
| Pr006 | P79393 | Prostanoid receptor | Prostacyclin |
| Pr006 | P43119 | Prostanoid receptor | Prostacyclin |
| Pr006 | P43252 | Prostanoid receptor | Prostacyclin |
| Pr006 | P43253 | Prostanoid receptor | Prostacyclin |
| Pr007 | Q95125 | Prostanoid receptor | Thromboxane |
| Pr007 | P21731 | Prostanoid receptor | Thromboxane |
| Pr007 | P56486 | Prostanoid receptor | Thromboxane |
| Pr008 | O35932 | Prostanoid receptor | Prostaglandin E2/D2 subtype EP2 |
| Pr008 | Q9R261 | Prostanoid receptor | Prostaglandin E2/D2 subtype EP2 |
| Pr008 | P70263 | Prostanoid receptor | Prostaglandin E2/D2 subtype EP2 |
| Pr009 | A5D7K8 | Prostanoid receptor | Prostaglandin E2/D2 subtype EP2 |
| Pr009 | Q13258 | Prostanoid receptor | Prostaglandin E2/D2 subtype EP2 |
| Pr010 | P34978 | Prostanoid receptor | Thromboxane |
| Pr010 | P30987 | Prostanoid receptor | Thromboxane |
| Rh001 | P56515 | (Rhod)opsin receptor | Vertebrate blue/green opsin |
| Rh001 | O93441 | (Rhod)opsin receptor | Vertebrate blue/green opsin |
| Rh001 | Q68J47 | (Rhod)opsin receptor | Vertebrate blue/green opsin |
| Rh001 | P28681 | (Rhod)opsin receptor | Vertebrate blue/green opsin |
| Rh001 | Q9YGZ4 | (Rhod)opsin receptor | Vertebrate blue/green opsin |
| Rh001 | P79809 | (Rhod)opsin receptor | Vertebrate blue/green opsin |
| Rh001 | P52202 | (Rhod)opsin receptor | Vertebrate blue/green opsin |
| Rh001 | Q9YH01 | (Rhod)opsin receptor | Vertebrate blue/green opsin |
| Rh001 | P79902 | (Rhod)opsin receptor | Vertebrate blue/green opsin |
| Rh001 | P79911 | (Rhod)opsin receptor | Vertebrate blue/green opsin |
| Rh001 | Q9YH02 | (Rhod)opsin receptor | Vertebrate blue/green opsin |
| Rh001 | Q9YH05 | (Rhod)opsin receptor | Vertebrate blue/green opsin |
| Rh001 | P32309 | (Rhod)opsin receptor | Vertebrate blue/green opsin |
| Rh001 | Q9YGZ0 | (Rhod)opsin receptor | Vertebrate blue/green opsin |
| Rh001 | P02699 | (Rhod)opsin receptor | Vertebrate blue/green opsin |
| Rh001 | P79808 | (Rhod)opsin receptor | Vertebrate blue/green opsin |
| Rh001 | P79848 | (Rhod)opsin receptor | Vertebrate blue/green opsin |
| Rh001 | Q6W3E1 | (Rhod)opsin receptor | Vertebrate blue/green opsin |
| Rh001 | P51488 | (Rhod)opsin receptor | Vertebrate blue/green opsin |
| Rh001 | P02700 | (Rhod)opsin receptor | Vertebrate blue/green opsin |
| Rh001 | Q9YGZ7 | (Rhod)opsin receptor | Vertebrate blue/green opsin |
| Rh001 | O93459 | (Rhod)opsin receptor | Vertebrate blue/green opsin |
| Rh001 | P15409 | (Rhod)opsin receptor | Vertebrate blue/green opsin |
| Rh001 | P79812 | (Rhod)opsin receptor | Vertebrate blue/green opsin |
| Rh001 | O62793 | (Rhod)opsin receptor | Vertebrate blue/green opsin |
| Rh001 | P56516 | (Rhod)opsin receptor | Vertebrate blue/green opsin |
| Rh001 | P32308 | (Rhod)opsin receptor | Vertebrate blue/green opsin |
| Rh001 | P79756 | (Rhod)opsin receptor | Vertebrate blue/green opsin |
| Rh001 | Q95KU1 | (Rhod)opsin receptor | Vertebrate blue/green opsin |
| Rh001 | Q9YGZ2 | (Rhod)opsin receptor | Vertebrate blue/green opsin |
| Rh001 | Q98980 | (Rhod)opsin receptor | Vertebrate blue/green opsin |
| Rh001 | O18766 | (Rhod)opsin receptor | Vertebrate blue/green opsin |
| Rh001 | P41591 | (Rhod)opsin receptor | Vertebrate blue/green opsin |
| Rh001 | P35359 | (Rhod)opsin receptor | Vertebrate blue/green opsin |
| Rh001 | Q9YGZ8 | (Rhod)opsin receptor | Vertebrate blue/green opsin |
| Rh001 | P22328 | (Rhod)opsin receptor | Vertebrate blue/green opsin |
| Rh001 | O42268 | (Rhod)opsin receptor | Vertebrate blue/green opsin |
| Rh001 | P51470 | (Rhod)opsin receptor | Vertebrate blue/green opsin |
| Rh001 | O62798 | (Rhod)opsin receptor | Vertebrate blue/green opsin |
| Rh001 | Q8HY69 | (Rhod)opsin receptor | Vertebrate blue/green opsin |
| Rh001 | Q9YGZ6 | (Rhod)opsin receptor | Vertebrate blue/green opsin |
| Rh001 | Q9YGZ9 | (Rhod)opsin receptor | Vertebrate blue/green opsin |
| Rh001 | P87369 | (Rhod)opsin receptor | Vertebrate blue/green opsin |
| Rh001 | P31355 | (Rhod)opsin receptor | Vertebrate blue/green opsin |
| Rh001 | O42466 | (Rhod)opsin receptor | Vertebrate blue/green opsin |
| Rh001 | O62795 | (Rhod)opsin receptor | Vertebrate blue/green opsin |
| Rh001 | Q9YH03 | (Rhod)opsin receptor | Vertebrate blue/green opsin |
| Rh001 | O42604 | (Rhod)opsin receptor | Vertebrate blue/green opsin |
| Rh001 | P35403 | (Rhod)opsin receptor | Vertebrate blue/green opsin |
| Rh001 | P29403 | (Rhod)opsin receptor | Vertebrate blue/green opsin |
| Rh001 | Q90214 | (Rhod)opsin receptor | Vertebrate blue/green opsin |
| Rh001 | O62791 | (Rhod)opsin receptor | Vertebrate blue/green opsin |
| Rh001 | Q9YH00 | (Rhod)opsin receptor | Vertebrate blue/green opsin |
| Rh001 | O62796 | (Rhod)opsin receptor | Vertebrate blue/green opsin |
| Rh001 | Q90215 | (Rhod)opsin receptor | Vertebrate blue/green opsin |
| Rh001 | P79807 | (Rhod)opsin receptor | Vertebrate blue/green opsin |
| Rh001 | O62794 | (Rhod)opsin receptor | Vertebrate blue/green opsin |
| Rh001 | P79901 | (Rhod)opsin receptor | Vertebrate blue/green opsin |
| Rh001 | P79863 | (Rhod)opsin receptor | Vertebrate blue/green opsin |
| Rh001 | Q9YGZ5 | (Rhod)opsin receptor | Vertebrate blue/green opsin |
| Rh001 | Q9DGG4 | (Rhod)opsin receptor | Vertebrate blue/green opsin |
| Rh001 | Q28886 | (Rhod)opsin receptor | Vertebrate blue/green opsin |
| Rh001 | P79798 | (Rhod)opsin receptor | Vertebrate blue/green opsin |
| Rh001 | P08100 | (Rhod)opsin receptor | Vertebrate blue/green opsin |
| Rh001 | P56514 | (Rhod)opsin receptor | Vertebrate blue/green opsin |
| Rh001 | P41590 | (Rhod)opsin receptor | Vertebrate blue/green opsin |
| Rh001 | P79914 | (Rhod)opsin receptor | Vertebrate blue/green opsin |
| Rh001 | O13227 | (Rhod)opsin receptor | Vertebrate blue/green opsin |
| Rh001 | Q9YGY9 | (Rhod)opsin receptor | Vertebrate blue/green opsin |
| Rh001 | Q9YGZ3 | (Rhod)opsin receptor | Vertebrate blue/green opsin |
| Rh001 | Q9YGZ1 | (Rhod)opsin receptor | Vertebrate blue/green opsin |
| Rh001 | O62792 | (Rhod)opsin receptor | Vertebrate blue/green opsin |
| Rh001 | P49912 | (Rhod)opsin receptor | Vertebrate blue/green opsin |
| Rh001 | P51489 | (Rhod)opsin receptor | Vertebrate blue/green opsin |
| Rh001 | Q90245 | (Rhod)opsin receptor | Vertebrate blue/green opsin |
| Rh001 | Q769E8 | (Rhod)opsin receptor | Vertebrate blue/green opsin |
| Rh001 | Q9YH04 | (Rhod)opsin receptor | Vertebrate blue/green opsin |
| Rh001 | P79898 | (Rhod)opsin receptor | Vertebrate blue/green opsin |
| Rh001 | P79903 | (Rhod)opsin receptor | Vertebrate blue/green opsin |
| Rh002 | P04000 | (Rhod)opsin receptor | Vertebrate red opsin |
| Rh002 | P34989 | (Rhod)opsin receptor | Vertebrate red opsin |
| Rh002 | Q9W6A7 | (Rhod)opsin receptor | Vertebrate red opsin |
| Rh002 | P87367 | (Rhod)opsin receptor | Vertebrate red opsin |
| Rh002 | O35478 | (Rhod)opsin receptor | Vertebrate red opsin |
| Rh002 | Q9BGI7 | (Rhod)opsin receptor | Vertebrate red opsin |
| Rh002 | P22330 | (Rhod)opsin receptor | Vertebrate red opsin |
| Rh002 | P41592 | (Rhod)opsin receptor | Vertebrate red opsin |
| Rh002 | Q95170 | (Rhod)opsin receptor | Vertebrate red opsin |
| Rh002 | Q8AYN0 | (Rhod)opsin receptor | Vertebrate red opsin |
| Rh002 | P22331 | (Rhod)opsin receptor | Vertebrate red opsin |
| Rh002 | O18912 | (Rhod)opsin receptor | Vertebrate red opsin |
| Rh002 | P22329 | (Rhod)opsin receptor | Vertebrate red opsin |
| Rh002 | O18911 | (Rhod)opsin receptor | Vertebrate red opsin |
| Rh002 | O18910 | (Rhod)opsin receptor | Vertebrate red opsin |
| Rh002 | O18913 | (Rhod)opsin receptor | Vertebrate red opsin |
| Rh002 | O12948 | (Rhod)opsin receptor | Vertebrate red opsin |
| Rh002 | P04001 | (Rhod)opsin receptor | Vertebrate red opsin |
| Rh002 | Q9R024 | (Rhod)opsin receptor | Vertebrate red opsin |
| Rh002 | O18914 | (Rhod)opsin receptor | Vertebrate red opsin |
| Rh002 | O35599 | (Rhod)opsin receptor | Vertebrate red opsin |
| Rh002 | O35476 | (Rhod)opsin receptor | Vertebrate red opsin |
| Rh002 | P22332 | (Rhod)opsin receptor | Vertebrate red opsin |
| Rh002 | P32313 | (Rhod)opsin receptor | Vertebrate red opsin |
| Rh002 | P35358 | (Rhod)opsin receptor | Vertebrate red opsin |
| Rh003 | O42431 | (Rhod)opsin receptor | Vertebrate blue/green opsin |
| Rh003 | O42330 | (Rhod)opsin receptor | Vertebrate blue/green opsin |
| Rh003 | O42327 | (Rhod)opsin receptor | Vertebrate blue/green opsin |
| Rh003 | O42307 | (Rhod)opsin receptor | Vertebrate blue/green opsin |
| Rh003 | O42301 | (Rhod)opsin receptor | Vertebrate blue/green opsin |
| Rh003 | O42328 | (Rhod)opsin receptor | Vertebrate blue/green opsin |
| Rh003 | Q90373 | (Rhod)opsin receptor | Vertebrate blue/green opsin |
| Rh003 | O42294 | (Rhod)opsin receptor | Vertebrate blue/green opsin |
| Rh003 | O42451 | (Rhod)opsin receptor | Vertebrate blue/green opsin |
| Rh003 | O42300 | (Rhod)opsin receptor | Vertebrate blue/green opsin |
| Rh003 | O42427 | (Rhod)opsin receptor | Vertebrate blue/green opsin |
| Rh003 | O42452 | (Rhod)opsin receptor | Vertebrate blue/green opsin |
| Rh004 | Q9W6A5 | (Rhod)opsin receptor | Vertebrate blue/green opsin |
| Rh004 | Q8AYM8 | (Rhod)opsin receptor | Vertebrate blue/green opsin |
| Rh004 | Q8AYM7 | (Rhod)opsin receptor | Vertebrate blue/green opsin |
| Rh004 | P87366 | (Rhod)opsin receptor | Vertebrate blue/green opsin |
| Rh004 | P32312 | (Rhod)opsin receptor | Vertebrate blue/green opsin |
| Rh004 | P51474 | (Rhod)opsin receptor | Vertebrate blue/green opsin |
| Rh004 | P51471 | (Rhod)opsin receptor | Vertebrate blue/green opsin |
| Rh004 | P35357 | (Rhod)opsin receptor | Vertebrate blue/green opsin |
| Rh004 | P28683 | (Rhod)opsin receptor | Vertebrate blue/green opsin |
| Rh004 | Q9W6A6 | (Rhod)opsin receptor | Vertebrate blue/green opsin |
| Rh004 | P32311 | (Rhod)opsin receptor | Vertebrate blue/green opsin |
| Rh005 | O16019 | (Rhod)opsin receptor | Rhodopsin Arthropod short wavelength 2 |
| Rh005 | O18486 | (Rhod)opsin receptor | Rhodopsin Arthropod short wavelength 2 |
| Rh005 | O16018 | (Rhod)opsin receptor | Rhodopsin Arthropod short wavelength 2 |
| Rh005 | O16017 | (Rhod)opsin receptor | Rhodopsin Arthropod short wavelength 2 |
| Rh005 | P35356 | (Rhod)opsin receptor | Rhodopsin Arthropod short wavelength 2 |
| Rh005 | O18312 | (Rhod)opsin receptor | Rhodopsin Arthropod short wavelength 2 |
| Rh005 | O18315 | (Rhod)opsin receptor | Rhodopsin Arthropod short wavelength 2 |
| Rh005 | O18481 | (Rhod)opsin receptor | Rhodopsin Arthropod short wavelength 2 |
| Rh005 | O18485 | (Rhod)opsin receptor | Rhodopsin Arthropod short wavelength 2 |
| Rh005 | O16020 | (Rhod)opsin receptor | Rhodopsin Arthropod short wavelength 2 |
| Rh006 | O57605 | (Rhod)opsin receptor | Vertebrate short wavelength opsin |
| Rh006 | P03999 | (Rhod)opsin receptor | Vertebrate short wavelength opsin |
| Rh006 | P51490 | (Rhod)opsin receptor | Vertebrate short wavelength opsin |
| Rh006 | P60573 | (Rhod)opsin receptor | Vertebrate short wavelength opsin |
| Rh006 | P51491 | (Rhod)opsin receptor | Vertebrate short wavelength opsin |
| Rh006 | Q63652 | (Rhod)opsin receptor | Vertebrate short wavelength opsin |
| Rh006 | P28684 | (Rhod)opsin receptor | Vertebrate short wavelength opsin |
| Rh006 | P51473 | (Rhod)opsin receptor | Vertebrate short wavelength opsin |
| Rh006 | O13092 | (Rhod)opsin receptor | Vertebrate short wavelength opsin |
| Rh006 | P60015 | (Rhod)opsin receptor | Vertebrate short wavelength opsin |
| Rh007 | Q4R1I4 | (Rhod)opsin receptor | Cephalochordata melanopsin |
| Rh007 | Q2KNE5 | (Rhod)opsin receptor | Vertebrate melanopsin |
| Rh007 | Q9UHM6 | (Rhod)opsin receptor | Vertebrate melanopsin |
| Rh007 | Q5YKK9 | (Rhod)opsin receptor | Vertebrate melanopsin |
| Rh007 | Q8R456 | (Rhod)opsin receptor | Vertebrate melanopsin |
| Rh007 | Q9QXZ9 | (Rhod)opsin receptor | Vertebrate melanopsin |
| Rh007 | Q5XXP2 | (Rhod)opsin receptor | Vertebrate melanopsin |
| Rh008 | Q17053 | (Rhod)opsin receptor | Rhodopsin Arthropod short wavelength 2 |
| Rh008 | Q95YI3 | (Rhod)opsin receptor | Rhodopsin Arthropod short wavelength 2 |
| Rh008 | P35362 | (Rhod)opsin receptor | Rhodopsin Arthropod short wavelength 2 |
| Rh008 | O01668 | (Rhod)opsin receptor | Rhodopsin Arthropod short wavelength 2 |
| Rh008 | Q17296 | (Rhod)opsin receptor | Rhodopsin Arthropod short wavelength 2 |
| Rh008 | O02464 | (Rhod)opsin receptor | Rhodopsin Arthropod short wavelength 2 |
| Rh008 | Q94741 | (Rhod)opsin receptor | Rhodopsin Arthropod short wavelength 2 |
| Rh009 | P04950 | (Rhod)opsin receptor | Rhodopsin Arthropod short wavelength 1 |
| Rh009 | O61303 | (Rhod)opsin receptor | Rhodopsin Arthropod short wavelength 1 |
| Rh009 | P08255 | (Rhod)opsin receptor | Rhodopsin Arthropod short wavelength 1 |
| Rh009 | P29404 | (Rhod)opsin receptor | Rhodopsin Arthropod short wavelength 1 |
| Rh009 | P17646 | (Rhod)opsin receptor | Rhodopsin Arthropod short wavelength 1 |
| Rh009 | P28680 | (Rhod)opsin receptor | Rhodopsin Arthropod short wavelength 1 |
| Rh009 | O02465 | (Rhod)opsin receptor | Rhodopsin Arthropod short wavelength 1 |
| Rh010 | O16005 | (Rhod)opsin receptor | Vertebrate opsin |
| Rh010 | Q17094 | (Rhod)opsin receptor | Vertebrate opsin |
| Rh010 | P31356 | (Rhod)opsin receptor | Vertebrate opsin |
| Rh010 | P24603 | (Rhod)opsin receptor | Vertebrate opsin |
| Rh010 | P09241 | (Rhod)opsin receptor | Vertebrate opsin |
| Rh010 | O15973 | (Rhod)opsin receptor | Rhodopsin 6 1 |
| Rh011 | P51472 | (Rhod)opsin receptor | Vertebrate blue/green opsin |
| Rh011 | Q9W6A8 | (Rhod)opsin receptor | Vertebrate blue/green opsin |
| Rh011 | P32310 | (Rhod)opsin receptor | Vertebrate blue/green opsin |
| Rh011 | P28682 | (Rhod)opsin receptor | Vertebrate blue/green opsin |
| Rh011 | P87365 | (Rhod)opsin receptor | Vertebrate blue/green opsin |
| Rh012 | O96107 | (Rhod)opsin receptor | Rhodopsin Arthropod short wavelength 1 |
| Rh012 | P91657 | (Rhod)opsin receptor | Rhodopsin Arthropod short wavelength 1 |
| Rh012 | Q26495 | (Rhod)opsin receptor | Rhodopsin Arthropod short wavelength 1 |
| Rh012 | P90680 | (Rhod)opsin receptor | Rhodopsin Arthropod short wavelength 1 |
| Rh013 | Q7T3Q7 | (Rhod)opsin receptor | Vertebrate ancient (long) opsin |
| Rh013 | O42490 | (Rhod)opsin receptor | Vertebrate P-opsin |
| Rh013 | O13018 | (Rhod)opsin receptor | Vertebrate ancient (long) opsin |
| Rh014 | Q9Z2B3 | (Rhod)opsin receptor | Vertebrate red opsin |
| Rh014 | P47804 | (Rhod)opsin receptor | Vertebrate red opsin |
| Rh014 | P47803 | (Rhod)opsin receptor | Vertebrate red opsin |
| Rh015 | Q90309 | (Rhod)opsin receptor | Vertebrate short wavelength opsin |
| Rh015 | P87368 | (Rhod)opsin receptor | Vertebrate short wavelength opsin |
| Rh015 | Q9W6A9 | (Rhod)opsin receptor | Vertebrate short wavelength opsin |
| Rh016 | P28678 | (Rhod)opsin receptor | Rhodopsin Arthropod short wavelength 2 |
| Rh016 | P06002 | (Rhod)opsin receptor | Rhodopsin Arthropod short wavelength 2 |
| Rh016 | P22269 | (Rhod)opsin receptor | Rhodopsin Arthropod short wavelength 2 |
| Rh017 | P51476 | (Rhod)opsin receptor | Vertebrate Pinopsin |
| Rh017 | O42266 | (Rhod)opsin receptor | Vertebrate Parapinopsin |
| Rh017 | P51475 | (Rhod)opsin receptor | Vertebrate Pinopsin |
| Rh018 | Q4U4D2 | (Rhod)opsin receptor | Vertebrate melanopsin |
| Rh018 | O57422 | (Rhod)opsin receptor | Vertebrate melanopsin |
| Rh019 | Q804X9 | (Rhod)opsin receptor | Vertebrate melanopsin |
| Rh019 | Q804Q2 | (Rhod)opsin receptor | Vertebrate melanopsin |
| Rh020 | O14718 | (Rhod)opsin receptor | Vertebrate Peropsin |
| Rh020 | O35214 | (Rhod)opsin receptor | Vertebrate Peropsin |
| Rh021 | P35361 | (Rhod)opsin receptor | Rhodopsin Arthropod short wavelength 2 |
| Rh021 | P35360 | (Rhod)opsin receptor | Rhodopsin Arthropod short wavelength 2 |
| Rh022 | P28679 | (Rhod)opsin receptor | Rhodopsin Arthropod short wavelength 2 |
| Rh022 | P08099 | (Rhod)opsin receptor | Rhodopsin Arthropod short wavelength 2 |
| Rh023 | Q6U736 | (Rhod)opsin receptor | Vertebrate opsin 5 |
| Rh023 | Q6VZZ7 | (Rhod)opsin receptor | Vertebrate opsin 5 |
| Rh024 | Q9H1Y3 | (Rhod)opsin receptor | Vertebrate opsin type 3 |
| Rh024 | Q9WUK7 | (Rhod)opsin receptor | Vertebrate opsin type 3 |
| Rh025 | Q1JPS6 | (Rhod)opsin receptor | Vertebrate melanopsin |
| Rh025 | Q6XL69 | (Rhod)opsin receptor | Vertebrate melanopsin |
| Rh026 | Q25157 | (Rhod)opsin receptor | Rhodopsin 5 3 |
| Rh026 | Q25158 | (Rhod)opsin receptor | Rhodopsin 5 3 |
| Se001 | P47872 | Secretin receptor | Secretin |
| Se001 | O46502 | Secretin receptor | Secretin |
| Se001 | P23811 | Secretin receptor | Secretin |
| Se001 | Q5FWI2 | Secretin receptor | Secretin |
| T1001 | Q717C2 | Taste receptor type 1 | Taste 1 |
| T1001 | Q717C1 | Taste receptor type 1 | Taste 1 |
| T1001 | Q7RTX0 | Taste receptor type 1 | Taste 1 |
| T1001 | Q49HH9 | Taste receptor type 1 | Taste 23 |
| T1001 | Q925D8 | Taste receptor type 1 | Taste 21 |
| T1001 | Q923K1 | Taste receptor type 1 | Taste 21 |
| T1001 | Q49KI5 | Taste receptor type 1 | Taste 22 |
| T2001 | P59539 | Taste receptor type 2 | Taste receptors T2R |
| T2001 | P59540 | Taste receptor type 2 | Taste receptors T2R |
| T2001 | Q646G0 | Taste receptor type 2 | Taste receptors T2R |
| T2001 | Q646F9 | Taste receptor type 2 | Taste receptors T2R |
| T2001 | Q646E1 | Taste receptor type 2 | Taste receptors T2R |
| T2001 | Q645Z7 | Taste receptor type 2 | Taste receptors T2R |
| T2001 | Q645T4 | Taste receptor type 2 | Taste receptors T2R |
| T2001 | Q5Y4Z8 | Taste receptor type 2 | Taste receptors T2R |
| T2001 | Q5Y4Y8 | Taste receptor type 2 | Taste receptors T2R |
| T2002 | P59533 | Taste receptor type 2 | Taste receptors T2R |
| T2002 | Q697L6 | Taste receptor type 2 | Taste receptors T2R |
| T2002 | Q697L5 | Taste receptor type 2 | Taste receptors T2R |
| T2002 | Q697L4 | Taste receptor type 2 | Taste receptors T2R |
| T2002 | Q697L2 | Taste receptor type 2 | Taste receptors T2R |
| T2002 | Q646E9 | Taste receptor type 2 | Taste receptors T2R |
| T2002 | Q697L3 | Taste receptor type 2 | Taste receptors T2R |
| T2003 | Q7RTR8 | Taste receptor type 2 | Taste receptors T2R |
| T2003 | Q646G3 | Taste receptor type 2 | Taste receptors T2R |
| T2003 | Q646B8 | Taste receptor type 2 | Taste receptors T2R |
| T2003 | Q645U9 | Taste receptor type 2 | Taste receptors T2R |
| T2003 | Q5Y4Z2 | Taste receptor type 2 | Taste receptors T2R |
| T2003 | Q645Z3 | Taste receptor type 2 | Taste receptors T2R |
| T2004 | Q9NYW3 | Taste receptor type 2 | Taste receptors T2R |
| T2004 | Q646F6 | Taste receptor type 2 | Taste receptors T2R |
| T2004 | Q646A2 | Taste receptor type 2 | Taste receptors T2R |
| T2004 | Q645V8 | Taste receptor type 2 | Taste receptors T2R |
| T2004 | Q645T7 | Taste receptor type 2 | Taste receptors T2R |
| T2004 | Q646D6 | Taste receptor type 2 | Taste receptors T2R |
| T2005 | Q9NYW0 | Taste receptor type 2 | Taste receptors T2R |
| T2005 | Q646F5 | Taste receptor type 2 | Taste receptors T2R |
| T2005 | Q646B5 | Taste receptor type 2 | Taste receptors T2R |
| T2005 | Q646D7 | Taste receptor type 2 | Taste receptors T2R |
| T2005 | Q645V0 | Taste receptor type 2 | Taste receptors T2R |
| T2006 | Q9NYW4 | Taste receptor type 2 | Taste receptors T2R |
| T2006 | Q646D5 | Taste receptor type 2 | Taste receptors T2R |
| T2006 | Q646E6 | Taste receptor type 2 | Taste receptors T2R |
| T2006 | Q645Z1 | Taste receptor type 2 | Taste receptors T2R |
| T2006 | Q645U0 | Taste receptor type 2 | Taste receptors T2R |
| T2007 | P59544 | Taste receptor type 2 | Taste receptors T2R |
| T2007 | Q646C3 | Taste receptor type 2 | Taste receptors T2R |
| T2007 | Q646E4 | Taste receptor type 2 | Taste receptors T2R |
| T2007 | Q646A1 | Taste receptor type 2 | Taste receptors T2R |
| T2007 | Q645V7 | Taste receptor type 2 | Taste receptors T2R |
| T2008 | P59535 | Taste receptor type 2 | Taste receptors T2R |
| T2008 | Q646B1 | Taste receptor type 2 | Taste receptors T2R |
| T2008 | Q645Y9 | Taste receptor type 2 | Taste receptors T2R |
| T2008 | Q645U5 | Taste receptor type 2 | Taste receptors T2R |
| T2008 | Q646D4 | Taste receptor type 2 | Taste receptors T2R |
| T2009 | Q7M707 | Taste receptor type 2 | Taste receptors T2R |
| T2009 | Q7M715 | Taste receptor type 2 | Taste receptors T2R |
| T2009 | Q675B9 | Taste receptor type 2 | Taste receptors T2R |
| T2009 | Q675B8 | Taste receptor type 2 | Taste receptors T2R |
| T2010 | Q646E2 | Taste receptor type 2 | Taste receptors T2R |
| T2010 | Q646C1 | Taste receptor type 2 | Taste receptors T2R |
| T2010 | Q645V4 | Taste receptor type 2 | Taste receptors T2R |
| T2010 | P59541 | Taste receptor type 2 | Taste receptors T2R |
| T2011 | P59534 | Taste receptor type 2 | Taste receptors T2R |
| T2011 | Q646A9 | Taste receptor type 2 | Taste receptors T2R |
| T2011 | Q646C8 | Taste receptor type 2 | Taste receptors T2R |
| T2011 | Q645S5 | Taste receptor type 2 | Taste receptors T2R |
| T2012 | Q9NYV7 | Taste receptor type 2 | Taste receptors T2R |
| T2012 | Q646B3 | Taste receptor type 2 | Taste receptors T2R |
| T2012 | Q646E7 | Taste receptor type 2 | Taste receptors T2R |
| T2012 | Q646D1 | Taste receptor type 2 | Taste receptors T2R |
| T2013 | Q9NYV9 | Taste receptor type 2 | Taste receptors T2R |
| T2013 | Q646B6 | Taste receptor type 2 | Taste receptors T2R |
| T2013 | Q646D8 | Taste receptor type 2 | Taste receptors T2R |
| T2013 | Q645V1 | Taste receptor type 2 | Taste receptors T2R |
| T2014 | Q9NYW5 | Taste receptor type 2 | Taste receptors T2R |
| T2014 | Q646F1 | Taste receptor type 2 | Taste receptors T2R |
| T2014 | Q645Y8 | Taste receptor type 2 | Taste receptors T2R |
| T2014 | Q646D3 | Taste receptor type 2 | Taste receptors T2R |
| T2015 | Q9NYW6 | Taste receptor type 2 | Taste receptors T2R |
| T2015 | Q645Y5 | Taste receptor type 2 | Taste receptors T2R |
| T2015 | Q646A7 | Taste receptor type 2 | Taste receptors T2R |
| T2015 | Q646D2 | Taste receptor type 2 | Taste receptors T2R |
| T2016 | Q9NYW7 | Taste receptor type 2 | Taste receptors T2R |
| T2016 | Q8MJU6 | Taste receptor type 2 | Taste receptors T2R |
| T2016 | Q646G9 | Taste receptor type 2 | Taste receptors T2R |
| T2016 | Q646H0 | Taste receptor type 2 | Taste receptors T2R |
| T2017 | P59542 | Taste receptor type 2 | Taste receptors T2R |
| T2017 | Q645Z9 | Taste receptor type 2 | Taste receptors T2R |
| T2017 | Q5Y4Z5 | Taste receptor type 2 | Taste receptors T2R |
| T2017 | Q5Y4Y9 | Taste receptor type 2 | Taste receptors T2R |
| T2018 | Q7TQB0 | Taste receptor type 2 | Taste receptors T2R |
| T2018 | Q67ES7 | Taste receptor type 2 | Taste receptors T2R |
| T2018 | Q5Y4Z0 | Taste receptor type 2 | Taste receptors T2R |
| T2019 | P59538 | Taste receptor type 2 | Taste receptors T2R |
| T2019 | Q646B9 | Taste receptor type 2 | Taste receptors T2R |
| T2019 | Q645V3 | Taste receptor type 2 | Taste receptors T2R |
| T2020 | Q646A5 | Taste receptor type 2 | Taste receptors T2R |
| T2020 | Q645U7 | Taste receptor type 2 | Taste receptors T2R |
| T2020 | Q646D0 | Taste receptor type 2 | Taste receptors T2R |
| T2021 | Q9NYV8 | Taste receptor type 2 | Taste receptors T2R |
| T2021 | Q646D9 | Taste receptor type 2 | Taste receptors T2R |
| T2021 | Q645V2 | Taste receptor type 2 | Taste receptors T2R |
| T2022 | Q9NYW2 | Taste receptor type 2 | Taste receptors T2R |
| T2022 | Q646A3 | Taste receptor type 2 | Taste receptors T2R |
| T2022 | Q645U8 | Taste receptor type 2 | Taste receptors T2R |
| T2023 | P59536 | Taste receptor type 2 | Taste receptors T2R |
| T2023 | Q646B2 | Taste receptor type 2 | Taste receptors T2R |
| T2023 | Q646C7 | Taste receptor type 2 | Taste receptors T2R |
| T2024 | P59543 | Taste receptor type 2 | Taste receptors T2R |
| T2024 | Q646E3 | Taste receptor type 2 | Taste receptors T2R |
| T2024 | Q646A0 | Taste receptor type 2 | Taste receptors T2R |
| T2025 | P59537 | Taste receptor type 2 | Taste receptors T2R |
| T2025 | Q646B4 | Taste receptor type 2 | Taste receptors T2R |
| T2025 | Q5Y500 | Taste receptor type 2 | Taste receptors T2R |
| T2026 | Q7M713 | Taste receptor type 2 | Taste receptors T2R |
| T2026 | Q67ER8 | Taste receptor type 2 | Taste receptors T2R |
| T2027 | Q7TQA8 | Taste receptor type 2 | Taste receptors T2R |
| T2027 | Q675B7 | Taste receptor type 2 | Taste receptors T2R |
| T2028 | Q7M710 | Taste receptor type 2 | Taste receptors T2R |
| T2028 | Q67ET4 | Taste receptor type 2 | Taste receptors T2R |
| T2029 | Q9JKA3 | Taste receptor type 2 | Taste receptors T2R |
| T2029 | Q67ET5 | Taste receptor type 2 | Taste receptors T2R |
| T2030 | Q7TQA4 | Taste receptor type 2 | Taste receptors T2R |
| T2030 | Q67ES0 | Taste receptor type 2 | Taste receptors T2R |
| T2031 | Q7M711 | Taste receptor type 2 | Taste receptors T2R |
| T2031 | Q67ES1 | Taste receptor type 2 | Taste receptors T2R |
| T2032 | Q7M709 | Taste receptor type 2 | Taste receptors T2R |
| T2032 | Q67ES6 | Taste receptor type 2 | Taste receptors T2R |
| T2033 | Q7M724 | Taste receptor type 2 | Taste receptors T2R |
| T2033 | Q67ET1 | Taste receptor type 2 | Taste receptors T2R |
| T2034 | Q7M721 | Taste receptor type 2 | Taste receptors T2R |
| T2034 | Q67ET3 | Taste receptor type 2 | Taste receptors T2R |
| T2035 | Q7M717 | Taste receptor type 2 | Taste receptors T2R |
| T2035 | Q675C0 | Taste receptor type 2 | Taste receptors T2R |
| T2036 | Q9JKT7 | Taste receptor type 2 | Taste receptors T2R |
| T2036 | Q7M720 | Taste receptor type 2 | Taste receptors T2R |
| T2037 | Q9JKT8 | Taste receptor type 2 | Taste receptors T2R |
| T2037 | Q7M722 | Taste receptor type 2 | Taste receptors T2R |
| T2038 | Q9JKT4 | Taste receptor type 2 | Taste receptors T2R |
| T2038 | Q9JKT5 | Taste receptor type 2 | Taste receptors T2R |
| T2039 | Q9JKF0 | Taste receptor type 2 | Taste receptors T2R |
| T2039 | P59528 | Taste receptor type 2 | Taste receptors T2R |
| T2040 | Q7TQA5 | Taste receptor type 2 | Taste receptors T2R |
| T2040 | Q67ER9 | Taste receptor type 2 | Taste receptors T2R |
| T2041 | Q7M718 | Taste receptor type 2 | Taste receptors T2R |
| T2041 | Q67ES5 | Taste receptor type 2 | Taste receptors T2R |
| T2042 | Q7M712 | Taste receptor type 2 | Taste receptors T2R |
| T2042 | Q9JKE8 | Taste receptor type 2 | Taste receptors T2R |
| T2043 | Q9JKT3 | Taste receptor type 2 | Taste receptors T2R |
| T2043 | Q67ET0 | Taste receptor type 2 | Taste receptors T2R |
| T2044 | Q7TQB9 | Taste receptor type 2 | Taste receptors T2R |
| T2044 | Q67ES3 | Taste receptor type 2 | Taste receptors T2R |
| T2045 | Q7TQA7 | Taste receptor type 2 | Taste receptors T2R |
| T2045 | Q67ET7 | Taste receptor type 2 | Taste receptors T2R |
| T2046 | Q7M725 | Taste receptor type 2 | Taste receptors T2R |
| T2046 | Q9JKT9 | Taste receptor type 2 | Taste receptors T2R |
| T2047 | Q7M723 | Taste receptor type 2 | Taste receptors T2R |
| T2047 | Q67ES9 | Taste receptor type 2 | Taste receptors T2R |
| T2048 | Q9NYW1 | Taste receptor type 2 | Taste receptors T2R |
| T2048 | Q645T0 | Taste receptor type 2 | Taste receptors T2R |
| T2049 | Q646F4 | Taste receptor type 2 | Taste receptors T2R |
| T2049 | Q645T2 | Taste receptor type 2 | Taste receptors T2R |
| T2050 | Q7TQB8 | Taste receptor type 2 | Taste receptors T2R |
| T2050 | Q67ET2 | Taste receptor type 2 | Taste receptors T2R |
| T2051 | Q9JKE7 | Taste receptor type 2 | Taste receptors T2R |
| T2051 | P59532 | Taste receptor type 2 | Taste receptors T2R |
| T2052 | Q9JKT2 | Taste receptor type 2 | Taste receptors T2R |
| T2052 | Q9JKU1 | Taste receptor type 2 | Taste receptors T2R |
| T2053 | Q646F8 | Taste receptor type 2 | Taste receptors T2R |
| T2053 | Q645T3 | Taste receptor type 2 | Taste receptors T2R |
| T2054 | Q645T6 | Taste receptor type 2 | Taste receptors T2R |
| T2054 | Q646G2 | Taste receptor type 2 | Taste receptors T2R |
| T2055 | Q7TQA9 | Taste receptor type 2 | Taste receptors T2R |
| T2055 | Q67ES2 | Taste receptor type 2 | Taste receptors T2R |
| T2056 | Q7TQA6 | Taste receptor type 2 | Taste receptors T2R |
| T2056 | Q4VHE7 | Taste receptor type 2 | Taste receptors T2R |
| T2057 | P59530 | Taste receptor type 2 | Taste receptors T2R |
| T2057 | Q9JKE9 | Taste receptor type 2 | Taste receptors T2R |
| Tb001 | Q8IZF7 | To be sorted | To be sorted |
| Tb001 | Q8IZF3 | To be sorted | To be sorted |
| Tb001 | Q9D2L6 | To be sorted | To be sorted |
| Tb002 | Q9WVT0 | To be sorted | To be sorted |
| Tb002 | Q8IZF2 | To be sorted | To be sorted |
| Tb003 | Q6JAN0 | To be sorted | To be sorted |
| Tb003 | Q8VHN7 | To be sorted | To be sorted |
| Tr001 | O08725 | Thyrotropin-releasing hormone receptor | Growth hormone secretagogue |
| Tr001 | O43193 | Thyrotropin-releasing hormone receptor | Growth hormone secretagogue like |
| Tr001 | Q99P50 | Thyrotropin-releasing hormone receptor | Growth hormone secretagogue |
| Tr001 | A5A4L1 | Thyrotropin-releasing hormone receptor | Growth hormone secretagogue |
| Tr001 | A5A4K9 | Thyrotropin-releasing hormone receptor | Growth hormone secretagogue |
| Tr001 | Q92847 | Thyrotropin-releasing hormone receptor | Growth hormone secretagogue |
| Tr001 | Q95254 | Thyrotropin-releasing hormone receptor | Growth hormone secretagogue |
| Tr002 | P21761 | Thyrotropin-releasing hormone receptor | Thyrotropin-releasing hormone |
| Tr002 | Q01717 | Thyrotropin-releasing hormone receptor | Thyrotropin-releasing hormone |
| Tr002 | O46639 | Thyrotropin-releasing hormone receptor | Thyrotropin-releasing hormone |
| Tr002 | P34981 | Thyrotropin-releasing hormone receptor | Thyrotropin-releasing hormone |
| Tr002 | O93603 | Thyrotropin-releasing hormone receptor | Thyrotropin-releasing hormone |
| Tr002 | Q28596 | Thyrotropin-releasing hormone receptor | Thyrotropin-releasing hormone |
| VA001 | Q9EQ48 | Vomeronasal type-1 receptor A | Vomeronasal receptors V1RA |
| VA001 | Q9EP79 | Vomeronasal type-1 receptor A | Vomeronasal receptors V1RA |
| VA001 | Q5J3G9 | Vomeronasal type-1 receptor A | Vomeronasal receptors V1RA |
| VA001 | Q5J3E5 | Vomeronasal type-1 receptor A | Vomeronasal receptors V1RA |
| VA002 | Q9EPB8 | Vomeronasal type-1 receptor A | Vomeronasal receptors V1RA |
| VA002 | Q9EQ51 | Vomeronasal type-1 receptor A | Vomeronasal receptors V1RA |
| VA002 | Q9EQ52 | Vomeronasal type-1 receptor A | Vomeronasal receptors V1RA |
| VA003 | Q5J3K9 | Vomeronasal type-1 receptor A | Vomeronasal receptors V1RA |
| VA003 | Q5J3K5 | Vomeronasal type-1 receptor A | Vomeronasal receptors V1RA |
| VA003 | Q62850 | Vomeronasal type-1 receptor A | Vomeronasal receptors V1RA |
| VA004 | Q8VIC7 | Vomeronasal type-1 receptor A | Vomeronasal receptors V1RA |
| VA004 | Q8R2E6 | Vomeronasal type-1 receptor A | Vomeronasal receptors V1RA |
| VA004 | Q8VIC6 | Vomeronasal type-1 receptor A | Vomeronasal receptors V1RA |
| VA005 | Q8VIC9 | Vomeronasal type-1 receptor A | Vomeronasal receptors V1RA |
| VA005 | Q8VBS7 | Vomeronasal type-1 receptor A | Vomeronasal receptors V1RA |
| VB001 | Q9WUF1 | Vomeronasal type-1 receptor B | Vomeronasal receptors V1RB |
| VB001 | Q9EQ45 | Vomeronasal type-1 receptor B | Vomeronasal receptors V1RB |
| VB001 | Q9EP51 | Vomeronasal type-1 receptor B | Vomeronasal receptors V1RB |
| VB001 | Q9EQ47 | Vomeronasal type-1 receptor B | Vomeronasal receptors V1RB |
| VB001 | Q9EP93 | Vomeronasal type-1 receptor B | Vomeronasal receptors V1RB |
| VB002 | Q9EQ46 | Vomeronasal type-1 receptor B | Vomeronasal receptors V1RB |
| VB002 | Q9EQ44 | Vomeronasal type-1 receptor B | Vomeronasal receptors V1RB |
| VB002 | Q5J3M9 | Vomeronasal type-1 receptor B | Vomeronasal receptors V1RB |
| VB003 | Q5J3L7 | Vomeronasal type-1 receptor B | Vomeronasal receptors V1RB |
| VB003 | Q5J3N1 | Vomeronasal type-1 receptor B | Vomeronasal receptors V1RB |
| VB004 | Q5J3M3 | Vomeronasal type-1 receptor B | Vomeronasal receptors V1RB |
| VB004 | Q5J3L4 | Vomeronasal type-1 receptor B | Vomeronasal receptors V1RB |
| Vi001 | Q91085 | Vasoactive intestinal polypeptide receptor | Vasoactive intestinal polypeptide |
| Vi001 | Q90308 | Vasoactive intestinal polypeptide receptor | Vasoactive intestinal polypeptide |
| Vi001 | P97751 | Vasoactive intestinal polypeptide receptor | Vasoactive intestinal polypeptide |
| Vi001 | P32241 | Vasoactive intestinal polypeptide receptor | Vasoactive intestinal polypeptide |
| Vi001 | P30083 | Vasoactive intestinal polypeptide receptor | Vasoactive intestinal polypeptide |
| Vi001 | Q28992 | Vasoactive intestinal polypeptide receptor | Vasoactive intestinal polypeptide |
| Vi002 | P41588 | Vasoactive intestinal polypeptide receptor | Vasoactive intestinal polypeptide |
| Vi002 | P41587 | Vasoactive intestinal polypeptide receptor | Vasoactive intestinal polypeptide |
| Vi002 | P35000 | Vasoactive intestinal polypeptide receptor | Vasoactive intestinal polypeptide |
| Vi003 | P69332 | Viral receptor | US28 |
| Vi003 | P69333 | Viral receptor | US28 |
| VL001 | Q9GZP7 | Vomeronasal type-1 receptor L | Vomeronasal receptors V1RL |
| VL001 | Q8WN92 | Vomeronasal type-1 receptor L | Vomeronasal receptors V1RL |
| VL002 | Q7YRP3 | Vomeronasal type-1 receptor L | Vomeronasal receptors V1RL |
| VL002 | Q9BXE9 | Vomeronasal type-1 receptor L | Vomeronasal receptors V1RL |
| Vo001 | Q7Z5H4 | Vomeronasal type-1 receptor other | Vomeronasal receptors other |
| Vo001 | Q7YRP1 | Vomeronasal type-1 receptor other | Vomeronasal receptors other |
| outlier | Q19084 | Aminergic receptor | Serotonin type 1a |
| outlier | Q03613 | Peptide receptor | GPR74 like |
| outlier | Q6IF63 | Olfactory receptor | Olfactory II fam 12 / MOR250 |
| outlier | Q09638 | Peptide receptor | GPR74 like |
| outlier | Q24563 | Aminergic receptor | Dopamine Insect type 2 |
| outlier | Q66673 | Peptide receptor | C-C Chemokine type 3 |
| outlier | P25931 | Peptide receptor | Tachykinin like 2 |
| outlier | Q8NGR6 | Olfactory receptor | Olfactory 257 |
| outlier | Q8N684 | Class A Orphan/other | GPR88 |
| outlier | Q5W9T5 | Peptide receptor | Vasotocin |
| outlier | P20905 | Aminergic receptor | Serotonin type 1a |
| outlier | Q09388 | Aminergic receptor | Musc. acetylcholine Non-Vertebrate |
| outlier | A6NFC9 | Olfactory receptor | Olfactory 73 |
| outlier | Q8N148 | Olfactory receptor | Olfactory 216 |
| outlier | Q75W84 | Peptide receptor | Annetocin |
| outlier | Q7YW31 | Peptide receptor | Vasotocin |
| outlier | Q8NH09 | Olfactory receptor | Olfactory 85 |
| outlier | Q9GZK7 | Olfactory receptor | Olfactory II fam 3 / MOR255 |
| outlier | Q868T3 | Peptide receptor | Vasotocin |
| outlier | Q2V2K5 | Peptide receptor | Vasotocin |
| outlier | Q9VZW5 | Peptide receptor | Neuropeptide Y / peptide YY |
| outlier | Q18007 | Aminergic receptor | Musc. acetylcholine Non-Vertebrate |
| outlier | Q9H342 | Olfactory receptor | Olfactory 243 |
| outlier | O01670 | Aminergic receptor | Alpha Adrenoceptors type 2c |
| outlier | Q8WPA2 | Peptide receptor | Allostatin |
| outlier | Q9Y2T6 | Class A Orphan/other | Other 519 |
| outlier | P41596 | Aminergic receptor | Dopamine Insect type 1 |
| outlier | Q5WA50 | Peptide receptor | Vasotocin |
| outlier | Q11082 | Peptide receptor | Galanin type 1 |
| outlier | Q8NG92 | Olfactory receptor | Olfactory II fam 3 / MOR255 |
| outlier | A6NND4 | Olfactory receptor | Olfactory II fam 3 / MOR255 |
| outlier | Q8IS44 | Aminergic receptor | Dopamine Vertebrate type 2 |
| outlier | Q8NH95 | Olfactory receptor | Olfactory 95 |
| outlier | P52380 | Peptide receptor | C-C Chemokine type 3 |
| outlier | Q86917 | Peptide receptor | C-C Chemokine type 8 |
| outlier | Q9J5H4 | Peptide receptor | Fmet-leu-phe |
| outlier | P34311 | Peptide receptor | GPR74 like |
| outlier | Q9J529 | Class A Orphan/other | EBV-induced |
| outlier | Q03566 | Peptide receptor | Substance P (NK1) |
| outlier | Q6SW98 | Peptide receptor | C-C Chemokine type 11 |
| outlier | O15974 | (Rhod)opsin receptor | G0 coupled Rhodopsin Mollusc |
| outlier | Q96G91 | Nucleotide receptor | P2RY11 |
| outlier | Q9J5I0 | Peptide receptor | Fmet-leu-phe |
| outlier | Q8NGJ8 | Olfactory receptor | Olfactory II fam 10 / MOR263-269 |
| outlier | Q18775 | Aminergic receptor | Serotonin type 1a |
| outlier | Q8NGE0 | Olfactory receptor | Olfactory 184 |
| outlier | P09703 | Viral receptor | US27 |
| outlier | Q96CH1 | Class A Orphan/other | GPR139 |
| outlier | P16395 | Aminergic receptor | Musc. acetylcholine Non-Vertebrate 2 |
| outlier | Q8TDS5 | Nucleotide receptor | P2RY5 |
| outlier | Q9NDM2 | Peptide receptor | Melanin-concentrating hormone receptors |
| outlier | Q09502 | Peptide receptor | Prolactin-releasing peptide (GPR10) |
| outlier | Q8ITC7 | Neuropeptides capa receptor | CAPA |
| outlier | Q9DDD1 | Class A Orphan/other | GPR139 |
| outlier | Q8NH08 | Olfactory receptor | Olfactory II fam 3 / MOR255 |
| outlier | P35410 | Class A Orphan/other | Mas proto-oncogene and Mas-related (MRGs) |
| outlier | Q9VNM1 | Peptide receptor | Neuropeptide Y type 1 |
| outlier | O42179 | Peptide receptor | Somatostatin type 5 |
| outlier | O77408 | Aminergic receptor | Alpha Adrenoceptors type 2a |
| outlier | Q8TDV2 | Class A Orphan/other | GPR139 |
| outlier | Q9W0R6 | Methuselah-like proteins | Methuselah-like proteins (MTH) type 3 |
| outlier | Q9W4Y2 | Pigment-dispersing factor receptor | PDF |
| outlier | Q7Z7M1 | GPR114 | GPR114 |
| outlier | Q09460 | Calcitonin receptor | Calcitonin |

Table S2. Normalized root mean square deviation (NRMSD) in the sequence distances of 10 query proteins to the 2841 base sequences using the base dataset and the base + query dataset.

| **UniProt ID** | **Protein Name** | **Organism** | **Length (a. a.)** | **NRMSD** |
| --- | --- | --- | --- | --- |
| B3H7A6 | S-protein homolog 19 | Arabidopsis thaliana | 132 | 1.92E-06 |
| A0A088MIT0 | Bradykinin-related peptides | Physalaemus nattereri | 134 | 1.95E-06 |
| P0DN81 | Olfactory receptor 13C7 | Homo sapiens | 318 | 3.89E-06 |
| P0DMS8 | Adenosine receptor A3 | Homo sapiens | 318 | 3.89E-06 |
| A1ZAX0 | Neuropeptide CCHamide-1 receptor | D. melanogaster | 499 | 5.10E-06 |
| P03878 | Intron-encoded DNA endonuclease aI4 | S. cerevisiae | 556 | 5.61E-06 |
| G5ECX0 | Latrophilin-like protein LAT-2 | C. elegans | 1338 | 9.08E-06 |
| S4X0Q8 | Adhesion G protein-coupled receptor A3 | Danio rerio | 1346 | 9.09E-06 |
| P36022 | Dynein heavy chain, cytoplasmic | S. cerevisiae | 4092 | 1.58E-05 |
| Q19020 | Dynein heavy chain, cytoplasmic | C. elegans | 4568 | 1.66E-05 |

Table S3. BLASTp based GPCR detection for 100 tested protein sequences, including 46 GPCRs, 25 membrane proteins (MP), and 29 7TM non-GPCRs (7nG). The sequence in red is falsely predicted to be a non-GPCR due to an incomplete base dataset.

| **UniProt ID** | **Closest GPCR ID** | **Sequence distance** | **Annotation** | **Prediction** |
| --- | --- | --- | --- | --- |
| Q6IF36 | Q15614 | 0 | GPCR | GPCR |
| P0DN80 | A6NKK0 | 0 | GPCR | GPCR |
| P0DN82 | P58182 | 0 | GPCR | GPCR |
| P0DMU2 | Q15614 | 0 | GPCR | GPCR |
| Q9TUA1 | Q9TQX4 | 0 | GPCR | GPCR |
| Q864G0 | Q864J3 | 0 | GPCR | GPCR |
| P0DMS8 | P33765 | 0 | GPCR | GPCR |
| P0DN81 | Q9NQN1 | 0 | GPCR | GPCR |
| P22671 | Q98980 | 0 | GPCR | GPCR |
| P0DN77 | P04001 | 0 | GPCR | GPCR |
| P0DN78 | P04001 | 0 | GPCR | GPCR |
| Q8C4G9 | Q86SQ6 | 0 | GPCR | GPCR |
| D4A3T6 | Q8IZF7 | 0 | GPCR | GPCR |
| E9Q4J9 | Q8IZF7 | 0 | GPCR | GPCR |
| Q7SY09 | Q923X1 | 0 | GPCR | GPCR |
| H2L0Q3 | Q9WV18 | 0 | GPCR | GPCR |
| S4X0Q8 | Q8IWK6 | 0 | GPCR | GPCR |
| G5E8Q8 | Q9WVT0 | 0 | GPCR | GPCR |
| E7FBY6 | Q91ZV8 | 0 | GPCR | GPCR |
| C0HL12 | Q3UHD1 | 0 | GPCR | GPCR |
| P02699 | P02699 | 0 | GPCR | GPCR |
| G3M4F8 | Q4LBB9 | 1.43E-156 | GPCR | GPCR |
| G5ECB2 | O75899 | 6.30E-155 | GPCR | GPCR |
| A0A0K3AWM6 | Q8AVJ9 | 6.74E-136 | GPCR | GPCR |
| G4WMX4 | P25931 | 2.88E-115 | GPCR | GPCR |
| C3ZQF9 | Q96P65 | 2.03E-107 | GPCR | GPCR |
| G5ECX0 | O95490 | 3.14E-104 | GPCR | GPCR |
| Q9JKU0 | Q646B3 | 3.11E-101 | GPCR | GPCR |
| Q7JQF1 | O77408 | 1.55E-72 | GPCR | GPCR |
| E9QJ73 | Q5MD61 | 1.94E-72 | GPCR | GPCR |
| Q23497 | Q8WPA2 | 2.26E-69 | GPCR | GPCR |
| Q4V622 | P47751 | 3.27E-66 | GPCR | GPCR |
| Q2AB83 | Q645Y9 | 6.21E-65 | GPCR | GPCR |
| A1ZAX0 | P21729 | 1.21E-63 | GPCR | GPCR |
| Q8ITC9 | Q8ITC7 | 1.28E-61 | GPCR | GPCR |
| Q8IN35 | Q9Y5X5 | 1.13E-59 | GPCR | GPCR |
| Q59E83 | P25931 | 9.66E-48 | GPCR | GPCR |
| O02300 | Q7YW31 | 1.36E-39 | GPCR | GPCR |
| Q18321 | Q9VZW5 | 5.15E-38 | GPCR | GPCR |
| O62169 | Q7YW31 | 5.85E-35 | GPCR | GPCR |
| Q9VML9 | P56719 | 1.80E-28 | GPCR | GPCR |
| Q9U320 | Q62463 | 3.34E-25 | GPCR | GPCR |
| Q8BGT1 | O88279 | 2.36E-21 | GPCR | GPCR |
| Q8SWR3 | Q9VZW5 | 5.24E-21 | GPCR | GPCR |
| G5ECD9 | P46002 | 1.20E-08 | GPCR | GPCR |
| B3DH96 | Q8NGB4 | 1.53E-07 | GPCR | GPCR |
| O45767 | P47747 | 0.002 | GPCR | Non-GPCR |
| Q12117 | Q2WED0 | 0.005 | 7nG | Non-GPCR |
| P0DMH8 | B7ZCC9 | 0.018 | 7nG | Non-GPCR |
| Q8LD98 | Q9XT57 | 0.02 | MP | Non-GPCR |
| P38079 | Q25190 | 0.026 | 7nG | Non-GPCR |
| Q9HNE5 | P30968 | 0.045 | 7nG | Non-GPCR |
| Q9HNE6 | Q2KNE5 | 0.099 | 7nG | Non-GPCR |
| Q57101 | P56442 | 0.1 | 7nG | Non-GPCR |
| P02945 | Q95NC9 | 0.11 | 7nG | Non-GPCR |
| Q8GUJ1 | F8VQN3 | 0.12 | MP | Non-GPCR |
| E2RQ08 | Q8NG98 | 0.19 | MP | Non-GPCR |
| Q5UXY6 | P56442 | 0.21 | 7nG | Non-GPCR |
| O74631 | O75899 | 0.21 | 7nG | Non-GPCR |
| O93740 | Q6F3F9 | 0.26 | 7nG | Non-GPCR |
| Q9HPU7 | O60241 | 0.37 | 7nG | Non-GPCR |
| A0A2R4S913 | P46090 | 0.4 | 7nG | Non-GPCR |
| Q8L636 | P34981 | 0.47 | MP | Non-GPCR |
| P96787 | D4AC13 | 0.49 | 7nG | Non-GPCR |
| A0A0K8P8E7 | Q9Z2H4 | 0.54 | MP | Non-GPCR |
| P29563 | P56483 | 0.6 | 7nG | Non-GPCR |
| F4I3V6 | Q13255 | 0.61 | MP | Non-GPCR |
| Q9SSS9 | Q2WED0 | 0.65 | MP | Non-GPCR |
| G3V7W1 | Q5XXP2 | 0.67 | MP | Non-GPCR |
| Q9V3J4 | Q9VSE7 | 0.76 | MP | Non-GPCR |
| Q5V4H7 | Q15619 | 0.85 | 7nG | Non-GPCR |
| Q5QNI2 | P23271 | 0.97 | MP | Non-GPCR |
| F1PJP5 | Q8MJ08 | 1 | MP | Non-GPCR |
| Q6ZIV7 | Q54P68 | 1.1 | MP | Non-GPCR |
| Q9AYU1 | Q8NGE1 | 1.1 | MP | Non-GPCR |
| F1PCT7 | Q6JAN0 | 1.1 | MP | Non-GPCR |
| Q5V5V3 | Q3UVX5 | 1.1 | 7nG | Non-GPCR |
| Q9FI83 | O00398 | 1.2 | MP | Non-GPCR |
| Q9AFF7 | Q9WVT0 | 1.2 | 7nG | Non-GPCR |
| P29255 | O60883 | 1.5 | MP | Non-GPCR |
| P71411 | Q9J5H4 | 1.5 | 7nG | Non-GPCR |
| F2Q9V4 | P79266 | 1.8 | MP | Non-GPCR |
| Q6K3R5 | O61303 | 1.8 | MP | Non-GPCR |
| Q9S7M0 | Q2V2K5 | 1.9 | MP | Non-GPCR |
| Q9FJ04 | Q924H0 | 2.1 | MP | Non-GPCR |
| Q18DH5 | Q5T601 | 2.2 | 7nG | Non-GPCR |
| B0R2U4 | Q7RTX1 | 2.5 | 7nG | Non-GPCR |
| Q9UW81 | Q6IF82 | 3.3 | 7nG | Non-GPCR |
| Q5V0R5 | Q9UBS5 | 3.6 | 7nG | Non-GPCR |
| Q5V1N0 | Q864H3 | 3.6 | 7nG | Non-GPCR |
| B0R367 | P23944 | 3.9 | 7nG | Non-GPCR |
| A3PDP9 | Q8MJ08 | 4 | MP | Non-GPCR |
| Q9F7P4 | Q9WVT0 | 4.4 | 7nG | Non-GPCR |
| P15647 | Q9U7D5 | 6.5 | 7nG | Non-GPCR |
| B3H7A6 | Q14246 | 7.5 | MP | Non-GPCR |
| G0LFX8 | Q4QXU2 | 7.8 | 7nG | Non-GPCR |
| Q18DH8 | Q4QXU2 | 7.8 | 7nG | Non-GPCR |
| P0DOE6 | Q6IF99 | 8.9 | MP | Non-GPCR |
| P0DOE7 | Q6IF99 | 8.9 | MP | Non-GPCR |
| Q5UXM9 | A6NM76 | 9.6 | 7nG | Non-GPCR |

Table S4. Common GO annotations of G3M4F8 and Q4LBB9 in their molecular functions, biological processes, and cellular components.

| Molecular function | Biological process | Cellular Component |
| --- | --- | --- |
| GO:0004930  G-protein coupled receptor activity | GO:0007165  signal transduction | GO:0005622  intracellular |
| GO:0004989  octopamine receptor activity | GO:0007186  G-protein coupled receptor signaling pathway | GO:0005886  plasma membrane |
|  | GO:0007189  adenylate cyclase-activating G-protein coupled receptor signaling pathway | GO:0016020  membrane |
|  |  | GO:0016021  integral component of membrane |

Table S5. Detection of Q13324 and P34998 isoforms using SeQuery. Q13324 is human Corticotropin-releasing hormone receptor 2 (CRHR2) and P34998 is human Corticotropin-releasing hormone receptor 1 (CRHR1). The last eight sequences are computationally mapped potential isoforms of P34998.

| **CRHR2 isoforms** | **Length** | **Closest sequence** | **Organism** | **Percent identity** | **Fit length ratio** | **Sequence distance** |
| --- | --- | --- | --- | --- | --- | --- |
| Q13324-1 | 411 | Q13324 | Homo sapiens | 100% | 100% | 0 |
| Q13324-2 | 438 | Q13324 | Homo sapiens | 99.74% | 86.30% | 0 |
| Q13324-3 | 397 | Q13324 | Homo sapiens | 98.86% | 96.47% | 0 |
| Q13324-4 | 387 | Q13324 | Homo sapiens | 100% | 94.32% | 0 |
| Q13324-5 | 370 | Q13324 | Homo sapiens | 100% | 98.65% | 0 |
| Q13324-6 | 366 | Q13324 | Homo sapiens | 100% | 100% | 0 |
| Q13324-7 | 410 | Q13324 | Homo sapiens | 99.76% | 100% | 0 |
| **CRHR1 Isoforms** |  |  |  |  |  |  |
| P34998-1 | 444 | P34998 | Homo sapiens | 100% | 100% | 0 |
| P34998-2 | 415 | Q76LL8 (P34988) | Macaca mulatta | 99.52% (91.67%) | 100%  (100%) | 0  (0) |
| P34998-3 | 375 | Q76LL8 (P34988) | Macaca mulatta | 89.64%  (82.43%) | 100%  (100%) | 0  (0) |
| P34998-4 | 401 | Q76LL8 (P34988) | Macaca mulatta | 96.15% (88.51%) | 100%  (100%) | 0  (0) |
| P34998-5 | 240 | Q76LL8 (P34988) | Macaca mulatta | 100%  (100%) | 100%  (100%) | 0  (5.2E-180) |
| A0A0A0MQZ1 | 430 | P34998 | Homo sapiens | 96.85% | 100% | 0 |
| J9JIC6 | 145 | P34998 | Homo sapiens | 100% | 99.30% | 7.9E-103 |
| K9J956 | 154 | P34998 | Homo sapiens | 66.67% | 45.45% | 3.6E-12 |
| H0YFR5 | 52 | P35353 | Rattus norvegicus | 100% | 34.60% | 3.4E-14 |
| B3TIK8 | 314 | Q76LL8 | Macaca mulatta | 99.67% | 97% | 0 |
| J3KSM0 | 152 | Q76LL8 | Macaca mulatta | 100% | 100% | 2.4E-77 |
| J3QQR1 | 93 | Q76LL8 | Macaca mulatta | 96.20% | 84.95% | 9.1E-52 |
| J3QKP8 | 75 | O62772 | Ovis aries | 94.64% | 74.67% | 7.2E-33 |
